# Review of the Quench Sensitivity of Aluminium Alloys: Analysis of the Kinetics and Nature of Quench-Induced Precipitation

**DOI:** 10.3390/ma12244083

**Published:** 2019-12-06

**Authors:** Benjamin Milkereit, Marco J. Starink, Paul A. Rometsch, Christoph Schick, Olaf Kessler

**Affiliations:** 1 Competence Centre °CALOR, Department Life, Light & Matter, University of Rostock, 18051 Rostock, Germany; christoph.schick@uni-rostock.de (C.S.); olaf.kessler@uni-rostock.de (O.K.); 2 Chair of Materials Science, University of Rostock, 18051 Rostock, Germany; 3 Materials Research Group, School of Engineering, University of Southampton, Southampton SO17 1BJ, UK; m.j.starink@soton.ac.uk; 4 Rio Tinto, Arvida Research and Development Center, Jonquiere, QC G7S 4K8, Canada; paul.rometsch@riotinto.com; 5 Polymer Physics Group, Institute of Physics, University of Rostock, 18051 Rostock, Germany; 6 A.M. Butlerov Institute of Chemistry, Kazan Federal University, Kremlevskaya 18, 420008 Kazan, Russia

**Keywords:** quench sensitivity, aluminium alloys, DSC, kinetics, quench induces precipitation, AlMgSi wrought alloys, AlZnMg wrought alloys, AlSi wrought alloys, AlCu wrought alloys, AlSiMg cast alloys

## Abstract

For aluminium alloys, precipitation strengthening is controlled by age-hardening heat treatments, including solution treatment, quenching, and ageing. In terms of technological applications, quenching is considered a critical step, because detrimental quench-induced precipitation must be avoided to exploit the full age-hardening potential of the alloy. The alloy therefore needs to be quenched faster than a critical cooling rate, but slow enough to avoid undesired distortion and residual stresses. These contrary requirements for quenching can only be aligned based on detailed knowledge of the kinetics of quench-induced precipitation. Until the beginning of the 21st century, the kinetics of relevant solid-solid phase transformations in aluminium alloys could only be estimated by ex-situ testing of different properties. Over the past ten years, significant progress has been achieved in this field of materials science, enabled by the development of highly sensitive differential scanning calorimetry (DSC) techniques. This review presents a comprehensive report on the solid-solid phase transformation kinetics in Al alloys covering precipitation and dissolution reactions during heating from different initial states, dissolution during solution annealing and to a vast extent quench-induced precipitation during continuous cooling over a dynamic cooling rate range of ten orders of magnitude. The kinetic analyses are complemented by sophisticated micro- and nano-structural analyses and continuous cooling precipitation (CCP) diagrams are derived. The measurement of enthalpies released by quench-induced precipitation as a function of the cooling rate also enables predictions of the quench sensitivities of Al alloys using physically-based models. Various alloys are compared, and general aspects of quench-induced precipitation in Al alloys are derived.


**1. Introduction**
3
**2. Measuring Techniques and Methods for the Analysis of Solid-Solid Phase Transformations**
7 *2.1. Methods for In Situ Kinetic Analysis of Solid-Solid Phase Transformations in Metals*7 *2.2. Methodology and Systematics of State-of-the-Art Kinetic DSC Analysis*9  2.2.1. Basic Concepts of DSC Measurements9  2.2.2. Key Features for In Situ Quantification of Enthalpy Changes of Solid-Solid Phase Transformation as a Function of the Scanning Rate or Time11   Excess Specific Heat Capacity11   Zero-Level Accuracy12   Large Dynamic Range14   Specific Physical Requirements for the Alloy over the Considered Dynamic range14   Metrological Aspects of a Large Dynamic Range15 *2.3. Construction Scheme for Continuous Cooling Precipitation Diagrams*18 *2.4. Methods and Systematics for Complementary Micro- and Nano-Structure Analysis*19 *2.5. Analysis of Resulting Mechanical Properties*20 *2.6. General Illustration of Results and Reading Guidelines for CCP Diagrams*21
**3. Solid-Solid Phase Transformations in Al Alloys over a Wide Dynamic Range**
22 *3.1. Heating to Solution Treatment and Isothermal Soaking*22  3.1.1. Capabilities and Limitations of DSC Heating Curve Analysis and Interpretation22  3.1.2. Achieving a Complete Solid Solution26  3.1.3. Extending the Scanning Rate by Reheating Experiments26 *3.2. Quench-Induced Precipitation during Cooling from Solution Treatment*28  3.2.1. AlSi binary Wrought Alloys28  3.2.2. 6xxx AlMgSi Wrought Alloys33  3.2.3. 7xxx AlZnMg(Cu) Wrought Alloys48  3.2.4. 2xxx AlCu(Mg) Wrought Alloys59  3.2.5. AlSiMg Cast Alloys61
**4. General Aspects of Quench-Induced Precipitation in Al Alloys**
66
**5. Kinetic Assessment of the DSC Data by Modelling**
68
**6. Application of the Derived DSC Methods to Other Alloy Systems**
71
**7. Conclusions**
74 *7.1. In Situ DSC Analysis of Solid-Solid Phase Transformations in Precipitation Hardening Alloys*74 *7.2. Continuous Heating and Solution Annealing*74 *7.3. Continuous Cooling and Analysis of Quench-Induced Precipitation*75
**Appendix A**
77
**References**
82

## 1. Introduction

Aluminium alloys are the second most important metallic materials. In 2014, the global production of steel (about 1670 Mt) amounted to about 90% of global metal production [1,2]. Of the remaining metals, aluminium represents the majority, and in 2013 and 2014, aluminium amounted to about 60% of the total tonnage of the global production of non-ferrous metals [2]. In terms of castings, Fe-based castings represented about 80% of the global production tonnage in 2016, while Al-based castings amounted to about 17%, with Cu less than 2% [3] and other metals below 1% in total. Thus, considering non-ferrous metal castings, aluminium amounted to about 85% of the global production. The importance of aluminium and its products can also be seen from the fact that the global demand for aluminium has doubled during the last ten years, reaching more than 60 Mt in 2017 [4].

However, pure aluminium has poor strength, and its application is largely limited to packaging and foil, electronic conductors, chemical process equipment and lithographic plates [5]. In order to make use of aluminium as a construction material or in applications as an engineering material for lightweight constructions, for instance aeroplanes, the strength of Al-based materials must be increased [5,6,7]. This strength increase, i.e., an increase in the resistance against plastic deformation, can be achieved by four physical mechanisms: (i) grain-boundaries; (ii) dislocations (work hardening); (iii) alloying elements in solid solution; and (iv) alloying elements precipitated in nano-sized particles [6,7,8]. To understand these mechanisms, it is essential to know that plastic deformation (at temperatures below 0.4 times the melting temperature in K, for aluminium below about 100 °C) involves the gliding of dislocations on certain lattice planes of the metallic matrix crystal. All four mechanisms that can increase the strength are therefore based on the effect of hindering dislocations from gliding. The mechanism of precipitation strengthening is typically used to achieve the highest strengths in Al-based products [5,9].

**Figure 1:**
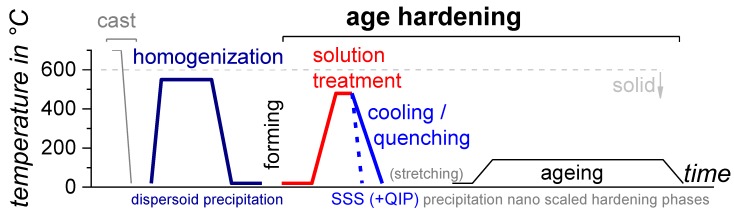
Simplified temperature-time profile of heat treatments within the production of structural Al products (SSS supersaturated solid solution, QIP quench-induced precipitates).

As seen from the above—and which is true for every engineering material—the structure of the material at micro- and nanoscales affects its properties. Thus, for several metallic alloys, changes in the inner structure due to solid-solid phase transformations control the properties. This includes not only mechanical properties such as strength, ductility and toughness, but also corrosion properties and others. The precipitation of secondary phase particles from a solid solution represents a solid-solid phase transformation. In technological applications, solid-solid phase transformations are typically adjusted by controlled heat treatments. Figure 1 shows a schematic diagram of the time-temperature profiles of some heat treatments that are relevant to the production of Al alloys. In addition to heat treatments, the production may incorporate several forming operations such as rolling or extrusion (at ambient or elevated temperatures), which substantially influence the microstructure. In addition, rolled or extruded products with relatively simple cross sections may experience a stretching treatment subsequent to the quenching process within the age-hardening treatment (prior ageing). This stretching causes relief of residual stresses induced by quenching, straightens the products and increases the dislocation density and thereby the nucleation site density for nanoscale precipitation during the final ageing.

**Figure 2:**
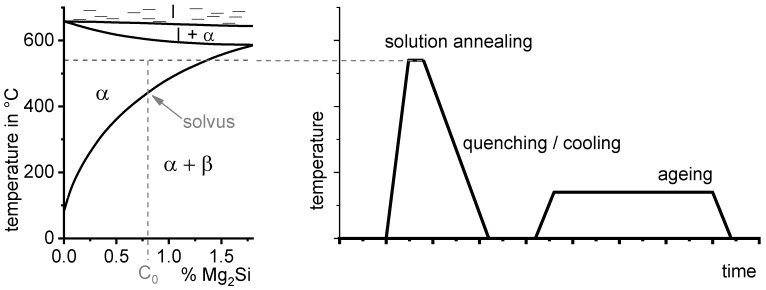
Pseudo-binary phase diagram for Al-Mg__2__Si (adapted from [5]) and schematic temperature-time profile of an age-hardening procedure.

For Al alloys, the most relevant heat treatment that influences strength is age-hardening [5]. To allow age-hardening in the first instance, alloying element atoms need to be added to the Al base material. This is mostly done in the liquid phase during casting. Age-hardening makes use of the decreasing solubility of aluminium with respect to the major alloying elements and decreasing temperature (see Figure 2). The heat treatment process of age-hardening consists of three major steps: solution treatment, quenching and ageing. The solution treatment aims to achieve a solid solution. A certain duration of soaking at solution temperature is often used to finalise the dissolution processes and/or to obtain a homogeneous distribution of the dissolved alloying element atoms within the matrix. The amount of dissolved major alloying elements typically substantially exceeds the solubility at room temperature. However, during cooling or quenching, the alloying elements need to be kept in solid solution, resulting in supersaturation of the base material. This condition is far from the thermodynamic equilibrium and is thus unstable (this is often referred to as meta-stable since the stability is relative within the timescales considered). From this unstable supersaturated solid-solution during the final ageing treatment, a high particle number density of nano-scale precipitates grows, which hinder the dislocation movement and thereby increase the strength.

The cooling step (quenching) is the most critical step in the age-hardening heat treatment process with regard to technological applications. This is because some alloys require very fast cooling to achieve a completely supersaturated solid solution, and in several cases, it is hard to achieve these fast cooling rates in technological applications. Thicker products may also give variations in the material properties across the thickness, due to varying cooling rates. The tendency towards quench-induced precipitation and variation in properties across the thickness are often referred to as quench sensitivity. If cooling takes place at less than a certain critical cooling rate, detrimental quench-induced precipitates grow during cooling. These undesired precipitates are coarse compared to precipitates originating from ageing, and thus lower the amount of solutes available for ageing. Due to the combined effect of coarse quench-induced precipitates and a lowered solute concentration, and thus a reduced fraction of the strength-increasing nano precipitates, several properties are affected and mostly degraded; for instance, the strength improvement, which is often important, is reduced. In many applications, other properties are even more important, such as toughness, fatigue resistance and stress corrosion cracking (SCC). Moreover, quench-induced precipitation also affects the presence and width of precipitation-free zones (after ageing) as well as the kinetics of natural and artificial ageing.

The quench sensitivity of Al alloys has been researched for about 70 years (e.g., [10,11,12,13,14,15,16,17,18,19,20,21,22,23,24,25,26,27,28,29,30]). Within the existing substantial body of work, the definitions of the term “quench sensitivity” differ, and this term generally refers to a reduction in the final properties (e.g., strength; ductility/toughness; corrosion-resistance) in the as-quenched state [21] or after ageing, generally as a result of reduced cooling rates [15,19,27]. The reason for this reduction, for instance in the hardness or yield strength, is the coarse precipitates which grow during cooling [25,31,32]. Although these coarse quench induced precipitates have very little hardening effect in themselves [33], the atomic fraction of alloying elements that is bound in quench-induced precipitates reduces the amount of solutes available for the formation of strengthening precipitates during ageing. The degree of solute reduction obviously depends on the fraction of quench-induced precipitates that is formed. Another aspect relating to quench sensitivity is the loss of properties across the thickness of a thicker product, which is related to a reduced cooling rate in the core of the material [34,35]. However, the definition of quench sensitivity is refined here, and the tendency of an age-hardening alloy to “lose” solute atoms to coarse (some 100 nm to µm sized), undesired quench-induced precipitates is called quench sensitivity. As will be demonstrated below, the quench sensitivity can be characterised by two major attributes: firstly by the minimum cooling rate, which is required to obtain a fully supersaturated solid solution (which after ageing can produce the highest strength), i.e., the so-called upper critical cooling rate (UCCR), and secondly by the slope of property loss as a function of the reducing cooling rate.

With respect to the technological applications, there is another important aspect related to quenching. Rapid quenching can cause severe thermal stresses which may lead to severe distortion [36,37,38,39,40,41,42,43]. This can create important additional costs due to the extra work required for stress-relief or straightening operations. In several cases, the quenching process is therefore confronted with two opposite requirements: cooling as rapidly as needed (to achieve maximum strength), and cooling as slowly as possible (to avoid residual stresses and distortion). Faster quenching may not be detrimental in all cases, since for example some extruded and rolled products are subsequently stretched anyway. Thus, in terms of detrimentally fast quenching, there is a difference between wrought products with simple shapes and cast and other net-shaped products. The alignment of these opposing demands on quenching is therefore a more important issue in the latter type. However, the alloy-specific UCCR typically represents the optimum cooling rate. The maximum achievable quench rates in the heat treatment shop for different types of products (e.g., thick vs. thin gauge) may therefore determine the alloy of choice and thus the strength level achievable.

In order to obtain a comprehensive understanding of the quench sensitivity of age-hardening alloys, the kinetics and nature of quench-induced precipitation must be analysed. Since there were limited options for in situ analysis prior to the major work reviewed here, most of the earlier experimental work was based on ex situ experiments. In 2010 Shuey and Tiryakioglu published a reviewing chapter on “quenching of aluminium alloys” including experimental results on a broad range of alloys as well as theoretical options for their evaluation [44]. Different quenching paths have therefore been frequently used for samples, for instance by using different quenching media [45,46,47,48,49] or by applying Jominy end-quench tests [50,51,52,53]. Subsequent post-quench analysis of certain properties has led to the assessment of the quench sensitivity. Besides continuous cooling, quenching to various isothermal soaking temperatures has also been frequently applied, again in combination with a subsequent property analysis, which allows isothermal time-temperature property diagrams to be derived, for instance [10,18,23,54]. In order to allow evaluation of continuous cooling basing on isothermal experiments, the quench factor analysis method was developed [20] and refined [47,48,52,55,56,57,58].

In contrast to the martensitic hardening of steels, the relevant quench-induced precipitation only amounts to a small percentage of the total atoms. However, this fraction is only transformed in equilibrium conditions. Under technologically relevant cooling conditions, quench-induced precipitation is already suppressed to a wide extent, and this is the reason why very small amounts of precipitation need to be detected under certain relevant conditions. This means that a very sensitive measurement setup is required for the in situ detection of this unwanted precipitation, which covers the entire relevant dynamic range. There are few physical effects that allow for the in situ detection of changes within the solid-solid phase transformation status. Solid-solid phase transformations can rarely be detected directly, although this might be possible to a certain extent using in situ TEM, requiring very high effort. Hence, the in-situ detection and analysis of quench-induced precipitation typically requires the in situ measurement of changes in certain material properties. For this purpose, properties that change with the phase transition of interest are suitable. This may be the electrical conductivity or resistivity [59,60,61,62,63], the associated changes in volume (measured by dilatometry) [64,65], or the crystallographic features (for instance measured by in situ X-ray scattering experiments [66,67,68,69,70,71]). Since the relevant solid-solid phase transformations all show a heat effect (e.g., precipitation = exothermic; dissolution = endothermic), one valuable measurement technique for analysing a solid-solid phase transformation in situ is calorimetry, and particularly differential scanning calorimetry (DSC). The reason for the importance of DSC is that it allows us to measure the enthalpy changes associated with precipitation. Moreover, this precipitation enthalpy is often directly proportional to the atomic fraction of the precipitates formed. However, previous DSC analysis of in situ solid-solid phase transformations is mostly restricted to works that concern heating, and which often carry out only a qualitative evaluation. Additionally, these earlier works commonly lack a sufficient dynamic range, and primarily consider heating rates of about 0.1 to 1 K/s (five to a few tens of K/min) (e.g., [72,73,74,75,76,77]). Until the early 2000s, no DSC analyses involving cooling experiments on quench-induced precipitation in Al alloys were reported; the first works on this topic were published by Kessler et al. [78,79,80] between 2002 and 2007, and Deschamps et al. [31] in 2009. However, due to the use of only a single DSC device in all cases, these works are still restricted to a relatively limited range of cooling rates. There are a few more recent works that have published cooling DSC curves for AlZnMgCu alloys, using only one cooling rate [34,60].

**Figure 3:**
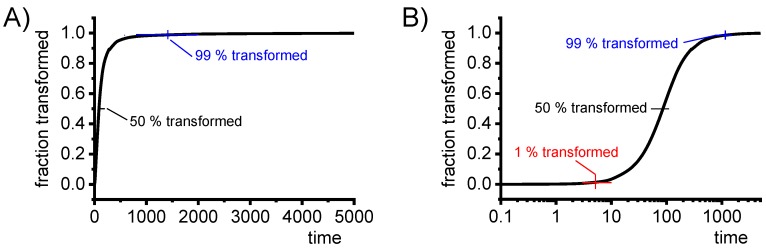
Example of a model for diffusion-controlled precipitation reactions [82]: (**A**) plotted on a linear time scale; (**B**) plotted on a logarithmic time scale.

All of the mentioned heat treatment steps make use of or are directly related to diffusion-controlled solid-solid phase transformations. In particular, in the heating and cooling steps of the heat treatment, solid-solid phase transformations take place under continuously changing temperatures. In practical applications, these dynamic changes in temperature generally depend on the quenching media used and the dimensions of the components and/or the batch (see for instance Fig. 3.64 in Ref. [81], page 613). Since diffusion is strongly dependent on temperature and time, a wide range of relevant heating and cooling rates needs to be assessed. This means that diffusion-controlled processes like precipitation in Al alloys need to be considered on logarithmic time scales. This can be derived from a theoretical description of diffusion, for instance, and is illustrated by an example in Figure 3. Diffusion-controlled precipitation reactions can be described by a Starink-model based on the extended volume *α_ext_* concept [82]. Figure 3 for an isothermal precipitation plots the fraction transformed as a function of time, on a linear time scale (Figure 3A) and on a logarithmic time scale (Figure 3B). It can be seen from Figure 3 that only by using a logarithmic time scale can we analyse this diffusion process in its entirety. Thus, cooling DSC analysis on quench induced precipitation should also cover a cooling rate range spanning several orders of magnitude, and the results should be displayed on logarithmic time scales.

The major progress that has been made relates to an extension of the dynamic range that is accessible for quantitative DSC analysis of solid-solid phase transformations under non-isothermal conditions. That is, conventional in situ DSC on heating and cooling is now possible over a dynamic range of ≈ 3 × 10^−4^ to 3 K/s. When complemented by indirect DSC and indirect DFSC, a total range of 10^−5^ to 10^5^ K/s can be covered. The metrological methods required to achieve this dynamic range have been developed, and currently allow for quantitative evaluation covering the whole range of heating and cooling rates of technological and physical relevance. The consideration of this very broad dynamic width is associated with a large range of associated micro- and nano-structural features, i.e., the dimensions of quench-induced precipitates can range from several tens of µm to just a few nm. Several microstructure observation techniques including optical-, scanning-electron- and transmission-electron-microscopy (as well as other techniques) therefore need to be applied to comprehensively analyse quench-induced precipitates. To complete the analysis, the obtained quantitative DSC enthalpy data are assessed using physically-based models. The models consider the alloy composition and formation of the phases that are most relevant for quench-induced precipitates, as revealed by experiment.

The experimental methods derived can generally be applied to nearly any metallic precipitation-hardening alloy system. Since most of the metrological aspects have been published in great detail in two book chapters [83,84], the experimental aspects are condensed to their key features in this review. The materials science aspects are summarised in this work, involving a substantial number of alloys and thousands of individual experiments. The kinetics and nature of quench-induced precipitation are analysed for a total of 27 alloys, comprising 24 wrought alloys (two AlSi binary alloys, 10 AlMgSi alloys, 10 variants of AlZnMg(Cu) alloys and two AlCu(Mg) alloys) and three AlSiMg cast alloys. The enthalpy changes from quench-induced precipitation are quantified, and the drop in hardness is evaluated in terms quench-induced precipitation. Other properties are not focused on, because the small size of DSC samples does not permit materials tests that require large volumes. In some cases, larger samples with similar temperature/time paths as DSC samples are prepared and mechanically tested.

Based on this work, general conclusions on the nature and kinetics of quench-induced precipitation in Al alloys are drawn.

## 2. Measuring Techniques and Methods for the Analysis of Solid-Solid Phase Transformations

### 2.1. Methods for In Situ Kinetic Analysis of Solid-Solid Phase Transformations in Metals

In metals which allow precipitation hardening, the relevant solid-solid phase transformations typically only amount to a small percentage of the total atomic fraction (excluding Ni-based super-alloys), and thus their detection requires very sensitive measurement setups. As mentioned in the introduction, the in-situ analysis of solid-solid phase transformations typically requires continuous analysis of the property of a particular material that changes with the phase transformation.

In view of this, suitable signals for in situ measurement include:

- changes in the electric conductivity or resistivity;
- changes in the volume;
- changes occurring in the crystal structure and related scattering effects; and
- changes in the enthalpy due to the endothermic nature of dissolution reactions or the exothermic nature of precipitation reactions.

One additional requirement that allows the analysis of quench-induced precipitation, of course, is the need for controlled cooling covering a wide range of cooling rates. This aspect of the metrological implementation might be difficult in several cases. All of the abovementioned methods also have certain advantages and some drawbacks, and these will be briefly reviewed with respect to their capabilities and limitations for the analysis of quench-induced precipitation.

Hong-Ying Li et al. demonstrated that continuous measurements of the electrical resistivity can be applied for the determination of continuous cooling transformation or precipitation diagrams [59,60,85,86,87]. However, it appears that the problem of control over the sample temperature has still not been fully solved, as this group applied several different approaches to achieve continuous cooling, including a self-made temperature control system [59]. They also later quenched samples in different media [60,88], and thereby achieved a considerable range of cooling rates. To the best knowledge of the authors, no commercial device is currently available that allows for the continuous measurement of electrical properties over a wide range of temperatures whilst achieving controlled cooling. In addition, the electrical conductivity is affected by several factors, making interpretations of precipitation difficult. For example, alloying elements in solution reduces the electrical conductivity [89], and precipitates may also have a negative influence on the conductivity [90]. The latter is generally lower compared to elements in solution, although the exact value depends on the size and strain field caused by the precipitates [90,91]. Furthermore, the different alloying elements in solid solution impact the electrical properties by substantially different factors [89]. For these reasons, the measured changes in the electrical resistance or conductivity are not directly related to precipitation or the volume fraction of precipitates, and potential changes in the superimposed influencing factors cannot be directly assigned to precipitation.

Similar problems occur in the dilatometric measurement of quench-induced precipitation. Modern dilatometers have a very high resolution for length changes, which even allow the detection of early cluster formation in age-hardening Al alloys [64,65,92]. It has been shown that dilatometry is also able to detect quench-induced precipitation in Al alloys over a wide range of cooling rates [93,94]. Nevertheless, the changes in the volume of a sample during cluster formation and precipitation are affected by the complex interplay between the volume changes in the matrix crystals and the volume changes due to the precipitated phase. That is, in some cases, precipitation can cause volumetric expansion of the sample, while precipitation of another phase can cause volumetric contraction [64,84,94]. The latter aspect complicates the evaluation of dilatometric measurements of quench-induced precipitation, as different phases that precipitate during cooling can cause opposite changes in length.

Positron lifetime spectroscopy [95,96] can be utilised to investigate the precipitation processes in age-hardening Al alloys [96,97,98,99,100]. However, since the recording of single data points takes at least a few minutes (and up to some hours if the spectra are to be decomposed), this technique would be very slow for in situ cooling experiments [101]. This is probably one reason why no in situ cooling experiments have been published, to the knowledge of the authors.

Another option for the detection of quench-induced precipitates is through the scattering effects of X-rays, i.e., by applying small angle X-ray scattering (SAXS) measurements during cooling of relevant alloys [102,103,104]. In addition to X-ray scattering, small-angle neutron scattering (SANS) can also in principle be used for the detection of precipitation processes [70,105], although the “neutron fluxes are lower and the interaction is also lower, therefore the counting times are usually higher (i.e., hours) in SANS which is a limitation for in situ measurements” [106]. The detection limit in terms of particle size depends on the small angle resolution, and is therefore dependent on the distance between the detector and the sample [106]. These small-angle scattering techniques are typically restricted to the detection of very small precipitates, with dimensions below about 100 nm [71,105,107]. This is a severe limitation for the detection of quench-induced precipitation, as several of the quench-induced particles are much larger. In addition, wide-angle X-ray scattering (WAXS) can be applied for the detection of precipitation processes [108,109]. A specifically adapted Baehr 805 quenching dilatometer has been installed to allow in situ detection of WAXS and SAXS signals within the high-energy materials science beamline “P07” at PETRA III within the *Deutsches Elektronen Synchrotron* (DESY). This setup allows the in situ detection of phase formation during cooling of metallic alloys [110]. The applied high energy of this beamline results in a sufficient time resolution for in situ experiments, and the capability of this setup for in situ analysis of quench-induced precipitation in AlZnMgCu alloys has been demonstrated very recently [111]. Using the abovementioned scattering techniques, information on the presence of certain crystal structures and therefore phases can be gained (WAXS), and conclusions on the particle size and volume fractions can be drawn (SAXS, SANS). For details on these techniques and their theory, the reader is referred e.g., to Ref. [112].

Since precipitation is an exothermic process, and dissolution an endothermic one, they can be detected by thermal analysis. Cavazos and Colas used direct thermal analysis of Jominy-like end-quench samples, i.e., they evaluated the changes in slope of the measured temperature development at different positions in the sample [25]. A more precise analysis of the thermal effects of solid-solid phase transformations is possible using calorimetry. DSC, in particular, allows for the detection of quench-induced precipitation in age-hardening metals [31,78,79,80]. Amongst the potential methods for the in situ detection of quench-induced precipitation in aluminium alloys, DSC is the most effective approach, as it has significant advantages. These in brief are:

- DSC allows for controlled linear heating and cooling by providing the largest accessible dynamic scanning rate range, which now covers the whole cooling rate range of technological and physical relevance. By using a combination of different DSC devices, rates of about 10^−5^ to 10^5^ K/s [83,84] can be achieved.
- DSC samples are typically in the mm range, which in most cases allows further analysis after the DSC experiment, for instance for hardness testing or microstructural investigations [83,84,113].
- In addition to linear cooling, nonlinear Newtonian heating and cooling can also be investigated using DSC [114].
- With regard to the abovementioned detection limits, DSC offers the highest sensitivity of all techniques over the whole dynamic range. In particular, precipitation of any precipitate size and very small volume fractions of precipitation can be detected by DSC, and even cluster formation can be recorded [99,115].
- If properly conducted, DSC allows for quantitative evaluation of the enthalpy changes related to precipitation. Since these enthalpy changes are directly proportional to the atomic fraction transformed, analysis of the volume fraction of precipitates is possible [116,117,118,119].

However, DSC also has certain disadvantages and limitations. In particular, DSC detects the heat effects of all occurring reactions in sum. Since different reactions occur simultaneously in several cases, it can be hard to distinguish the different parts of the signal that belong to specific reactions. This makes the interpretation of DSC heating experiments particularly complicated, as opposite heat effects from endothermic dissolution and exothermic precipitation can occur during heating [120]. Since only precipitation will take place during cooling, DSC still is the most promising method for comprehensive analysis of the kinetic behaviour of quench-induced precipitation.

### 2.2. Methodology and Systematics of State-of-the-Art Kinetic DSC Analysis

#### 2.2.1. Basic Concepts of DSC Measurements

**Figure 4:**
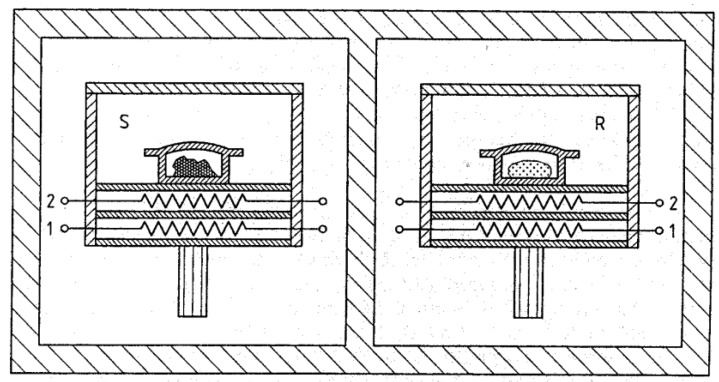
Construction scheme of a power-compensated scanning calorimeter (PerkinElmer Instruments). S: Sample furnace with sample in crucible; R: reference-furnace (analogous to sample-furnace); 1: heating wire; 2 resistance thermometer. Both sensors are separated from each other and located in surroundings at constant temperature (cold block) [113].

DSC allows us to measure heat release or consumption, or in other words the amounts of enthalpy that are exchanged by samples, for instance due to phase transitions. In order to enhance the sensitivity compared to a classical calorimeter and to allow non-isothermal operation in a differential calorimeter, the signals of two calorimetric sensors are compared [113]. Operation under non-isothermal conditions is possible, as both sensors are located symmetrically in surroundings at the same temperature (see the schematic example of power-compensated DSC sensors in Figure 4). A differential scanning calorimeter is generally operated in scanning mode rather than under isothermal conditions, i.e., the temperature typically changes at a constant heating or cooling rate. DSC uses two symmetric (ideally identical) sensors, one of which measures the sample to be investigated, while the other measures an inert reference sample. There are two different types of signals that can be measured: either the temperature difference between the two sensors, or the difference in heating power. In the first case, the device is called a heat flux differential scanning calorimeter, and both sensors are located in one furnace, which is used for the general temperature-time program providing the same surrounding temperature for both sensors. In the second case, both sensors are located separately, in surroundings of constant temperature (a cooled block), and both are equipped with a micro furnace that incorporates a temperature sensor, i.e., the sensor is both a furnace and a sensor at the same time. This version of DSC is called power-compensated DSC (see Figure 4). Nevertheless, both types of DSC devices can be used in a very similar way, and the setup of the experiments and the data evaluation are very alike, as in both cases the (electronic) signal is converted to the value of heat flow by calibration. For more details on specific setups and the peculiarities of the different types and operation modes of DSC devices, the reader is referred to Ref. [113].

**Figure 5:**
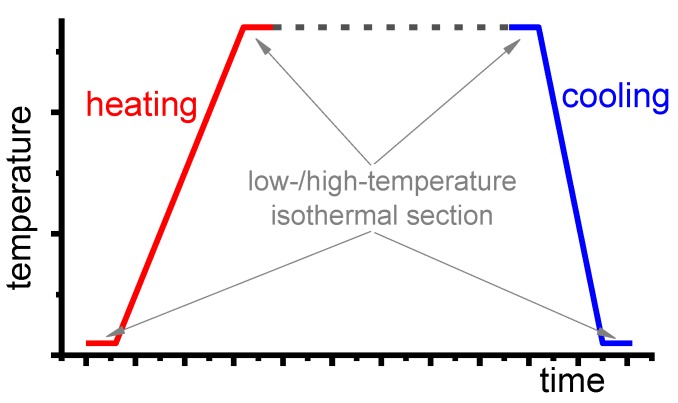
Basic time-temperature profile of DSC experiments.

In this work, each temperature-time profile of a DSC measurement starts and ends with an isothermal section (see Figure 5). This is needed for heat capacity calculation employing two or three curve methods (for details see [112]).

In general, an accurate DSC analysis of the sample requires at least two measurements: a measurement of a sample versus a reference, and a baseline measurement (inert reference samples on both sensors). The heat flow signal of the baseline measurement is subtracted from the heat flow signal of the sample measurement, and the resulting heat flow signal is corrected for small device-specific asymmetries which, although minimised in the design, can still influence the accuracy when low-heat-flow, high-accuracy measurements are required. Examples of such small asymmetries include slight differences in sensor positions and differences in the sensors themselves.

#### 2.2.2. Key Features for In Situ Quantification of Enthalpy Changes of Solid-Solid Phase Transformation as a Function of the Scanning Rate or Time

##### Excess Specific Heat Capacity

**Figure 6:**
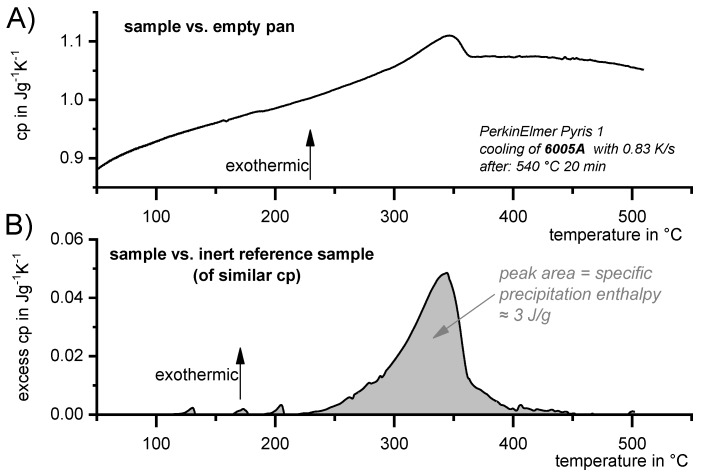
Comparison of two basic measurement set-ups: (**A**) a sample versus an empty reference sensor, acquiring the specific heat capacity of the sample; (**B**) a sample versus an inert reference sample of similar heat capacity, acquiring the specific excess heat capacity of the sample compared to the reference.

To allow for quantitative analysis of solid-solid phase transitions, very sensitive measurement setups are required. To obtain the best possible DSC sensitivity for this particular purpose, and to allow a purposeful comparison of different heating rates and sample masses, the experimental setup needs to allow for conversion of the measured signals to the specific excess heat capacity of the sample. When studying precipitation reactions, the contribution of the heat capacity can be subtracted during the measurement by using inert reference samples [121]. In the following, the apparent heat capacity due to precipitation (latent heat) in addition to the heat capacity of the matrix is called excess heat capacity. Figure 6 clarifies the difference between a conventional measurement of sample heat capacity (Figure 6A) and a measurement of the specific excess heat capacity (Figure 6B). The heat capacity (measurement setup sample versus an empty reference sensor) can be considered as the sum of the heat capacity that increases with temperature and the apparent heat capacity caused by the latent heat effects due to precipitation. In the case of an alloy that shows any kind of phase transformation, the measured apparent heat capacity is a sum of the heat capacity and the effects due to heat or enthalpy release/consumption by the occurring phase transition. The latter, of course, has a dependence on temperature and time.

The specific excess heat capacity is obtained here by measuring an alloyed sample versus a reference sample with similar heat capacity. The specific excess heat capacity is then defined to be the difference between the specific heat capacity of the alloyed sample and that of an inert reference sample. In an ideal situation, the reference and the alloy sample have highly similar absolute heat capacities over the entire temperature range of interest. Another requirement for the reference sample is that it must be inert with respect to thermal reactions within the temperature range of interest. It may be difficult to find appropriate reference material, but in the case of Al alloys, pure Al has proven to be very well suited [120,121]. Pure Al reference samples are preferably used in a pre-oxidised condition, i.e., heated once before the actual measurement; however, there are various strategies for obtaining a suitable reference sample [122], and changes in the sample mass to adjust the total heat capacities of the sample and reference sample may further improve the signal quality [122,123].

To acquire the excess specific heat capacity (*excess c_p_*), the alloyed sample is placed in the sample sensor, while the reference sample is placed in the reference sensor. Since both sample and reference have very similar absolute heat capacities, this setup retains the symmetry within the differential scanning calorimeter and its entire measurement setup. This supports the basic idea of DSC, which is a steady comparison of two equal measuring systems. In an ideal system, the only remaining differences—and therefore the only remaining thermal asymmetries—are thermal effects related to reactions within the sample. The resulting signal can therefore be directly and solely related to the phase transformation of interest.

As described above, in the first instance the measured signal is a heat flow signal. The DSC heat flow signal depends on the amount of sample used (expressed as a mass or in moles) and the scanning rate applied. To allow for a quantitative comparison of measurements with different scanning rates and sample masses, the heat flow signal $\dot{Q}$ must be normalised by the sample mass (or the number of atoms) and the scanning rate β, resulting in the unit of specific heat capacity (per g or per mole):

(1) $$excess\;c_{p}=\;\frac{{\dot{Q}}_{sample\;vs\;reference}-{\dot{Q}}_{baseline\;\left(reference\;vs\;reference\right)}}{sample\;mass\;\times scanning\;rate}\;\left[excess\;c_{p}\right]=1\;\frac{mW-mW}{mg\;\times \;\frac{K}{s}}=1\frac{Ws}{g\times K}=1\;\frac{J}{gK}$$

The *excess c_p_* thus represents only the changes in specific heat capacity that are caused by a reaction inside the sample. Integration of the *excess c_p_* peak versus temperature (bounded by the zero level) determines the specific precipitation heat, or more generally, the enthalpy changes caused by the occurring phase transformation.

##### Zero-Level Accuracy

**Figure 7:**
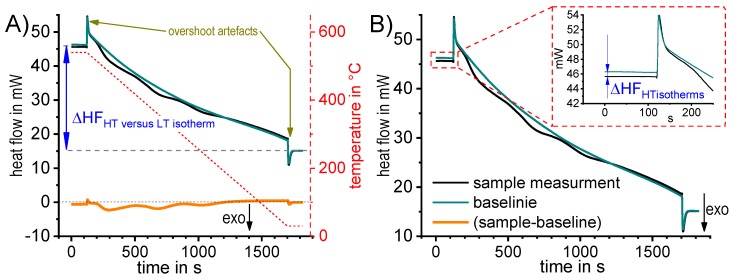
Quality attributes of DSC raw data. A & B show the same raw data, measured by cooling an AlCuMg alloy with 0.3 K/s in a PerkinElrmer Pyris 1 DSC. (**A**) Overshoot artefacts and the quality attribute of the heat flow difference between the high- and low-temperature isotherm, are highlighted; (**B**) the quality attribute of heat flow difference between sample and baseline measurements in the isothermal section at high-temperatures is highlighted.

In terms of the intended evaluation of solid-solid phase transitions, the zero level of the considered DSC measurement is of outstanding importance, particularly when the enthalpy changes are to be determined quantitatively. The measured signal of excess *c*_p_ can still be curved, and may particularly deviate from zero in temperature regions in which no reactions are occurring (the excess c_p_ should be zero here). This zero-level curvature can have multiple reasons. To ensure a reliable and straight signal close to zero level, only adequately calibrated calorimeters should be used (for calibration details, see [113,124]). It is also reasonable to check each DSC run and its corresponding baseline measurement using the following quality attributes: (i) the heat flow difference between the high- and low-temperature isothermal section (Figure 7A); and (ii) the heat flow difference between the heat flow values of the sample and baseline measurements of the high-temperature isothermal section (Figure 7B). The acceptable values of the heat flow difference depend on both the specific device and their continuity with time; for instance, the heat flow difference between the isothermal sections at high and low temperatures depends mostly on the symmetry and adjustment of the device. More important than the absolute values of these differences is the reproducibility. An unfavourable value may be acceptable as long as it is constant with time [83], but for highest accuracy, the values of heat flow differences should be small, i.e., in case (i) within the range of few tenths of mW, and in case (ii) within the range of a few mW for the PerkinElmer power-compensated differential scanning calorimeter.

To obtain good zero-level accuracy, it is important to ensure that symmetry is maintained within the experimental setup. This includes sample and reference heat capacities [122,123], crucible or pan masses/heat capacities, and of course sample positioning and potentially sensor cover placement [125].

**Figure 8:**
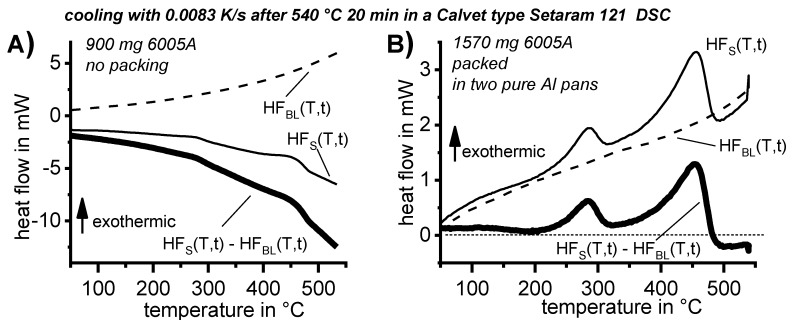
Effects of inadequate or adequate sample packing in a CALVET-type heat flux differential scanning calorimeter under otherwise very similar experimental conditions: (**A**) heat flow of sample (HF_S_) and baseline (HF_BL_) measurements without sample packing showing substantially different heat flow in regions without phase transformations; (**B**) if the samples are packed properly HF_S_ and HF_BL_ are very similar in reaction-free temperature regions, i.e., subtracting HF_BL_ from HF_S_ results in a nearly straight zero-level.

In any case, the sample must be packed properly, for instance using high-purity aluminium pans. If this aspect is disregarded, an incorrect measurement might result (see Figure 8). The reason for this is that the colour of the sample surface, which influences the heat exchange by radiation between sample and furnace, needs to be kept constant [121]. A purge with inert gas is also required to prevent oxidation and keep heat transfer conditions constant. Nitrogen gas is therefore preferable to helium, as nitrogen purging results in the best baseline stability of the DSC instrument. Helium may be suitable for increasing the achievable cooling rates [78].

For each individual sample measurement, a corresponding baseline measurement needs to be performed [75,113,121], and this measurement should be made promptly. This is particularly true for power-compensated devices, as their baseline behaviour drifts more strongly with time [126] than that of heat flow calorimeters.

In terms of statistical validation, a sufficient number of experiments is required, and the exact number depends on the sample and the considered effect. Of course, the number of experiments is always a compromise between statistical validation and time effort, and this especially holds for slow, long-lasting measurements. For these, two to four experiments and one or two baseline runs are typically done, while for the faster experiments at least six samples and three baselines are generally measured.

Even if all of the abovementioned aspects are ensured, the zero level of the resulting specific excess heat capacity may still show a slight curvature, and this is slightly different for each experiment [75]. This may be caused by slight differences in the sample position for each individual measurement, for instance [125]. Thus, the raw DSC data must undergo a sophisticated data treatment prior to evaluation, including baseline subtraction in each case, and possibly also the elimination of the remaining zero-level curvature [120] or a mean value curve calculation [122,123,127]. If efforts are made to ensure the best zero level quality, a result that is straight and close to zero can be achieved. In heating experiments, this allows us to judge whether exo- or endothermic reactions predominate at a certain temperature or time [75,120]. In addition, the zero level can act as an integration reference for enthalpy calculations [75,120,121].

##### Large Dynamic Range

A large dynamic range (of time or heating/cooling rates) allows us to comprehensively analyse the kinetic development of single reactions. Any relevant solid-solid phase transition is related to diffusion, and as described above, diffusion is dependent on logarithmic time scales, implying the need for a large dynamic range. However, this need for a large dynamic range can be considered from two perspectives: (i) the specific physical and alloy requirements and (ii) metrological aspects.

##### Specific Physical Requirements for the Alloy over the Considered Dynamic Range

To enable a full physical understanding of solid-solid phase transformations, the required high dynamic range is bounded on the one hand by very slow transformations close to equilibrium conditions, and on the other hand by relatively fast cooling beyond the critical cooling rates of the relevant transitions. For instance, for aluminium alloys, the adjustment of maximum strength values requires the complete suppression of any precipitation reaction upon cooling from solution annealing. The slowest cooling rate that just retains all alloying elements in solid solution is the UCCR [128]. For highly alloyed Al-Zn-Mg-Cu wrought alloys or Al-Si-Mg cast alloys, the UCCR can easily reach several hundreds of K/s [129,130,131]. The lower critical cooling rate defines the fastest cooling rate at which all the alloying elements (compared to the equilibrium solubility) precipitate during cooling from solution annealing, yielding enthalpy changes on a saturation level. For an Al-0.72Si alloy, this lower critical cooling rate is about 3 × 10^−5^ K/s [117,132]. To identify both critical cooling rates, they must be experimentally exceeded by at least one order of magnitude in terms of both slower and faster cooling, which requires a dynamic range of about 10^−5^ K/s to 10^3^ K/s. This wide dynamic range is now available, even for cooling, in scanning calorimetry using multiple devices.

**Figure 9:**
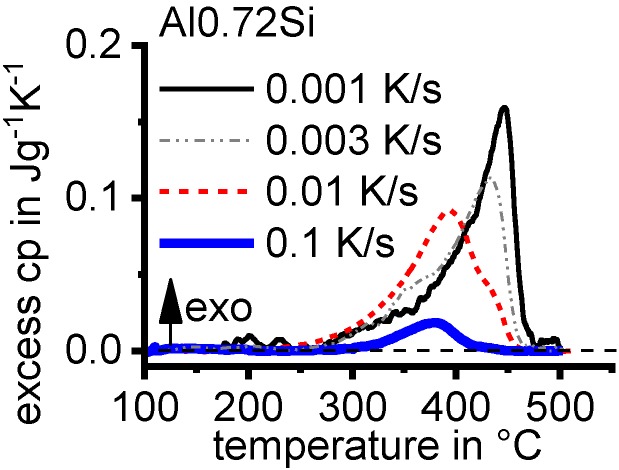
DSC cooling curves of a pure binary Al-Si alloy covering a wide range of cooling rates. It can be seen that exothermic precipitation reactions at higher temperatures are only detected at slower cooling rates. Original data from [117].

Aside from the determination of the critical cooling rates, a further aspect can only be understood if almost the full range of cooling rates is considered. As discussed later, it has been shown that for any age-hardening alloy, quench-induced precipitation during slow cooling predominantly occurs at alloy-specific high temperatures (compare e.g., [117,121,133]). At faster rates, the high-temperature reactions will be suppressed to a certain extent, and maybe even suppressed completely, and quench-induced precipitation will then probably take place via a medium- or low-temperature reaction [119,133]. An example is shown in Figure 9 for the pure binary alloy Al0.72Si. At a “high” cooling rate of 0.1 K/s (which may be a relatively slow cooling rate for higher concentrated alloys), quench-induced precipitation only occurs in a “low-temperature reaction” (≈400–300 °C). In contrast, during slow cooling at 0.001 K/s, the vast majority of quench-induced precipitation occurs at high temperatures (≈470–350 °C). At medium rates, both types of reactions overlap.

Thus, if only the faster rates are considered in the analysis of quench-induced precipitation, the high-temperature reactions may be disregarded [121]. By inversion of the above argument, it can be seen that all relevant precipitation reactions can be detected only if the full range of physically relevant cooling rates is analysed.

##### Metrological Aspects of a Large Dynamic Range

**Figure 10:**
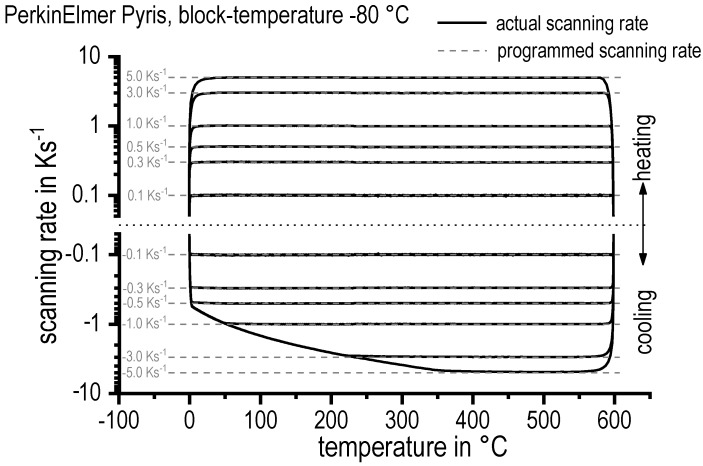
Typical performance diagram of a PerkinElmer Pyris diamond DSC.

Obtaining a large dynamic range is a much bigger challenge in cooling experiments than in heating experiments. Each DSC device has its own spectrum of heating/cooling rates, which also depends on the particular setup of the device (see Ref. [83] for more details). The scanning rate spectrum is typically analysed by measuring a performance diagram (see the example in Figure 10). It can be seen that during heating, a scanning rate of 5 K/s is reached after a short period, and can be controlled over nearly the entire temperature range, which was 0 to 600 °C in this case. However, during cooling, control over the scanning rate is lost at a certain temperature, and this takes place earlier at increasing cooling rates. The loss of cooling control is accompanied by the loss of an evaluable DSC signal, which is typically more severe for power-compensated calorimeters. In the example given in Figure 10, the critical temperature during cooling at 5 K/s is about 350 °C. This is clearly a limiting factor, since relevant reactions might occur at lower temperatures, and these cannot be therefore analysed with this particular DSC device at this cooling rate.

**Figure 11:**
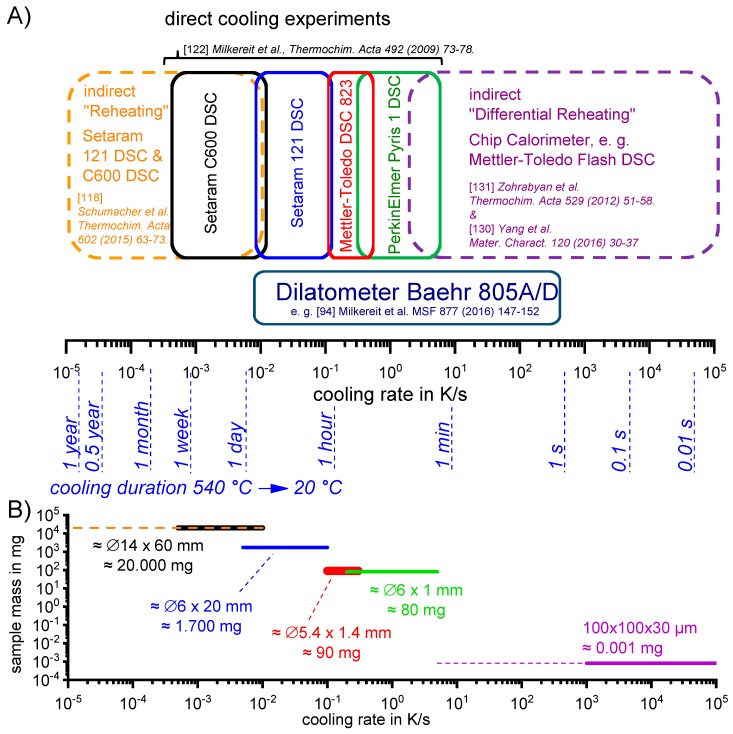
(**A**) Scanning rate ranges of different DSC devices and the quenching dilatometer; (**B**) related sample dimensions and masses (in the case of Al samples). In order to make the broad dynamic range easier knowable the cooling rate axis is complemented by the durations of cooling, which are needed to cover the temperature range of 540 to 20 °C (typical for the age-hardening of Al alloys).

To obtain a dynamic range that covers cooling rates of several orders of magnitude and also allows us to analyse the full temperature range, it is essential to combine several types of DSC device [83]. In this work, a total of five different DSC devices were used: two relatively slow CALVET-type heat-flux differential scanning calorimeters, a disc-sensor type heat-flux differential scanning calorimeter, a power-compensated differential scanning calorimeter and a chip-sensor-based differential fast scanning calorimeter (DFSC, [134]). These devices and their ranges of scanning rates can be seen in Figure 11A. As is obvious from Figure 11B, the large variation in cooling rates also causes a strong variation in the sample mass. Large sample masses are crucial for slow scans, since the heat flow signal is proportional to the scanning rate and sample mass. This means that if the scanning rate is very small, the signal can only become strong enough for detection if the sample mass is large. On the other side of the spectrum, small sample masses are essential to allow for fast cooling [134]. The cooling rate range can be extended in either direction (slower and faster cooling) by indirect measurements [117,129]. The basic concept of these extending experiments is the same in either direction, and will be explained in Section 3.1.3.

Another aspect is that in general, if cooling becomes faster, precipitation is increasingly suppressed, and the amount of heat released decreases with increasing cooling rate when approaching the UCCR. This is because precipitation reactions are diffusion-controlled, and diffusion is increasingly suppressed if the time available shortens. One very important aim of these studies from a technological point of view is the detection of the UCCR at which precipitation during cooling is completely suppressed, i.e., at which all alloying elements dissolved during solution annealing remain in a supersaturated solid solution. The UCCR is very relevant in technological applications, and it is shown below that the maximum age-hardening potential can be exploited only if the alloy is cooled at the UCCR or faster. However, faster cooling may result in undesired residual stresses and distortion of age-hardened aluminium components [38,41,42,43,104,135,136,137]. Although this is of lower importance for extrusions or rolled products, which are stretched after quenching, residual stresses and distortion are very important for complex cross-section extrusions and net-shaped cast and forged products.

In the DSC analysis, the measure of the fraction of precipitates is the specific precipitation heat (or enthalpy) released by the precipitation. On reaching the UCCR, the enthalpy change by precipitation is obviously zero. This leads to a metrological problem: in a range of cooling rates close to the UCCR, we need to determine whether no precipitation heat is detectable in the measured curves. Thus, the challenge is to decide when a precipitation signal is above the device-specific noise level.

An objective criterion is therefore needed. The detection limit was defined in Ref. [121] in the following way:

- The reaction is detectable in at least three repeated experiments;
- The reaction is also detectable at the next slower cooling rate;
- The specific precipitation heat is at least 0.1 J/g; and
- Peak temperatures are in the same region as for the next slower rate.

The detection limit of 0.1 J/g for instance in the Al-Mg-Si system corresponds to a Mg_2_Si precipitation volume fraction of about 0.01% [116]. For comparison, the maximum precipitation heat measured by DSC of 6005A alloys amounts to 12 J/g, and the related β-Mg_2_Si precipitate volume fraction amounts to 1.15%.

For correct curve interpretation, evaluation must be done from the slowest to faster cooling. Other features of DSC cooling experiments can be found in Refs. [83,84].

**Figure 12:**
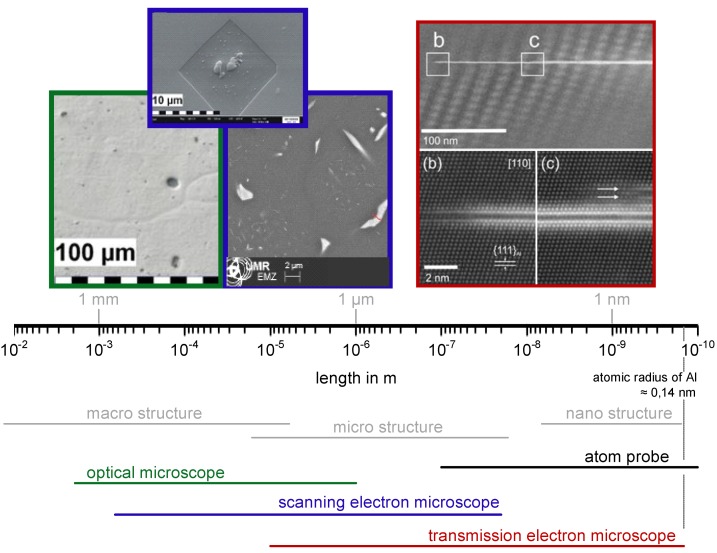
Dimensions of quench-induced precipitation illustrated by micrographs from optical, scanning electron and transmission electron microscopy from an AlMgSi and an AlZnMgCu alloy.

The required wide range of cooling rates is accompanied by another metrological challenge, in that the dimensions of the quench-induced precipitates vary substantially within the cooling rate range. This aspect is illustrated in Figure 12. It can be seen that the dimensions of quench-induced precipitates vary between several tens of µm down to thicknesses of a few atomic layers (≈nm), i.e., the dimensions vary by a factor of >10,000. Comprehensive imaging of quench-induced precipitates over the entire relevant dynamic range (of cooling rates and duration) requires the application of three different types of microscopy: optical microscopy (OM), scanning electron microscopy (SEM) and transmission electron microscopy (TEM). Of course, the application of additional analytical methods can provide further detailed information on quench-induced precipitation, and these will be briefly introduced in Section 2.4.

### 2.3. Construction Scheme for Continuous Cooling Precipitation Diagrams

A substantial compression of the information generated for quench-induced precipitation is plotted in continuous cooling precipitation (CCP) diagrams, which correspond to the well-established continuous cooling transformation (CCT) diagrams for steels. CCP diagrams allow us to choose suitable heat treatment parameters for a certain alloy in the heat treatment shop, and thus allow for the practical application of the findings on the quench sensitivity of Al alloys. Furthermore, they are important input data for heat treatment simulations.

**Figure 13:**
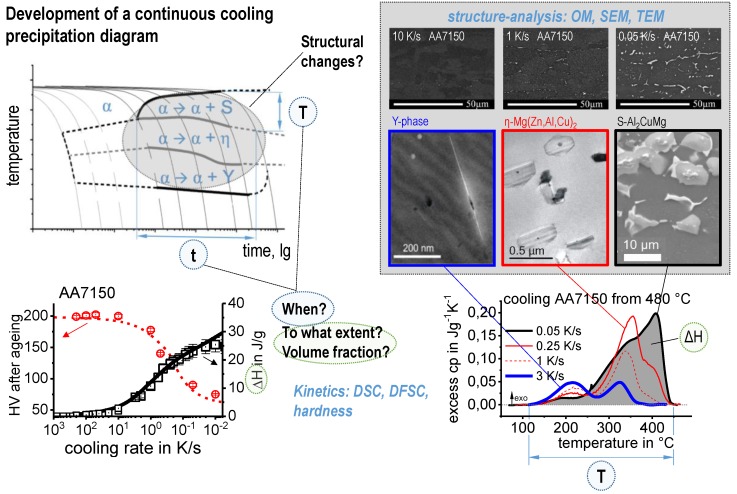
Scheme of the different analyses required for construction of a complete continuous cooling precipitation diagram, illustrated here for alloy 7150.

Since a CCP diagram involves the kinetic behaviour of precipitation during cooling from solution treatment, the time-temperature profiles of the varied linear (i.e., constant) cooling rates form the basis of the diagram. These are plotted on a temperature-time diagram with logarithmic scaling of the time axis. To allow construction of a CCP diagram for a specific alloy, three distinct aspects of quench-induced precipitation need to be analysed for each specific alloy using different experimental methods, as schematically illustrated in Figure 13.

In the first step, the temperature and times at which a distinct precipitation reaction starts and finishes need to be determined. This can be measured by DSC: the onset or end temperature of the reaction can be read directly from the DSC curves, and the associated time values are derived from the cooling rates applied. The obtained values for the onset or end temperatures and times of the reaction are inserted into the related cooling rates. In this way, the single values for any relevant cooling rate can be graphically connected by splines, to illustrate the regions of the specific precipitation reactions. In the second step, the intensity of the distinct precipitation reactions (or at least the total overall precipitation) at this cooling rate needs to be determined. This relates to the amount of the fraction transformed, i.e., the atomic or volume fraction of precipitation. Although this question might be hard to answer in detail, the specific precipitation enthalpy measured by DSC at least gives a value that is directly proportional to the fractions transformed. In the third and final step, the nature of the micro- and nano-structural changes related to the different DSC peaks needs to be determined, i.e., we need to determine which phases are precipitating. This involves several additional scientific aspects, for instance the chemical composition, crystal structure, particle morphology, active nucleation mechanism, particle size distributions and particle densities depending on the cooling rate. In some cases, several of these aspects cannot be revealed with high accuracy; however, any information generated in this respect helps to complete the CCP diagrams.

### 2.4. Methods and Systematics for Complementary Micro- and Nano-Structure Analysis

As described above, a comprehensive understanding of the microstructural changes in the alloys caused by cooling is crucial. As shown in Figure 12, the dimensions of quench-induced precipitates vary substantially and cover about five orders of magnitude. Thus, the use of a range of microscopes is required for imaging of the relevant precipitates. Coarse quench-induced precipitates originating from high temperatures and slow cooling rates can be imaged by OM [112,116,138]. OM images also allow us to evaluate the volume fractions of coarse quench-induced precipitation [116,118,119,138,139]. SEM (e.g., [140]) allows imaging of coarser quench-induced precipitates. Imaging is typically done with either secondary electrons (SEs) or backscattered electrons (BSEs), the latter of which give better contrast for different atom masses. Analytical techniques integrated into SEM allow further valuable information to be acquired; for example, energy-dispersive X-ray spectroscopy (EDS) enables the determination of the chemical composition of precipitates [112,138], while electron backscatter diffraction (EBSD) can reveal the crystal structure of coarse precipitates [138]. EDS always involves a compromise between spatial resolution and element-specific stimulation. A sufficient acceleration voltage is required for the EDS detection of alloying elements with heavier atomic masses; for example, the EDS detection of Zn typically requires an acceleration voltage of 25 kV [112]. However, this enlarges the interaction volume, and may make it larger than the particle of interest. Thus, in most cases, it is rarely possible to obtain EDS signals solely from the precipitate, and part of the stimulated and measured X-rays generally originates from the matrix [138].

The presence of crystal structures with volume fractions of above ≈1 % and large precipitates can be detected by common X-ray diffraction (XRD) [112,138]. Hence, this technique is again limited to relatively slow cooling rates, which produce corresponding volume fractions and particle dimensions of the phase of interest. In addition, the wide and small-angle X-ray scattering techniques briefly discussed above can generate information on quench-induced precipitation [102,103,104,111].

Imaging of precipitates of dimensions below about 1 µm is commonly done by TEM (e.g., [141]) [112,118,119,128,142,143]. In this work, bright-field TEM images and high angle annular dark field (HAADF) images from (scanning) TEM are used. In TEM, additional analysis with EDS allows conclusions to be drawn on the elemental composition of certain relevant particles, down to precipitate structures composed of just a few layers of atoms [143]. The chemical analysis of the latter can potentially be supplemented or even improved by the application of electron energy loss spectroscopy (EELS) [143]. When using TEM, information on local crystal structures can be generated by selected area electron diffraction (SAED) or nanobeam diffraction (NBD).

Most of the structural information considered in this review was obtained through international collaborative work.

**Figure 14:**
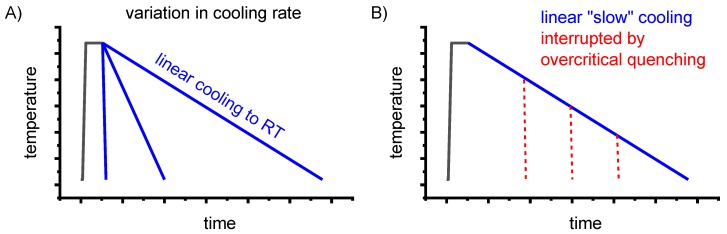
Schematic presentation of heat treatments applied to alloys in this work (introduced in [128]): (**A**) variation in cooling rate; (**B**) interrupted cooling.

To improve the meaningfulness of the structure analysis, it is helpful to investigate samples using systematically varied heat treatments. Two major heat treatment routes were therefore examined (see Figure 14). Firstly, the development of quench-induced precipitation during linear cooling with varying rates down to room temperature is analysed. The formation of quench-induced precipitates at a certain cooling rate is also analysed via cooling to a specific temperature followed by rapid overcritical quenching, which allows us to match certain quench-induced phases to specific peaks found in the DSC curves.

### 2.5. Analysis of Resulting Mechanical Properties

The small dimensions of the DSC samples only allow hardness testing to be done directly on the DSC sample. Hardness testing (commonly Vickers hardness HV1 or HV5) was done regularly after cooling at various rates and subsequent (mostly artificial) ageing.

However, hardness is only loosely related to strength. Thus, in some cases, additional compression or tensile tests were performed on samples cooled at controlled rates to certain temperatures. Details of these tests can be found in Refs. [37,117,132]. In general, thermo-mechanical-analysis (TMA) is performed with a quenching and deformation dilatometer to obtain mechanical properties from tensile [144] and compression tests [36,37,132,145].

### 2.6. General Illustration of Results and Reading Guidelines for CCP Diagrams

In the following sections, a large number of similar plots are presented. In particular, three types of plots occur several times. To ensure a full understanding, their general layout is explained here.

DSC curves are generally presented in terms of the specific excess heat capacity as a function of temperature. The associated zero level of the DSC curve is plotted as a dashed straight line. In the case of DSC heating experiments, the zero level allows us to assess whether exo- or endothermic reactions predominate, while endothermic reactions are plotted in the upward direction. For cooling DSC curves, only precipitation reactions can occur. For cooling experiments, by definition, exothermic precipitation reactions are plotted in the upward direction. In plots with multiple cooling DSC curves at various scanning rates, the zero levels are in most cases shifted by a fixed offset, with the DSC curve with the slowest scanning rate on top. The specific solution treatment applied prior to the DSC cooling experiments is stated in plots of this type.

In another frequently used plot, the specific precipitation enthalpy is plotted as a function of the cooling rate. A third type plots the hardness after cooling at various rates and subsequent (mostly artificial) ageing. In several cases, the latter two diagrams are combined into one plot with two ordinate axes. To allow interpretation and comparison with the timescale of CCP diagrams, the cooling rate axis is scaled in descending logarithmic order, i.e., proportional to the duration of cooling.

As the most compressed illustration of information on quench-induced precipitation in Al alloys, CCP diagrams describe the precipitation behaviour of aluminium alloys during continuous cooling from solution annealing, as a function of temperature and time. Since a very broad time (cooling rate) range is physically and technologically relevant, the time axis is scaled logarithmically. We define the time axis as ranging from 0.1 s to 1,000,000 s in each case, while the plotted temperature range is fixed at 550 °C to 0 °C. This allows us to cover the relevant time and temperature scales for most alloys.

CCP diagrams can only be read along the continuous cooling curves, and isothermal readings are not possible from these diagrams. Moreover, CCP diagrams are only valid for the particular alloy composition investigated and the specific solution treatment applied, which both have a great impact on the quench sensitivity of the alloy. The solution treatment and the mass fractions of alloying elements are therefore given at the top of each diagram. This also holds for the initial microstructure, i.e., the grain size or secondary dendrite arm or cell spacing. The reader should be aware that different batches with similar chemical composition may therefore still behave differently.

The start and finish temperatures of precipitation, depending on quenching rate, are shown by bold lines. In almost all cases, several precipitation reactions occur sequentially during cooling and there is often some overlap. The onset of precipitation in the first reaction and the end of precipitation in the last reaction can be determined exactly, and are shown in continuous black lines. Overlapping reactions are hard to separate by DSC, and intermediate start and finish precipitation temperatures have been estimated using the procedure outlined in Ref. [114,118] and are shown in continuous grey lines. In some cases, precipitation start and finish temperatures have been extrapolated for readability by dashed black lines.

To provide information about precipitation intensity, the total cooling rate specific precipitation enthalpy ∆h_total_ is plotted below certain cooling paths in rectangles. The specific precipitation enthalpy is proportional to the volume fraction of precipitates formed during cooling. The Vickers hardness HV1 (HV5 for some 7xxx alloys) after cooling and additional ageing is shown by ovals. The precipitation enthalpy is high for slow cooling, whereas hardness after ageing is high for rapid quenching. Strong precipitation during cooling causes a loss of availability of alloying elements for subsequent ageing.

The slowest cooling rate at which a certain precipitation reaction is completely suppressed during cooling is defined as the UCCR. These values are provided in the lower left-hand corner of the diagrams. In cases where (in situ) DSC measurements were not possible up to cooling rates above the UCCR, the latter is estimated based on the hardness results.

**Figure 15:**
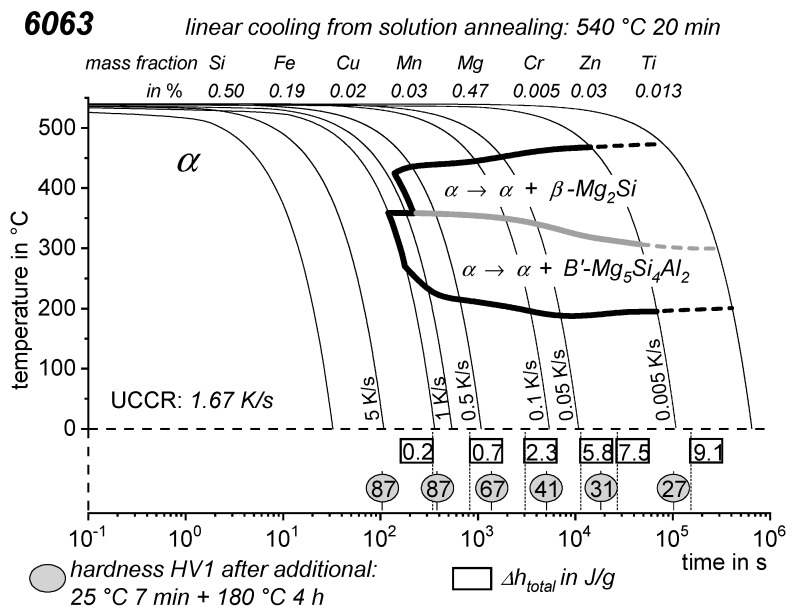
Continuous cooling precipitation diagram of 6063, as published in Ref. [146].

An example of a CCP diagram in the format described above is shown in Figure 15. In Ref. [146], we published CCP diagrams in this format for 11 Al wrought alloys and one Al cast alloy. A range of additional CCP diagrams is found in the Appendix A of this publication.

## 3. Solid-Solid Phase Transformations in Al Alloys over a Wide Dynamic Range

This chapter provides assessments of the kinetics of solid-solid phase transformation, and particularly the kinetics of quench-induced precipitation for two AlSi binary wrought alloys, two high purity AlMgSi ternary wrought alloys, and a range of 20 wrought alloys from the heat treatable alloy systems AlMgSi (eight variants), AlZnMg(Cu) (10 variants) and AlCuMg (two variants). Some of the aforementioned alloys are laboratory alloys, and some were produced commercially; however, all contain amounts of trace elements and impurities typical of commercial production. Three AlSiMg cast alloys are also considered. Table A1 in the Appendix A gives alloy compositions and some details of their original processing.

### 3.1. Heating to Solution Treatment and Isothermal Soaking

#### 3.1.1. Capabilities and Limitations of DSC Heating Curve Analysis and Interpretation

The vast majority of published DSC work on Al alloys involves heating experiments. This is likely to be due to the fact that DSC was originally intended for use during heating, and DSC heating experiments are easier in terms of scanning rate control (see Figure 10). However, the interpretation of heating DSC curves for age-hardening Al alloys is complicated by severely overlapping endo- and exothermic reactions i.e., dissolution and precipitation reactions [120]. Unfortunately, the superposition of different reactions is rarely verifiable and thus is too often disregarded. As will be shown later, the consideration of DSC heating curves over a wide range of heating rates can help to identify superposition issues. In DSC heating experiments, it is reasonable to choose the maximum temperature to be as high as possible while keeping the sample in the solid state and avoiding incipient melting. This is because the dissolution of the major alloying elements is typically of particular interest for heating experiments. Since the heating rate specific solvus temperature rises with increasing heating rate, high maximum temperatures are preferable. A quantitative analysis of the total enthalpy changes during heating can only be carried out if the heating rate specific solvus temperature is exceeded (see below).

**Figure 16:**
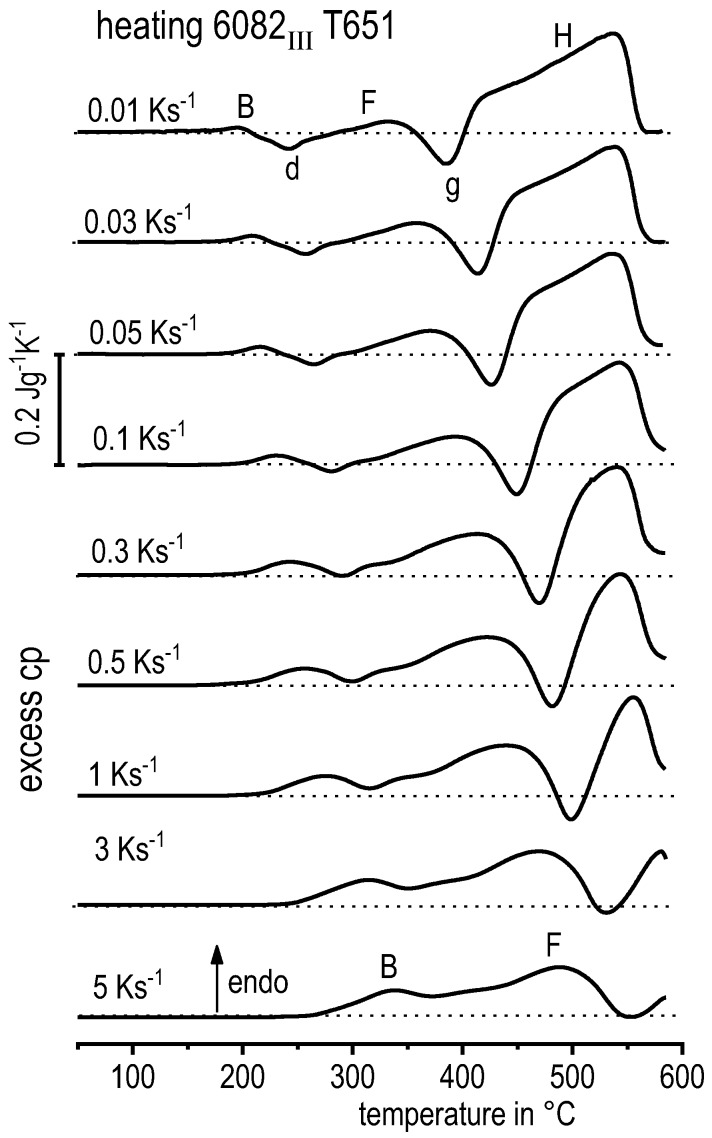
Heating DSC curves of 6082_III_ in initial state T651, [148].

Why are overlapping microstructural reactions a problem in the interpretation of the DSC heating curve? DSC detects (only) the sum of any heat that is released or consumed by the reactions inside the sample. In a theoretical case, a precipitation and a dissolution reaction with equal quantities of enthalpy but opposite algebraic signs may occur, and in this case, the resulting DSC signal would be zero. Thus, a heat flow signal of zero during heating does not necessarily mean that no reactions are taking place. In addition, the peak temperature of a certain heating DSC peak is not necessarily equal to the intensity maximum of the related microstructural reaction [120]. The above-discussed aspects imply that kinetic evaluation methods that focus on single heating DSC peaks, for instance the Kissinger method [147], are problematic for the precipitation hardening of Al alloys. The issue of superimposed reactions can be exemplified by Figure 16, which plots DSC heating curves of 6082_III_ in the initial state T651. During slow heating with 0.01 K/s, there are two smaller dissolution peaks B and F at about 200 °C and 330 °C, respectively. In addition to the fact that the peaks shift towards higher temperatures at higher heating rates, the peak areas also substantially increase. It is very unlikely that a diffusion-controlled dissolution reaction increases its transformed fraction if the time available for that reaction is substantially shortened. The phenomenon of increasing peak area can only be explained by a significant variation in the superposition of different reactions. This may be caused by a more severe suppression of the overlapping exothermic precipitation reaction.

**Figure 17:**
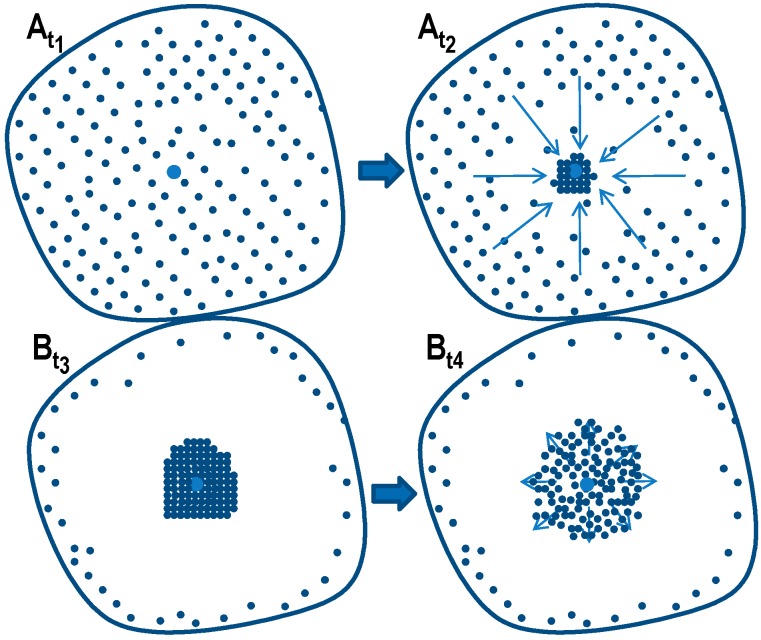
Schemes for (**A**) a precipitation process from a homogeneously distributed solid solution covering time steps t1 to t2; (**B**) dissolution of a precipitate covering the time steps t3 to t4, t4−t3 < t2−t1.

Similar cases with increasing areas of the dissolution peaks at higher heating rates can be found in multiple cases of different age-hardening Al alloys and different initial heat treatment states [120,127,144,149]. One likely reason for the more severe suppression of precipitation compared to dissolution is the different diffusion distances of these reactions. This can be seen in Figure 17, which schematically shows the development of these reactions over time in similarly large microstructure areas. In Figure 17A, showing precipitation from a homogeneously distributed solid solution, the alloying element atoms have to diffuse a certain distance in order to consolidate with one precipitate particle, forming its own crystal structure. This effectively causes the exothermic heat effect of precipitation. In Figure 17B, the dissolution of an existing precipitate only requires the crystal structure of the precipitate to be dissolved in order to provoke the endothermic heat effect. This dissolution of the previously existing precipitate structure requires the alloying elements to diffuse over a much shorter distance; this requires a shorter duration to complete the reaction. Thus, if the time available for the reaction is shortened and we assume that both the dissolution and precipitation of similar phases proceed at very similar or equal diffusion rates, it must be concluded that the kinetic suppression of both types of reactions is similar. However, since the diffusion distances are different, a similar shortening of the reaction time causes a more severe suppression of the precipitation reactions. As a side effect, due to the increased heating or cooling rates (and hence a shortened diffusion time), precipitates tend to become smaller, and this also arises from the reduced diffusion pathways. However, dissolution reactions are also increasingly suppressed with rising heating rates.

It should be stated that new methods need to be developed in the future to evaluate the kinetic data from DSC heating curves. All of the existing methods are based on the assumption that the transformed fraction of alloying elements is independent of the varying heating rates [75,150,151]. This basic assumption obviously does not hold, and this is particularly the case for heating rate variations over a wide dynamic range. This leads to the need for developing new methods in the future for the evaluation of kinetic parameters, a problem which could be solved by kinetic modelling. Some available models even use a combination of both precipitation and dissolution to model the whole DSC heating curve from room temperature up to the solvus temperature (e.g., [152,153,154,155,156]). In the future, these models will need to implement the kinetic suppression of diffusion-controlled reactions.

**Figure 18:**
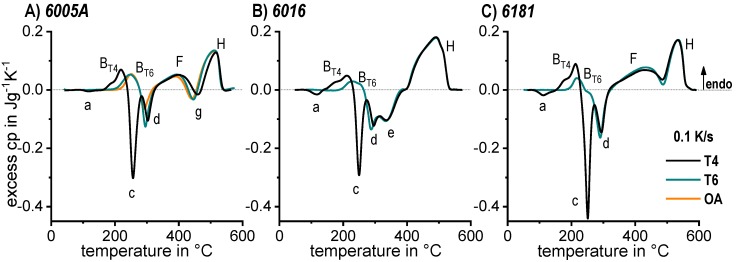
DSC heating at 0.1 K/s for the alloys (**A**) 6005A; (**B**) 6016; and (**C**) 6181 with the initial conditions T4 and T6, and in case of 6005A overaged (solution treated, over-critically quenched and aged at 200 °C for 10 h) [120].

**Figure 19:**
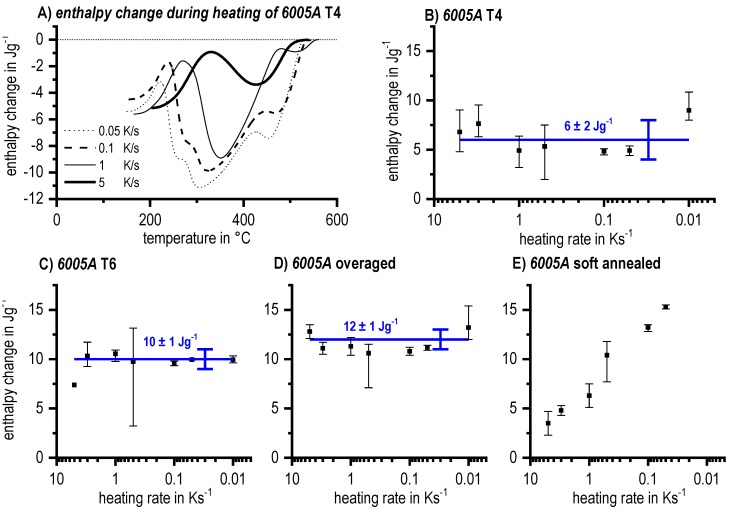
(**A**) Enthalpy change of 6005A T4 during heating to 580 °C; (**B**–**E**) enthalpy levels of 6005A for initial conditions T4 and T6, over-aged and soft annealed, respectively, covering the heating rate range 0.01 to 5 K/s [120].

Despite the abovementioned issues with the analysis of DSC heating curves, there are some aspects that can still be derived from DSC heating curves, such as the following:

- The course of the DSC heating curve strongly depends on the initial state of the sample and the heating rate, e.g., Ref. [75,117,120,127,144,157,158,159,160]. The differences from the initial state are therefore mostly relevant at temperatures below about 300 °C (see Figure 18, which shows heating DSC curves for three AlMgSi alloys in different initial heat treatment states). Above 300 °C, the differences between the DSC curves resulting from different initial precipitation states are mostly equalised, and thus the heating DSC curves are very similar at high temperatures.
- If the maximum temperature of the heating program is chosen to be high enough, and if the alloy happens to allow the complete dissolution of the major alloying elements, the heating rate-dependent solvus temperature can be determined by heating DSC. In Figure 18, this characteristic temperature relates to the end of peak H, where the DSC signal finally drops back towards zero. This aspect has high technological relevance, as determination of the solvus temperature allows us to choose a proper solution treatment temperature. Choosing a solution temperature well above the solvus is crucial in cases where a short soaking is necessarily related to the technological process, for instance in continuous annealing processes during sheet production [148].
- If the abovementioned condition is fulfilled and the DSC heating curves exceed the scanning-rate-specific solvus temperature, the total integral of the DSC heating curve $\int_{T_{min}}^{T_{max}}excess\;cp\;\left(T\right)$ gives the enthalpy level Δh of the initial state. This enthalpy level is an indicator of the thermal and kinetic stability of the initial state. That is, a more stable precipitation state has a larger enthalpy level (6005A: Δh_T4_ = 6 J/g < Δh_T6_ = 10 J/g < Δh_oa_ = 12 J/g, see Figure 19, [120]). In the case of a soft annealed 6005A (Figure 19E), the precondition for complete dissolution during heating to T_max_ does not hold. The determination of the enthalpy level of the initial state can be useful in two different ways: firstly, the heat treatment state of an unknown delivery condition of an alloy can be estimated; and secondly, the determination of the enthalpy level by a reheating experiment allows us to determine the enthalpy changes caused by preceding heat treatments, which is very valuable in order to extend the DSC scanning rate range by indirect experiments, for instance.

#### 3.1.2. Achieving a Complete Solid Solution

Producing a complete solid solution in terms of the dissolution of the major alloying elements of wrought Al alloys has major importance in terms of utilising the full age-hardening potential. In addition, incomplete dissolution will increase the quench sensitivity of an alloy [148]. This is because quench-induced precipitation is accelerated by the higher number of available nucleation sites that are present in the form of undissolved secondary phase particles (of the same or a highly similar phase, which precipitates during cooling). More precisely, if undissolved particles remain, nucleation is not necessary and instantaneous growth can take place. In such cases, the hardness after ageing (the age-hardening potential) can be substantially reduced [148].

DSC analysis can help to identify proper solution treatment parameters. In the first instance, the solvus temperature can be estimated from DSC heating experiments, as discussed above. In this case, the solution temperature is ideally chosen to be a few tens of K above the solvus temperature. If the overheating above the solvus must be kept small, or if very coarse precipitates are to be dissolved, and if the homogeneity of the elemental distribution is also a consideration, a particular soaking duration is required at the solution temperature. In Ref. [161], we showed that the heat-flow development during isothermal soaking in a differential scanning calorimeter at the solution treatment temperature can be used to estimate the duration of the dissolution processes. Based on this, suitable solution treatment parameters can be derived.

#### 3.1.3. Extending the Scanning Rate by Reheating Experiments

The determination of the enthalpy level of any initial state can be utilised in indirect DSC reheating experiments. The basic idea is to perform systematic variations of the initial state via defined heat treatments and the subsequent determination of the enthalpy level by a DSC reheating experiment [117,129,130]. One prerequisite for this approach is that the alloy allows the formation of a complete solution at the solution treatment temperature. The complete solid solution is an equilibrium state, and its enthalpy level h_0_ can be defined as zero. A second prerequisite is that this equilibrium enthalpy state h_0_ = 0 must be reached again during reheating.

**Figure 20:**
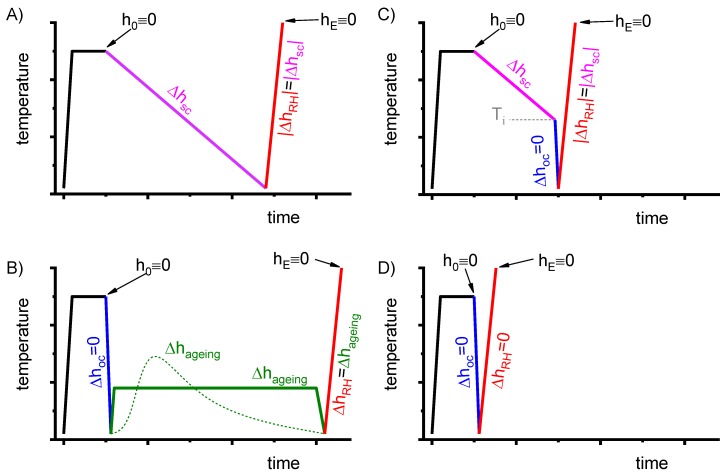
Schematic development of the enthalpy level Δh for different temperature-time profiles with final reheating (RH). (**A**) Slow cooling (sc) from solution treatment to room temperature; (**B**) slow cooling from solution treatment to a certain temperature and interruption by overcritical cooling; (**C**) arbitrary types of ageing after overcritical cooling from solution treatment; and (**D**) overcritical cooling (oc) from solution treatment.

Figure 20 schematically illustrates some potential applications of this basic idea. In Figure 20A, an enthalpy change Δh_sc_ due to quench-induced precipitation during slow cooling is assumed. Since the reheating step ends in an enthalpy state h_0_ = 0, the same magnitude of enthalpy change (with an opposite algebraic sign compared to cooling) must be exchanged during reheating. Reheating can therefore reveal the enthalpy change caused by precipitation during slow cooling. Reheating experiments after variations of the “slow” cooling rate thus allow for the determination of precipitation enthalpy as a function of the cooling rate [117,130]. Since “slow” cooling in this case refers to the capability of the calorimeter used, reheating can be applied to extend the cooling rate range in both directions, i.e., both slower and faster experiments. In both cases, quantitative statements of the enthalpy changes are possible [117,129]. We performed slow cooling in a controlled air furnace with rates down to 3 × 10^−5^ K/s, corresponding to a cooling duration (540 to 20 °C) of more than half a year [132]. On the other side of the spectrum, using the chip-sensor based DFSC, “slow” cooling may be as fast as several hundreds of K/s [129,130]. These DFSC devices commonly require scanning rates above 1,000 K/s to obtain a sufficient signal to noise ratio [134]. Thus, reheating in a DFSC is typically carried out at rates above 1,000 K/s [129,130,162]. The reheating approach can be extended to a temperature-dependent determination of the precipitation enthalpy by interrupting the precipitation process during “slow” cooling at a certain temperature T_i_ (see Figure 20C) [129].

Another form of this idea involves the systematic variation of a particular heat treatment, for instance an ageing treatment, prior to reheating (see Figure 20B). If cooling from solution treatment is carried out faster than the UCCR, the subsequent reheating step will give a total enthalpy change of zero [117,130] (see Figure 20D). This fact can be utilised as a baseline correction for the preceding reheating experiment [129,130]. Based on this idea, the differential reheating method (DRM) was introduced in Refs. [130,162], an approach that allows the application of chip-sensor-based DFSC for the analysis of the kinetics of quench-induced precipitation in Al alloys. In particular, the specific precipitation enthalpy can be obtained as a function of the cooling rate, allowing us to determine the UCCR of the alloy. In Ref. [129], the DRM was developed further towards the obtainment of the specific enthalpy changes depending on the temperature. The latter allowed assessment of the start and end temperatures of certain precipitation reactions, which helps in completing the CCP diagrams for highly concentrated alloys with high UCCRs [129].

**Figure 21:**
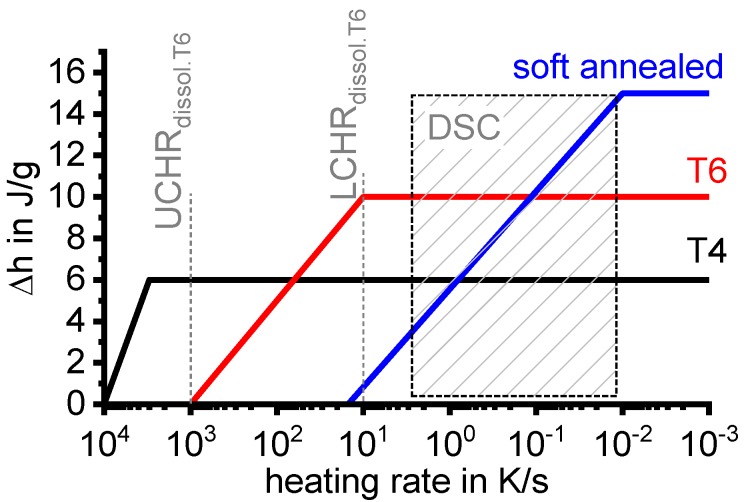
Schematic development of the enthalpy levels of three different heat treatment states in a medium-strength AlMgSi alloy obtainable by reheating to a certain maximum temperature. Values are based on those of 6005A in [120]. The shaded area signifies the heating rate range covered by DSC in Ref. [120].

The above considerations lead to the postulation of two critical heating rates for the dissolution of a specific initial heat treatment state, as illustrated in Figure 21. The first of these is the lower critical heating rate (LCHR), and the second is the upper critical heating rate (UCHR). The LCHR is postulated to be the fastest heating rate at which the particular initial state can be dissolved completely during heating to a certain maximum temperature T_max_ (which must be above the solvus temperature of the alloy). A potential further increase in this temperature in the solid state will reduce the LCHR. If heating is carried out to T_max_ at a rate slower than the LCHR, a complete solid solution will be obtained during heating. The LCHR therefore has high technological importance. For a soft annealed state of 6005A, we found indications that the LCHR is on the order of 0.01 K/s, although there is no extensive experimental proof of this. The UCHR is theoretically defined as a fast heating rate at which diffusion is prevented completely and no dissolution takes place. Currently, for the soft annealed 6005A, a UCHR can only be estimated as about 10 to 20 K/s (compare Figure 19E with Figure 21).

### 3.2. Quench-Induced Precipitation during Cooling from Solution Treatment

#### 3.2.1. AlSi Binary Wrought Alloys

**Figure 22:**
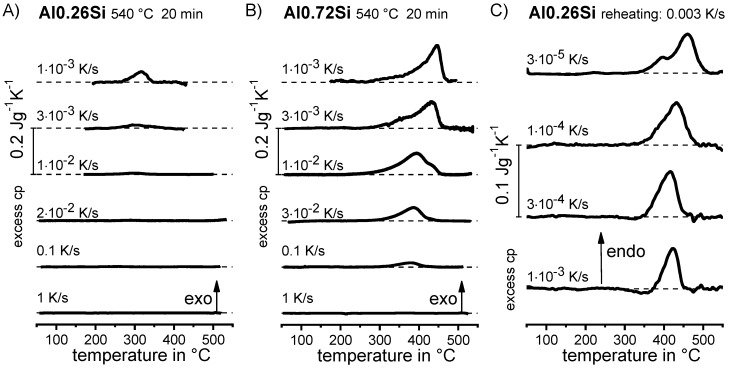
Direct cooling DSC curves of (**A**) Al0.26Si and (**B**) Al0.72Si in a range of cooling rates from 1 to 10^−3^ K/s; (**C**) DSC reheating curves of Al0.26Si after cooling at rates down to 3 × 10^−5^ K/s.

**Figure 23:**
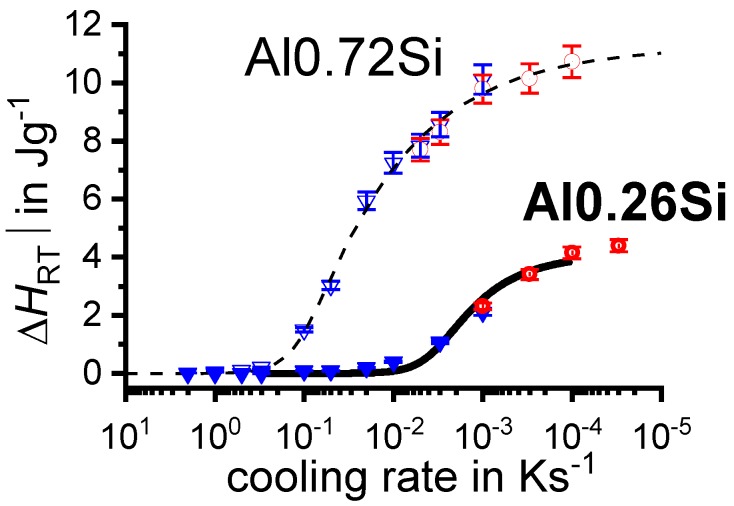
Enthalpy development by quench-induced precipitation. Combination of directly (blue triangles) and indirectly (red circles) measured results. Predictions obtained from model in Ref. [117].

Figure 22A,B show direct cooling curves for alloys Al0.26Si and Al0.72Si, while Figure 22C displays DSC reheating curves for Al0.26Si after various extremely slow cooling rates were applied. The specific precipitation enthalpy values are given in Figure 23.

From Figure 22A,B and Figure 23, it can be seen that even in lean pure binary Al-Si alloys, quench-induced precipitation is separated into high-temperature (HTR) and low-temperature (LTR) reactions. For Al0.26Si, the HTR is detected only after extremely slow cooling at 3 × 10^−5^ K/s (seen as a two stage dissolution reaction during reheating in Figure 22C). The UCCR of Al0.72Si was found to be 1 K/s [117,132], while for Al0.26Si this is only 3 × 10^−2^ K/s [132].

Analysis using SEM and HR-TEM with EDS and SAED/NBD [132] showed that both reactions were caused by the precipitation of the same phase, i.e., the Si phase with diamond cubic crystal structure, but that the morphologies and dimensions were different: during HTRs, polygonal particles with a low aspect ratio grew (up to several tens of µm in diameter), while during LTRs, plate-like particles with a high aspect ratio were formed (up to several µm in length but only several hundreds of nm or less in thickness) [117,132].

From Figure 23, it can be seen that the precipitation enthalpy obtained directly from in situ cooling DSC and the values from indirect DSC measurements fit together very well, thus providing additional confirmation of the validity of these approaches. In Ref. [117], it is demonstrated that the specific precipitation enthalpy shown in Figure 23 is directly proportional to the volume fraction of quench-induced Si precipitation. The continuous lines plotted in Figure 23 in addition to the measured data points are the results of a model (for details, see [117,132]). This model allows us to accurately calculate the volume fraction of quench-induced Si precipitation as well as the fraction of Si left in the solid solution for various temperatures and cooling rates.

**Figure 24:**
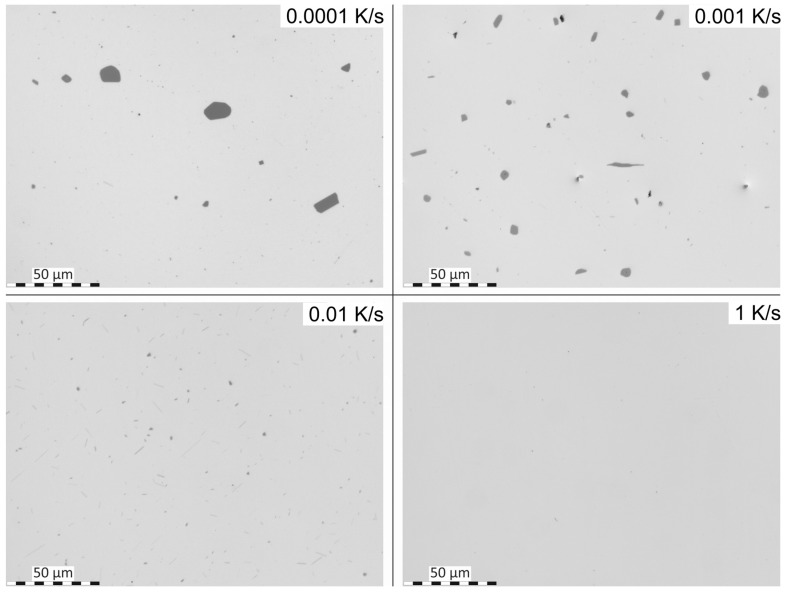
OM images of “quench-induced” precipitation in Al0.72Si after cooling at different rates.

**Figure 25:**
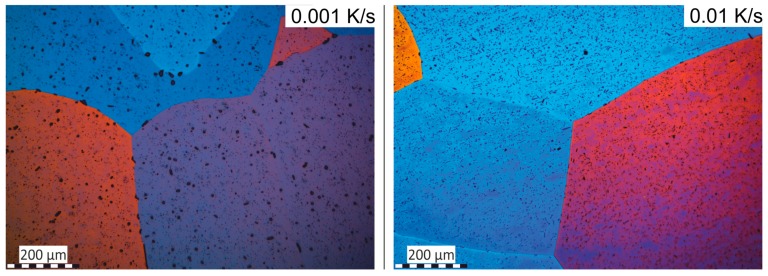
OM images of Barkers-etched Al0.72Si samples after two different cooling rates, revealing that the vast majority of quench-induced precipitation occurs inside the grains.

**Figure 26:**
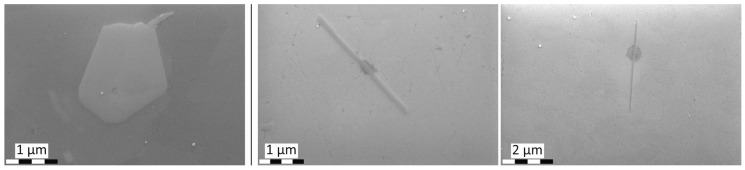
SEM-SE images of quench-induced precipitates in Al0.72Si after cooling at 0.01 K/s. Left: Polygonal particle originating from the HTR; middle and right: needle-/plate-shaped particles from the LTR [132].

**Figure 27:**
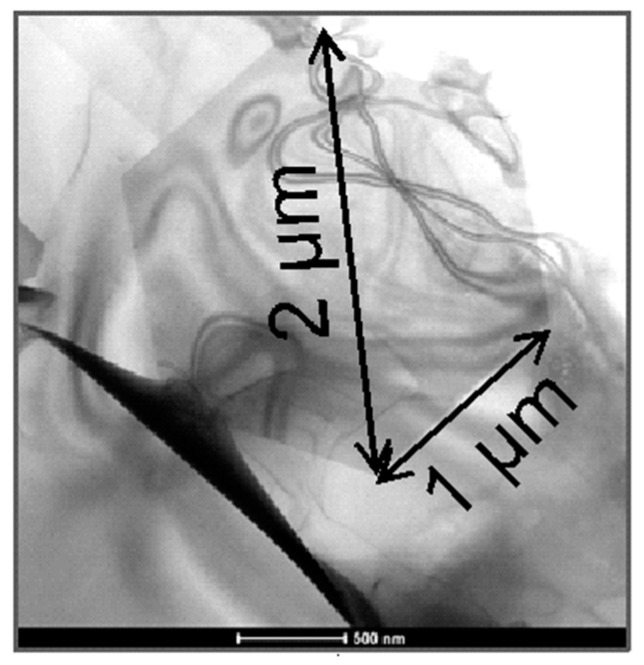
BF-STEM images of hexagonal Si plate in Al0.72Si after cooling at 0.1 K/s, zone axis [001]_Al_ [132].

**Figure 28:**
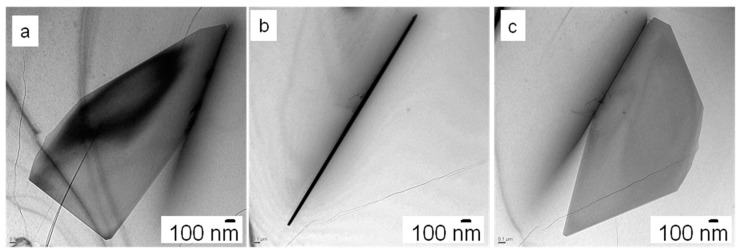
BF-STEM images of a thin plate formed from cooling at 0.1 K/s (pure Si, diamond cubic), standing out from the Al matrix in three different rotations: (**A**) −40°; (**B**) 0° (zone axis [001]_Al_); and (**C**) 40° [132].

Figure 24 and Figure 25 display microstructural images depending on the cooling rate in Al0.72Si. Optical microscopy after very slow cooling with 10^−4^ K/s (Figure 24) reveals polygonal-shaped Si particles. If cooling is carried out ten times faster, at 10^−3^ K/s, the number of quench-induced particles is substantially increased (Figure 24B), while their particle size is reduced. From Figure 23, it can be seen that the volume fraction of quench-induced precipitation is similar for these two very slow cooling rates. In Ref. [117,132] we demonstrated that these polygonal-shaped precipitates originate from the HTRs seen in cooling DSC. At a cooling rate of 10^−2^ K/s, quench-induced particles are further reduced in size. A detailed analysis of the OM images reveals that next to the polygonal-shaped particles, further plate-like particles (appearance: needle or rod-shaped) with distinct orientations appear (see also Figure 26). It has been proven that these plate-shaped particles originate from the LTRs [117,132]. After cooling at 1 K/s, no quench-induced precipitates can be seen in OM. Figure 25 demonstrates that the vast majority of quench-induced precipitates form inside the grains. In high-purity alloys such as this one, grains are generally coarse. The OM results are consistent with the DSC results in Figure 22, indicating that at 0.01 K/s, the HTR has already been suppressed to a great extent. However, at this cooling rate, and for even faster cooling at 0.1 K/s, TEM unambiguously shows that some precipitates form during the quench (see Figure 27 and Figure 28).

In Figure 26, the individual particles that form in the HTR and LTR are compared using SEM. SEM-EDS and TEM-EDS reveal that both the needle-/plate-shaped and polygonal particle types are nearly pure Si. Selected area electron diffraction (SAED) in TEM additionally reveals that both particle types have the same diamond cubic lattice structure. Figure 27 shows a BF-STEM image of a quench-induced Si plate-shaped precipitate (formed during the LTR) after cooling at 0.1 K/s. Under this cooling condition, these Si plates have thicknesses of about 50 nm, with an edge length of 5–20 µm. Via TEM experiments employing sample rotation (see Figure 28), it was confirmed that particles such as those shown in Figure 26 (middle and right) and Figure 27 are indeed plate-shaped.

At first impression, it may seem odd that pure binary AlSi shows two quench-induced precipitation reactions in which the same phase is formed. However, it has been reported earlier that Si particles in Al can precipitate either as equiaxed particles (here, polygonal particles) or as hexagonal plates [163,164].

During cooling, competing effects occur, as the increasing supersaturation with decreasing temperature leads to an increasing driving force for precipitation, while the precipitation itself depletes the matrix, leading to a slowed reaction. In addition, the diffusion rate is steadily reduced as cooling progresses, and the lattice misfit between precipitate and matrix changes due to the differing thermal contraction of Al and Si. Indeed, the thermal expansion coefficient of Si is much lower than that of Al, with the difference being higher than most other light-alloy–precipitate combinations. These effects can combine to cause the same phase to form in two morphologies, as also shown for Ni-based superalloys, for instance [165,166,167,168].

During cooling of the binary AlSi alloys, high and low-temperature precipitation compete for the same alloying element (i.e., Si). If cooling is carried out very slowly, precipitation will primarily result in coarse, polygonal-shaped particles being formed during the HTR. As the majority of those particles are precipitated inside the grain, homogeneous nucleation can be expected, particularly since this is promoted by the vacancies that are expected to be present at higher temperatures [132]. If precipitation at high temperatures is suppressed to a certain extent, a fraction of the solved Si will be precipitated at lower temperatures. From the classical theory of nucleation and growth, it is known that the morphology of precipitates is dependent on the formation temperature [169]. At high temperatures, the phase transition is mainly inhibited by the formation of new interfaces. Since polygonal-shaped particles have a lower surface-to-volume ratio, precipitation of these particles is preferred at high temperatures [132]. At lower temperatures, the lattice distortion mainly inhibits the precipitation, and this is why high aspect ratio particles preferentially precipitate at lower temperatures [132].

The nucleation mechanism of the low-temperature plate particle precipitation is not yet fully resolved. Figure 26 (middle and right image) show that plate-shaped Si particles with a length of several µm have nucleated on an equiaxed particle of about 200 nm diameter. It therefore appears that at a cooling rate close to the critical cooling rate, some of the plate-shaped particles forming in the LTR can nucleate on fine Si particles formed in the HTR. However, this is only possible if there is a sufficient driving force for precipitation during the LTR in the vicinity of the equiaxed particle, i.e., if the HTR reaction is incomplete and leaves a substantial supersaturation of Si in the Al-rich phase near the small equiaxed particle. This explains why this type of nucleation of plate-shaped particles is only seen for cooling rates close to the critical cooling rate.

**Figure 29:**
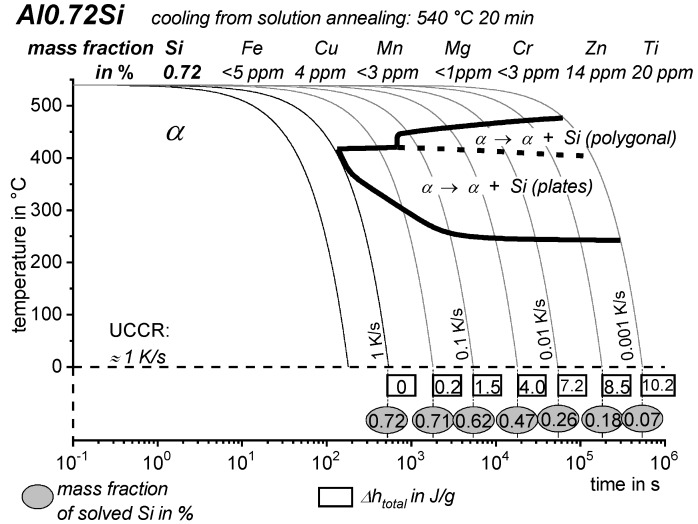
CCP diagram of Al0.72Si. Instead of hardness values, the mass fraction of Si supersaturation is stated.

The results obtained for quench-induced precipitation of the binary Al0.72Si alloy are summarised in the CCP diagram shown in Figure 29. As this binary Al-Si alloy is not an age-hardening alloy, no hardness values are shown. Instead, the measured mass fraction of Si remaining in (supersaturated) solid solution after cooling is stated.

#### 3.2.2. 6xxx AlMgSi Wrought Alloys

In this work, a total of 10 different AlMgSi wrought alloys are investigated, none of which contain substantial amounts of Cu. The set of AlMgSi alloys includes two high-purity ternary alloys and eight commercial variants, which also contain further alloying additions and impurities besides Mg and Si, such as Mn, Fe and Cr.

**Figure 30:**
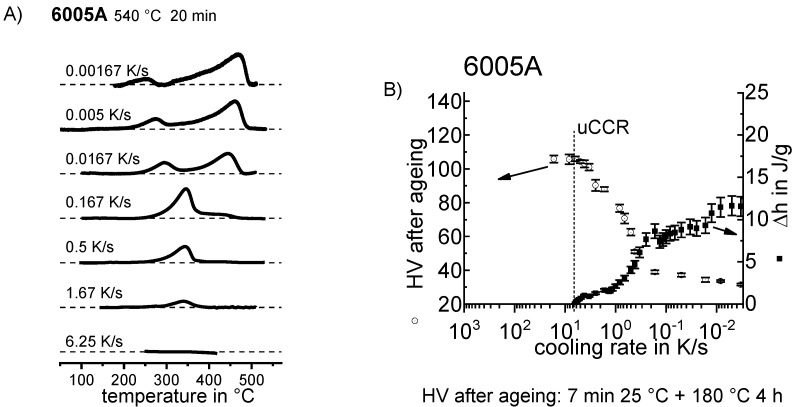
(**A**) Cooling DSC curves for 6005A; and (**B**) related specific precipitation enthalpy after cooling and hardness after subsequent ageing.

**Figure A1:**
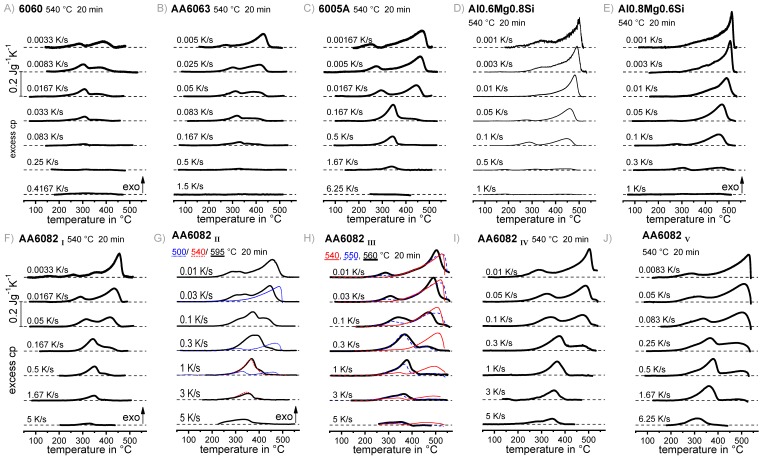
Comparison of DSC cooling curves for 10 different AlMgSi alloys.

Figure 30A presents DSC cooling curves for 6005A, showing effects that are typical for AlMgSi alloys. Two dominating reaction regions are seen, namely the HTRs and LTRs [118,128,138]. A comparison of the DSC cooling curves of the 10 AlMgSi alloys in Figure A1 in the Appendix A (partly published in Refs. [118,128,138]) reveals that these two reaction regions dominate during cooling of all AlMgSi alloys. However, as will be discussed later, several additional and superimposed reactions are also seen.

HTRs are the dominating heat effects during slow cooling, and are increasingly suppressed at increasing cooling rates. At cooling rates where the HTRs are substantially suppressed, the LTRs of the eight commercial alloys studied become more dominant, and the peaks shift towards higher temperatures. These two effects can be explained by the higher concentration of alloying elements that is left in solution prior to the LTR. The high-purity alloys behave differently in this respect from the commercial alloys, and one major reason for this is the difference in the nucleation mechanism of the LTR (shown below in more detail). At even higher rates, the LTRs also experience increasing suppression. For 6005A, a complete suppression of precipitation during cooling was achieved at about 6 K/s. From Figure 30B, it can be seen that the total specific precipitation enthalpy Δh measured by DSC drops to zero at the UCCR. It can also be observed that at higher cooling rates, the hardness after ageing reached a saturation level.

**Figure 31:**
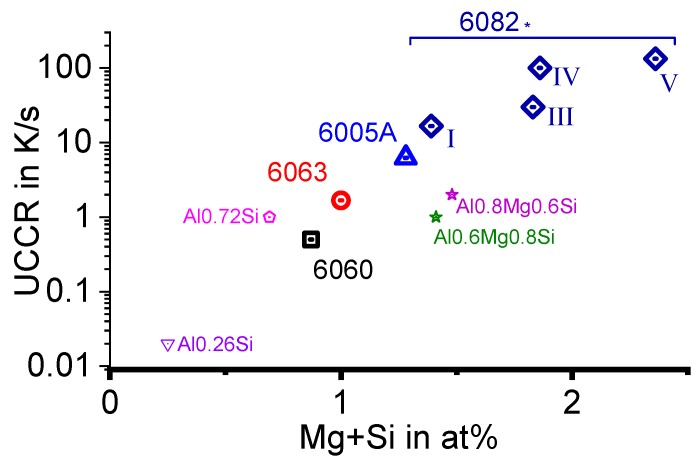
Upper critical cooling rates for nine different AlMgSi alloys and two binary AlSi alloys as a function of their total Mg+Si content.

**Figure A2:**
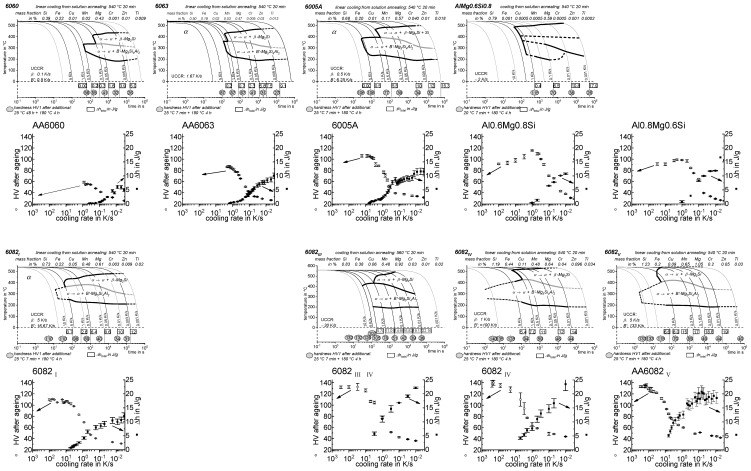
Comparison of the continuous cooling precipitation diagrams, the total specific precipitation enthalpies after cooling and hardness after additional artificial ageing, for 9 different AlMgSi alloys.

As can be seen from the comparative plot of hardness after ageing and specific precipitation enthalpy during cooling for all considered AlMgSi alloys in Figure A2 in the Appendix A, a correlation between precipitation enthalpy and hardness after ageing is shown for all AlMgSi alloys for which DSC was able to achieve sufficiently fast cooling. Thus, in cases where DSC is not fast enough, the hardness data after ageing can provide a good approximation of the UCCR. Figure 31 shows the values of the UCCR on a logarithmic scale for nine different AlMgSi alloys as a function of the summarised Mg+Si content. This figure shows that the UCCR increases substantially with increasing alloying element concentration. Figure 31 also includes four different alloys within the chemical specification of 6082 (which is very broad), and it is interesting to note that their UCCRs can vary by a factor of up to 10. A strong batch influence can therefore be expected in terms of the quench sensitivity of Al alloys with a broad specification range for their composition.

The obtained UCCRs for 6082_I_ and 6063 correspond well with the results obtained in Ref. [23]. In [23], an UCCR of 1 K/s for 6063 is reported, which is the same as that found in this work for a slightly higher concentrated version of 6063 [128]. For 6082, Ref. [23] reports an UCCR of above 10 K/s. The amounts of Mg and Si in the 6082 variant considered in [23] is close to those of the 6082_I_ in this work, for which we obtained an UCCR of ≈17 K/s.

**Figure 32:**
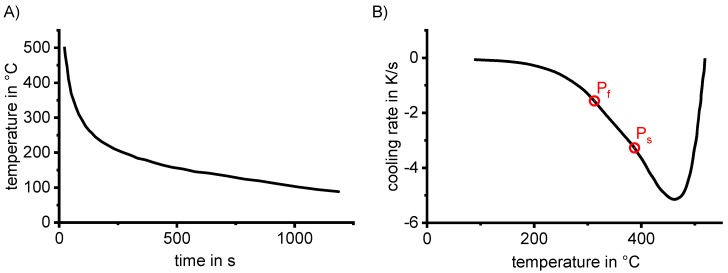
(**A**) Cooling resulting from placing one end of the experimental bar in water, measured at a distance of 5.5 cm from the bar end; (**B**) “instantaneous cooling rate as a function of temperature (as given in A). The points indicate the start (Ps) and end (Pf) of precipitation [25].” Digitized data from Ref. [25].

**Figure 33:**
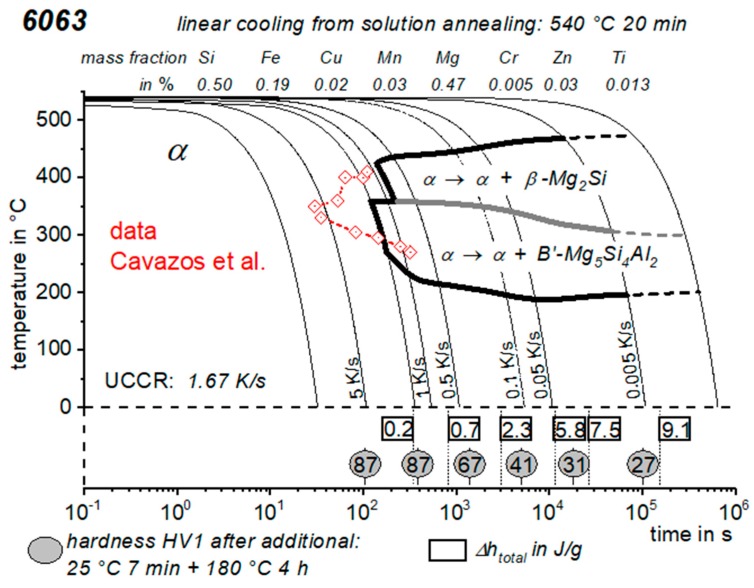
CCP diagram for 6063 obtained by DSC [128] comparing the data points (red) of Ref. [25].

Cavazos and Colás obtained a CCP diagram for 6063 from Jominy-like bar-end-quench tests [25]. They measured the actual cooling rates achieved at different distances of the quench bar end (see the example in Figure 32A for a distance of 5.5 cm). The characteristic transformation temperatures were evaluated from the inflection points of the cooling rate curve shown in Figure 32B. Using this approach, these authors were able to construct a CCP diagram. The 6063 variant considered in Ref. [25] contained mass fractions of 0.50% Si, 0.52% Mg, 0.21% Fe and 0.022% Cu, i.e., very similar to our 6063 variant [128]. The CCP diagrams obtained in Refs. [25] and [128] are compared in Figure 33. Although the 6063 variant in [25] showed slightly faster quench-induced precipitation, it can be seen that the nose temperature is very similar. Considering the sequence of HTR and LTR, it can be concluded that the DSC data are much more detailed, and cover a substantially wider cooling rate range. The authors of Ref. [25] found the UCCR of 6063 to be ≈10 K/s [25], while in Ref. [128] an UCCR of only 1 K/s was found. The higher UCCR of the 6063 variant analysed in Ref. [25] compared to that of Ref. [128] again shows that the quench sensitivity of different batches of alloys that are nominally the same may be substantially different.

As can be seen from Figure 31, the two laboratory alloys Al0.6Mg0.8Si and Al0.8Mg0.6Si form an exception in terms of their UCCR: they have much lower values compared to commercial alloys with comparable Mg and Si contents. This is due to the substantially lower concentration of nucleation sites for quench-induced precipitation, as demonstrated below.

**Figure 34:**
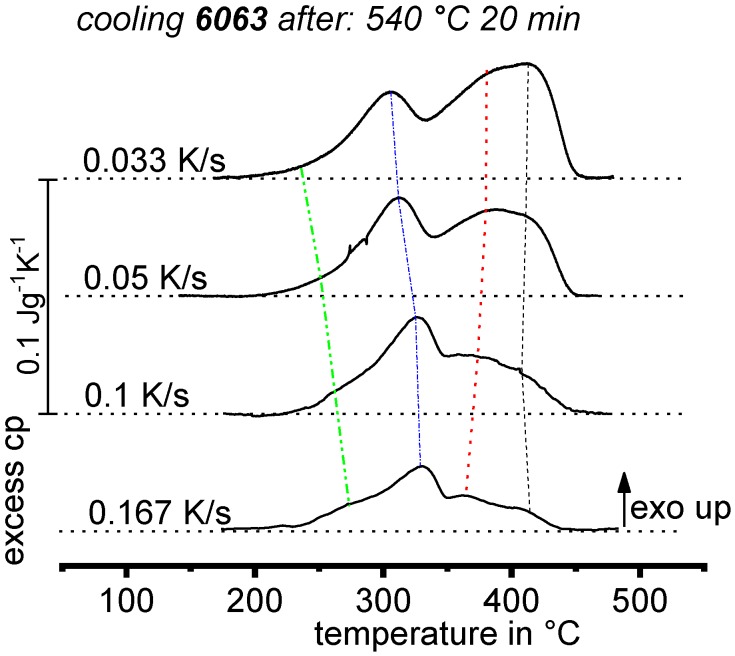
Cooling DSC curves for 6063 in a narrow range of cooling rates at higher excess cp magnification, indicating at least four separate peaks below the two major reaction regions.

For several AlMgSi alloys, DSC indicates the superposition of several microstructural reactions beneath the HTRs and LTRs. This is exemplified by Figure 34 for 6063, and can be also seen for instance in Figure A1F,G, and Figs. 40–41 in Ref. [116].

**Figure 35:**
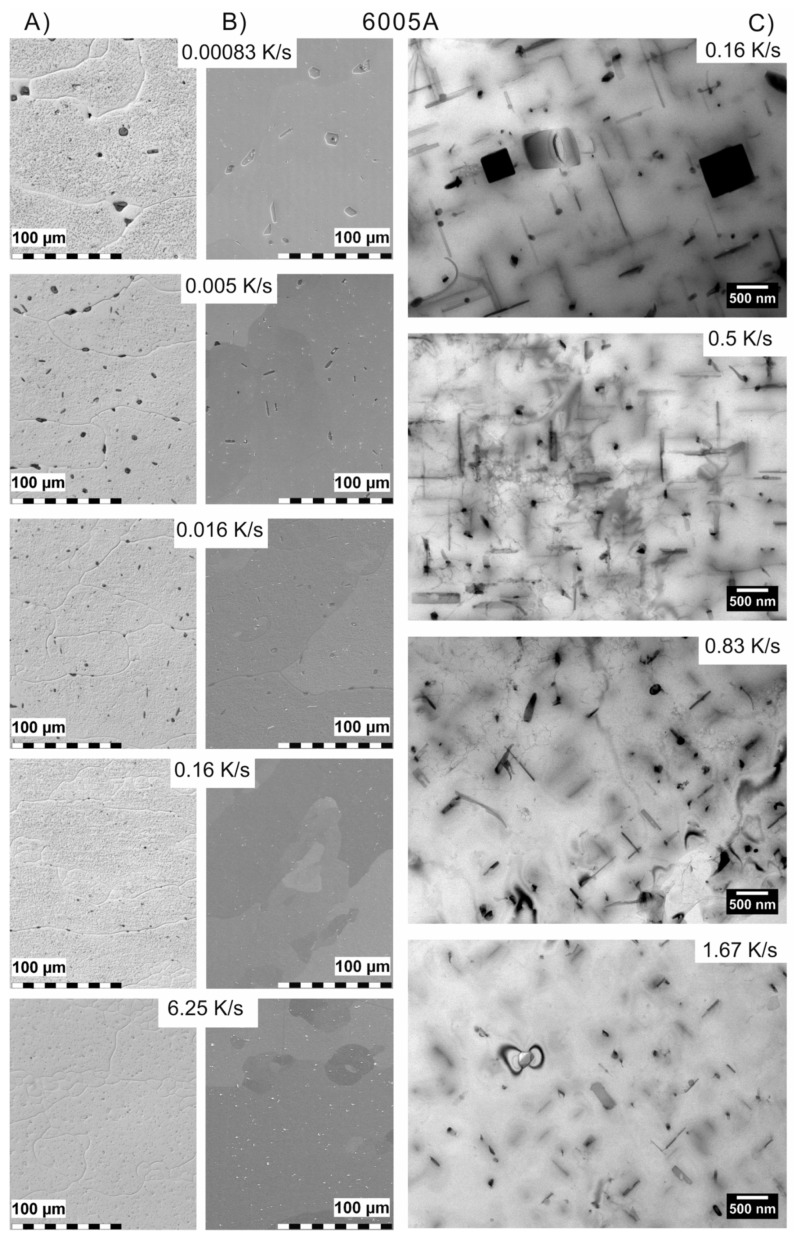
Quench-induced precipitation in 6005A after different cooling rates [128]: (**A**) OM and (**B**) SEM secondary electron micrographs of quench-induced β-Mg_2_Si particles originating from the HTRs; (**C**) TEM images of quench-induced rod-shaped B’ or β’ particles. In each case, the particle dimensions are substantially reduced with increasing cooling rate.

To identify these overlapping microstructural reactions, a relatively large body of work is available, e.g., [23,116,118,128,138,170,171,172,173]. Figure 35 illustrates the authors’ own work on quench-induced precipitation in 6005A at different cooling rates, showing optical micrographs (A), SEM secondary electron images (B) and TEM bright-field images (C) after cooling from solution treatment to the ambient temperature at different rates. It can clearly be seen from Figure 35A,B that precipitates with lower aspect ratio (appearing dark) become substantially smaller as the cooling rate rises. These HTR-quench-induced precipitates are β-Mg_2_Si phase particles, which partially precipitate at grain boundaries and mainly precipitate inside the grains (see Figure 35A,B). The fcc structure of β-Mg_2_Si has been proven by electron backscatter diffraction (EBSD) and X-ray diffraction (XRD) analysis [116,138]. At a cooling rate of 0.16 K/s, hardly any quench-induced precipitation is seen in OM and SEM; however, at this cooling rate, a large number of rod-like quench-induced precipitates with much higher aspect ratios is found in TEM (Figure 35C). The lengths of these rod- and lath-shaped secondary precipitates were observed to decrease from about 600 nm after cooling at 0.16 K/s to about 300 nm at a cooling rate of 1.6 K/s.

**Figure 36:**
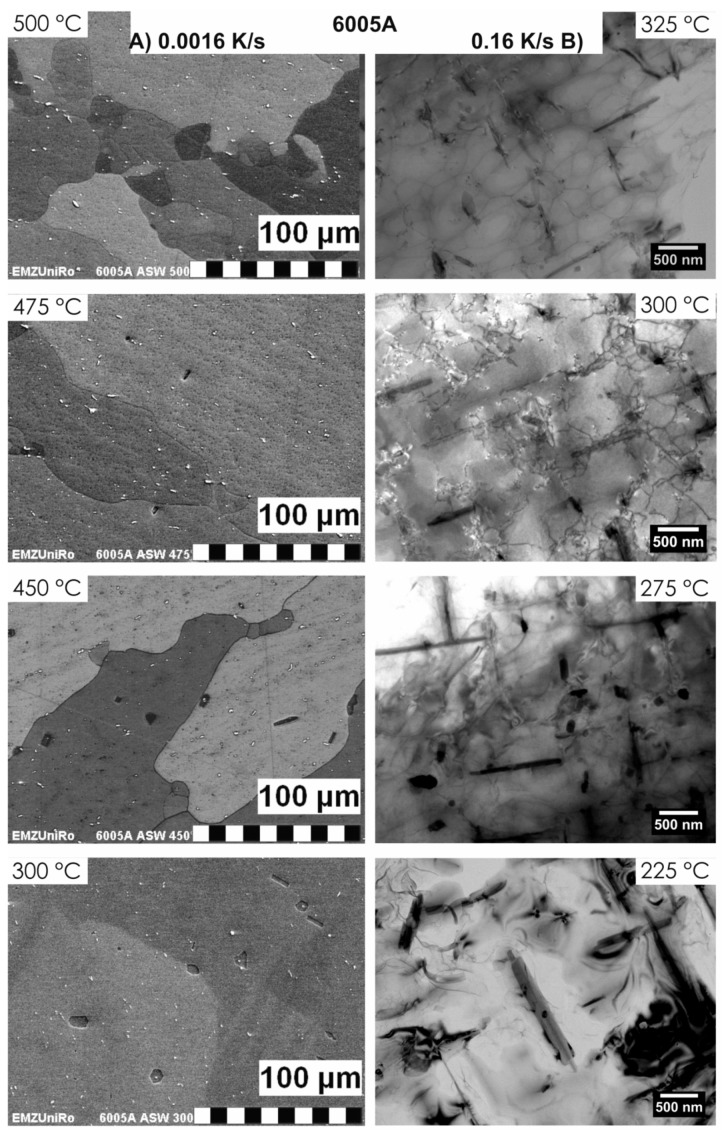
Microstructure of 6005A alloy samples investigated at different temperatures: (**A**) using SEM backscattered electron images at a cooling rate of 0.0016 K/s; and (**B**) using bright-field TEM at a cooling rate of 0.16 K/s [128].

Figure 36A shows BSE-SEM images of the microstructural development of 6005A observed at a constant cooling rate of 0.0016 K/s down to different temperatures (Figure 14). At 500 °C, only primary precipitates are visible. The first Mg_2_Si particles were detected at 475 °C, immediately before the intensity maximum of the HTR was reached, as shown in Figure 30A. A high fraction of Mg_2_Si precipitates was observed by SEM at 450 °C after passing the peak of the HTRs. As revealed by SEM, the microstructure did not change significantly with a further decrease in temperature. It was therefore concluded that the Mg_2_Si precipitation originates from the HTRs. During faster cooling of 6005A at 0.16 K/s, where the LTR dominates (Figure 30A), rod-/lath-shaped precipitates could be detected for the first time at 325 °C. At temperatures below 325 °C, the rod-/lath-shaped precipitates grew only slightly, as illustrated in the TEM micrographs in Figure 36B. Hence, it was concluded that the precipitation of rod-/lath-shaped precipitates corresponds to the LTR.

**Figure 37:**
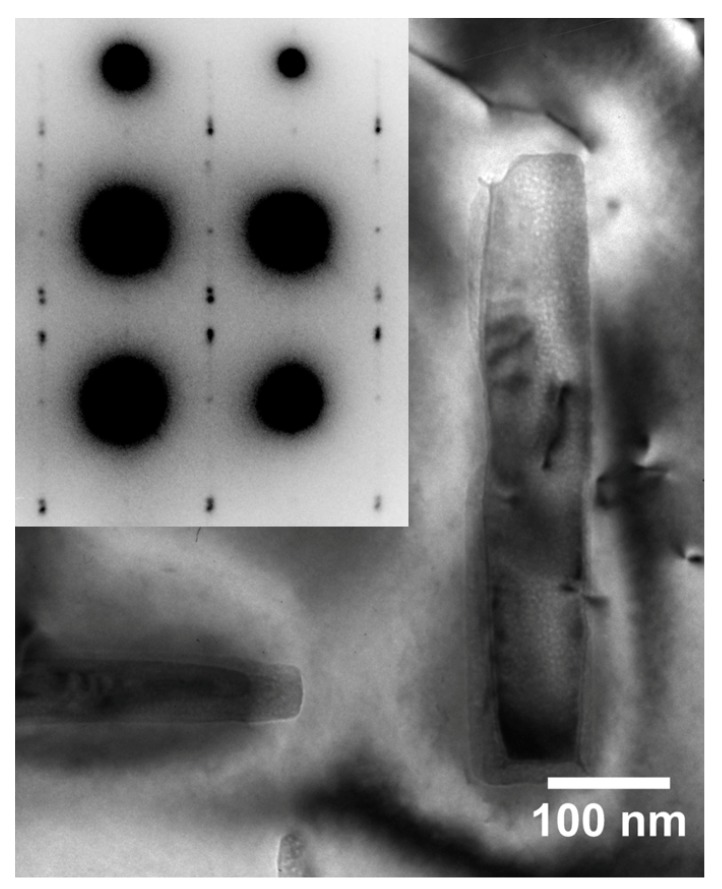
Bright-field TEM micrograph of a 6005A specimen after cooling at 0.16 K/s [128].

Figure 37 shows a TEM bright-field micrograph of a rod-shaped precipitate embedded in an Al matrix. The corresponding SAED pattern of the [001] Al zone axis is shown in the inset. Additional superlattice reflections initiated from the rod precipitate in the SAED pattern are visible. Rod- and lath-shaped precipitates show similar diffraction patterns in the [001] Al zone axis. Since parts of the diffraction pattern initiated from the rod-/lath-shaped precipitates are located at the <100> positions of the aluminium matrix, these precipitates are coherent with the Al matrix along this direction. The diffraction pattern of the [001] zone axis of Al indicates superlattice reflections (Figure 37) that are very similar to those reported in the literature for the same kind of precipitates [174,175,176,177,178,179]. However, the interpretation of these results differs significantly between authors. While the authors of Refs. [174,176,177,178] suggest a hexagonal structure based on the TEM diffraction pattern, the authors of Refs. [179] report a monoclinic structure determined from similar diffraction images. Furthermore, it has been found that both the hexagonal and the monoclinic crystal structure of these precipitates build upon the same Si-based network structure [179]. Due to the coherence of the rod- and lath-shaped precipitates observed along the <100> direction of the aluminium matrix, at least one lattice parameter is known (0.405 nm), which is in agreement with the reported values of the hexagonal structure for β’ (a = 0.705 nm, c = 0.405 nm) and B’ (a = 1.03 nm, c = 0.405 nm) [174,175,176,177]. According to [5], both types of precipitates, β’ and B’, can exist concurrently, although B’ occurs particularly at high Si:Mg ratios. Hence, it can be assumed that the low-temperature precipitates are β’ and/or B’. A recent work [180] confirms the quench-induced precipitation of the hexagonal β’ phase during the LTR due to a combination of cooling DSC, TEM, EDS and SAED in an Mn-rich dispersoid-containing model alloy with a composition of Al-0.67Si-0.84Mg-0.35Mn-0.25Fe (mass fraction in %).

Figure 30 shows that at cooling rates faster than 0.5 K/s, the start of the LTR for 6005 is about 400 ± 10 °C, and the peaks of this reaction for all cooling rates faster than 0.16 K/s are located at about 320–340 °C. According to the solvi temperatures determined from first principles modelling in [181], this onset temperature is about 90 K below the solvus of the β’-phase. Hence this reaction is unlikely, due to formation of the β’-phase. Instead, the start temperature corresponds closely (within 10 K) to the solvi temperatures of the hexagonal Al_2_MgSi_2_ phase, the hexagonal Al_4_Mg_8_Si_7_ phase and the orthorhombic Al–Mg–Si phase [181]. It has been shown that the Mg:Si ratio in the hexagonal precipitate formed at these temperatures [176,182] is close to 1.15 [176], and hence the dominant reaction is thought to be the formation of this Al_4_Mg_8_Si_7_ phase, which is termed B’ (for further discussion, see [118]). The length of LTR B’ particles is on the order of several hundreds of nm, while their aspect ratio is about 10 [116]. The chemical composition of the rod-/lath-shaped precipitates consists of Al, Si and Mg, and sometimes Cu, as measured by TEM-EDS [128]. In most cases, more Si than Mg was detected; however, the chemical composition varied between the individual precipitates.

**Figure 38:**
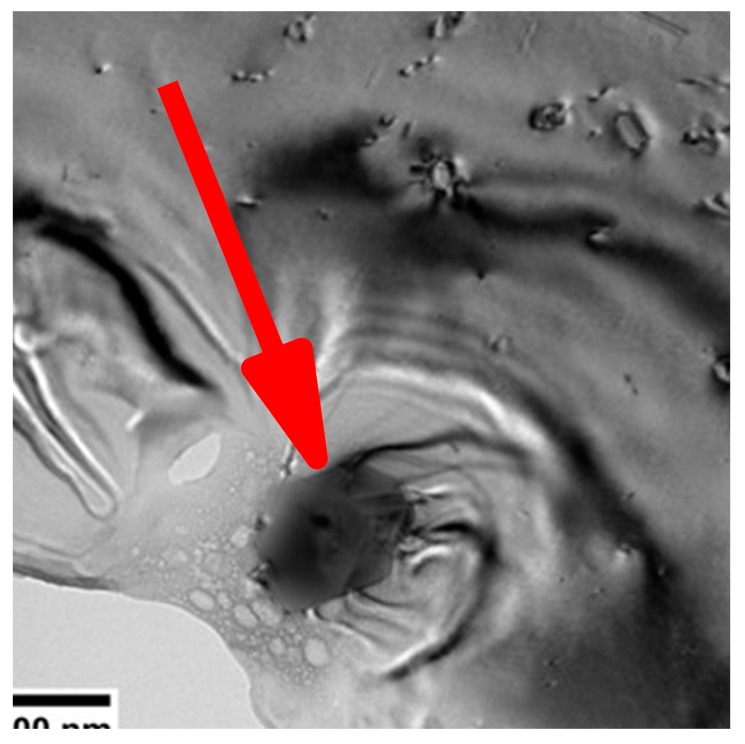
BF-TEM images of a quench-induced hexagonal Si plate particle, precipitated in 6005A cooled from 540 °C 20 in at 0.167 K/s to 375 °C.

In addition to the formation of β-Mg_2_Si and B’ precipitates, pure Si particles were found after relatively slow cooling, as shown by the particle in Figure 38 for which EDS analysis revealed nearly pure Si (EDS results not shown here). Regarding the precipitation of pure Si in an AlMgSi alloy with excess Si relative to the stoichiometric composition of Mg_2_Si, it is reasonable to compare the DSC cooling curves of AlMgSi alloys with those of pure binary AlSi alloys. For the latter, it can be seen that both the HTR and the LTR occur within a temperature range of ≈460–300 °C at 0.01 K/s. When the same cooling rate for 6005 is compared in the same temperature range, only the HTR peak is seen. In terms of the overlapping reactions beneath the HTRs and LTRs, it can be assumed that the precipitation of Si adds a certain heat effect to the HTRs.

**Figure 39:**
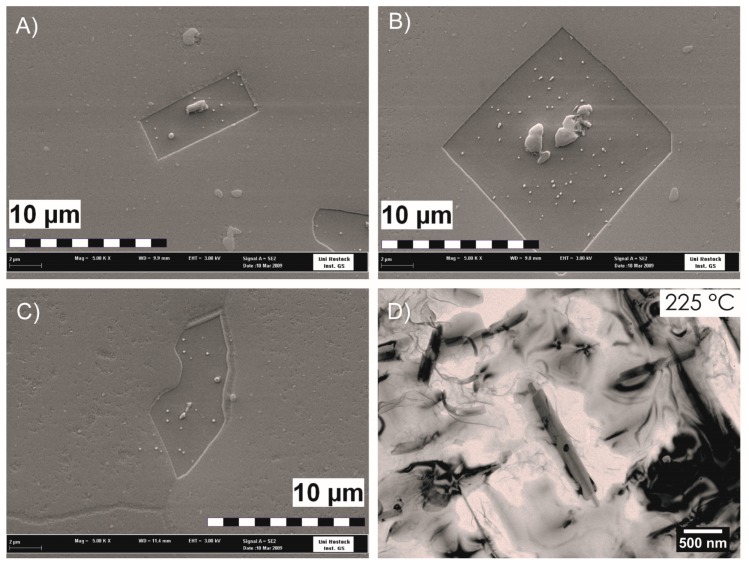
Microstructural details of 6005A: (**A**–**C**) SEM secondary electron images of Mg_2_Si particles after very slow cooling (0.00083 K/s), which all seem to be nucleated on coarse primary Fe-rich particles [128,138]. (**A**,**B**) Intragrannular precipitated Mg_2_Si plates in two perpendicular alignments; (**C**) Mg_2_Si precipitate on the grain boundary; (**D**) TEM-BF image of rod-shaped precipitate from LTR during cooling with 0.16 K/s to 225 °C (β’/B’, [118,128]). Nucleation seems to take place on an Mn-rich dispersoid particle [128].

Figure 39 shows detailed images of quench-induced β-Mg_2_Si particles. If precipitated inside a grain, square plate particles with a thickness of about one third of their edge length (i.e., an aspect ratio of 1/3) are common. This can be seen from Figure 39A,B, as the particle in Figure 39A is in a perpendicular plane compared to that of Figure 39B. Stepwise polishing of a metallographic sample revealed this aspect ratio for several particles [138]. For alloys with small grain size close to the size of potential quench-induced β-Mg_2_Si precipitates, the precipitation of β-Mg_2_Si seems to occur only at grain boundaries [118]. The β-Mg_2_Si particles at grain boundaries have irregular and variable particle shapes (Figure 39C). At either location, the nucleation of quench-induced β-Mg_2_Si particles appears to occur on coarse (µm range) primary precipitates typically enriched in Fe, Si and Mn (see Figure 39A–C and [118,128,138]). As exemplified by Figure 39D, nucleation of the LTR B’-phase particles takes place on dispersoids (see also [17,118,173,180]).

**Figure 40:**
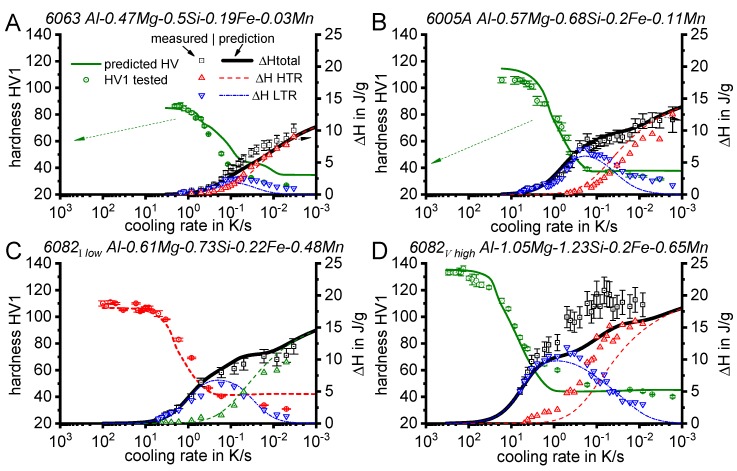
Comparison of measured and predicted values for the specific precipitation enthalpies and hardness after artificial ageing as a function of cooling rate for four AlMgSi alloys. The specific precipitation enthalpy values are plotted for total reactions (black squares), high-temperature reactions (red triangle, tip upwards) and low-temperature reactions (blue triangle, tip downwards). (**A**) 6063; (**B**) 6005A; (**C**) 6082_I_; and (**D**) 6082_V_ [118].

Figure 40 compares the measured and predicted values of the specific precipitation enthalpy and hardness after ageing for four commercial AlMgSi wrought alloys. The prediction results are drawn from the model derived in Ref. [118]. The basic ideas of the model setup are introduced in Chapter 0 below. As already seen for 6005A in Figure 30B, the specific precipitation enthalpy generally drops to zero at a certain cooling rate. For higher cooling rates, the hardness typically reaches a saturation level. In general, these model predictions are accurate, and a more detailed discussion is provided in [118]. The model considers precipitation of the phases β-Mg_2_Si and B’-Mg_5_Si_4_Al_2_. It is also notable that the predicted values for these separate phases show close agreement with the measured enthalpies of the HTR and LTR. This is particularly true for the LTR-quench-induced precipitates of B’-Mg_5_Si_4_Al_2_ and industrially relevant cooling rates.

**Figure 41:**
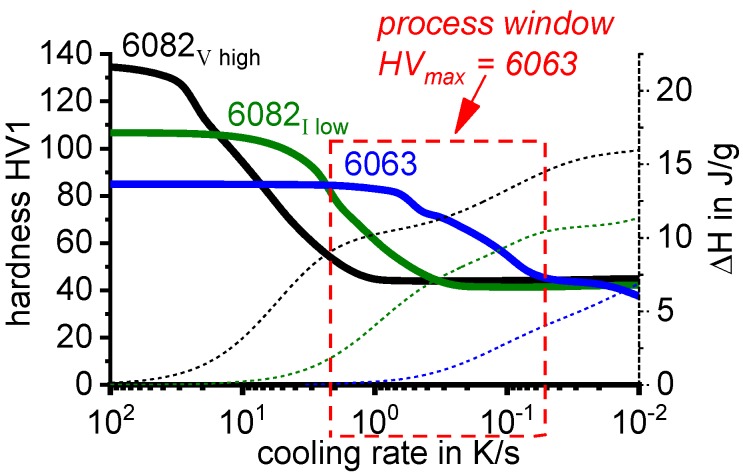
Comparison of hardness after ageing and total precipitation enthalpy predicted by the model from Ref. [118] for alloys 6063, 6082_I_ and 6082_V_.

Figure 41 shows a comparison of the predicted hardness values as a function of cooling rate for 6063 and two variants of 6082. This comparison is especially remarkable in terms of the technological application of age-hardening and the design of the applied heat treatment processes. It can be seen that the highest maximum hardness is achieved for the alloy with the highest alloying element concentration (6082_V_) after very rapid quenching. Thus, if the main objective is to obtain maximum hardness and strength, a highly concentrated alloy is required, although it is also necessary to ensure sufficiently fast cooling via the technologically applied cooling process. It may be difficult, if not impossible, to achieve >100 K/s as an average cooling rate in the relevant temperature range (≈500–200 °C) for thick sections and large batches/loads. In such conditions, it might be more appropriate to choose a leaner alloy. As can be seen from Figure 41, in a certain range of slower cooling rates, the maximum hardness is achieved with the leanest alloy in this comparison. Slower cooling rates may be of interest, for instance due to requirements for distortion control or reduction. The results obtained in this work allow us to choose either a suitable process for a specific alloy or a suitable alloy for a certain fixed process.

In Ref. [148], it was shown for 6082_III_ that β-Mg_2_Si particles, which are not fully dissolved during (insufficient) solution treatment, strongly influence the mechanism of nucleation and growth of quench-induced precipitates. Partly dissolved β-Mg_2_Si particles may occur for instance when a solution temperature below the solvus is chosen, or a solution temperature very close to the solvus temperature with short soaking durations. In the presence of undissolved β-Mg_2_Si at the end of the solution treatment, no additional nucleation is required with the onset of cooling, and these undissolved β-Mg_2_Si particles instantaneously start to grow. This effect considerably increases the quench sensitivity of the alloy. In this way, the critical cooling rate can be increased by a factor of three, and the hardness after ageing can be reduced by about 30 % for medium cooling rates [148]. As an additional effect in such cases, the HTR dominates over the entire cooling rate range (Figure A1H).

**Figure 42:**
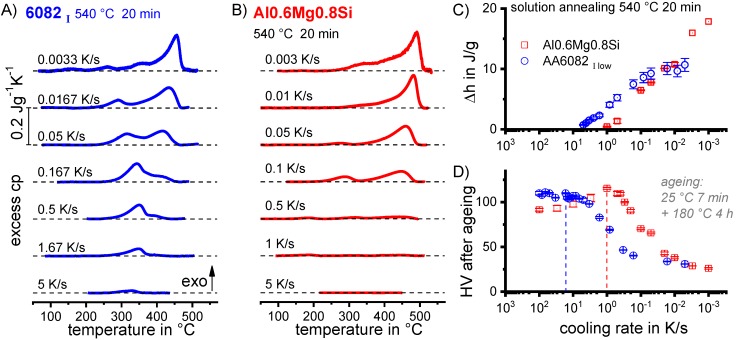
DSC cooling curves for (**A**) 6082 I and (**B**) Al0.6Mg0.8Si; (**C**) total specific precipitation enthalpies; and (**D**) hardness after ageing of both alloys as functions of cooling rate.

Furthermore, the alloying elements Fe and Mn are of primary importance for the nucleation of quench-induced precipitation, as these elements are typically found in either coarse primary particles, which act as nucleation sites for HT-Mg_2_Si particles, or in dispersoids, which are the preferred nucleation sites of the rod-shaped LT particles [17,23,48,170,180,183]. Nevertheless, the influence of these minor element additions has not yet been fully quantified. One attempt to quantify the influence of dispersoid forming elements is presented in Figure 42, which shows a comparison of the commercially produced 6082_I_ and a pure ternary Al0.6Mg0.8Si alloy. Both alloys have a highly similar content of the main alloying elements, Mg and Si. The major difference between them is the additional mass fraction of 0.2% Fe and 0.5% Mn in 6082_I_ (atomic fractions: 0.11% Fe; 0.24% Mn). Figure 42A,B show the DSC cooling curves of both alloys, while Figure 42C,D compare the specific precipitation enthalpy after cooling and the hardness after additional ageing, respectively.

**Figure 43:**
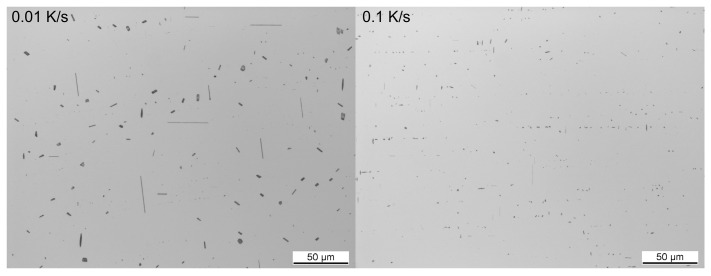
OM images of Al0.6Mg0.8Si after cooling at 0.01 K/s and 0.1 K/s.

In Al0.6Mg0.8Si, the HTRs are dominant over the entire range of cooling rates, and it can be concluded that the equilibrium phase β-Mg_2_Si is primarily precipitated. However, the nucleation mechanism of β-Mg_2_Si in the pure ternary Al0.6Mg0.8Si remains unclear. Compared to the commercial variant, substantially reduced LTRs are seen for the pure alloy, and this is probably due to the reduced number of nucleation sites. A direct comparison at about 0.1 K/s shows that for the commercial 6082_I_, quench-induced precipitation is clearly dominated by the LTR, while quench-induced precipitation for Al0.6Mg0.8Si is dominated by the HTR. For cooling rates higher than 0.5 K/s in Al0.6Mg0.8Si, barely any reaction can be seen in DSC, whereas for 6082_I_ the LTRs are still well above the DSC detection limit and the total specific precipitation enthalpy still amounts to about 5 J/g for 0.5 K/s (see Figure 42C). Although both alloys can achieve the same maximum hardness, this maximum is reached at substantially different cooling rates. A comparison of Figure 42C,D shows that the UCCRs of these two alloys differ by a factor of ≈20, which quantifies the influence of an addition of an atomic fraction of ≈0.35% Fe+Mn to a medium AlMgSi alloy. This factor may of course may also depend in detail on the particular distribution size of the dispersoid particles and therefore on the homogenisation treatment [48]. It is also interesting to note from Figure 42 that the solvus temperature of these two alloys differs. For cooling at a rate of 0.003 K/s, the onset of quench-induced precipitation for Al0.6Mg0.8Si is about 510 °C, while for 6082_I_ it is about 475 °C. It can be assumed that Si is partially bound in the eutectic primary phases and dispersoids, thus reducing the Si solute concentration at the end of the solution treatment and thereby the solvus temperature. This assumption is supported by the total specific precipitation enthalpy, for which Δh_Al0.6Mg0.8Si_ > Δh _6082I_ holds during very slow cooling. The abovementioned differences in the nucleation of the equilibrium β-Mg_2_Si phase (commercial alloy: coarse primary particles enriched in Fe, Mn, Si; lab alloys: unclear) suggest that the HTR-quench-induced precipitates nucleate more easily in commercial alloys. However, the higher precipitation onset temperature for Al0.6Mg0.8Si hints at the dominating influence of a higher amount of solved alloying elements, particularly Si, on precipitation onset in the stable phases. The opposite behaviour is seen for the LTR, which is obviously much more strongly influenced by nucleation promoted in the presence of dispersoids. In other words, in the pure ternary alloy, quench-induced precipitation is clearly dominated by the precipitation of β-Mg_2_Si, which precipitates as low aspect ratio particles at high temperatures and slow cooling rates (see Figure 43). However, β-Mg_2_Si particles are also found as high aspect ratio particles, which probably form during the second stage of the HTRs. As can be seen from Figure 42B, the HTR peak during slow cooling has a shoulder at about 400 °C. This could indicate the separation of the precipitation regimes for these particle morphology types. A comparison of the OM image of the sample cooled at 0.01 K/s in Figure 43 with the DSC cooling curve at the same rate in Figure 42 indicates that both particle shapes are present after completion of the HTRs, as no LTRs occur during cooling at 0.01 K/s. The high aspect ratio particles identified after cooling at 0.01 K/s reach lengths of up to ≈50 µm.

**Figure 44:**
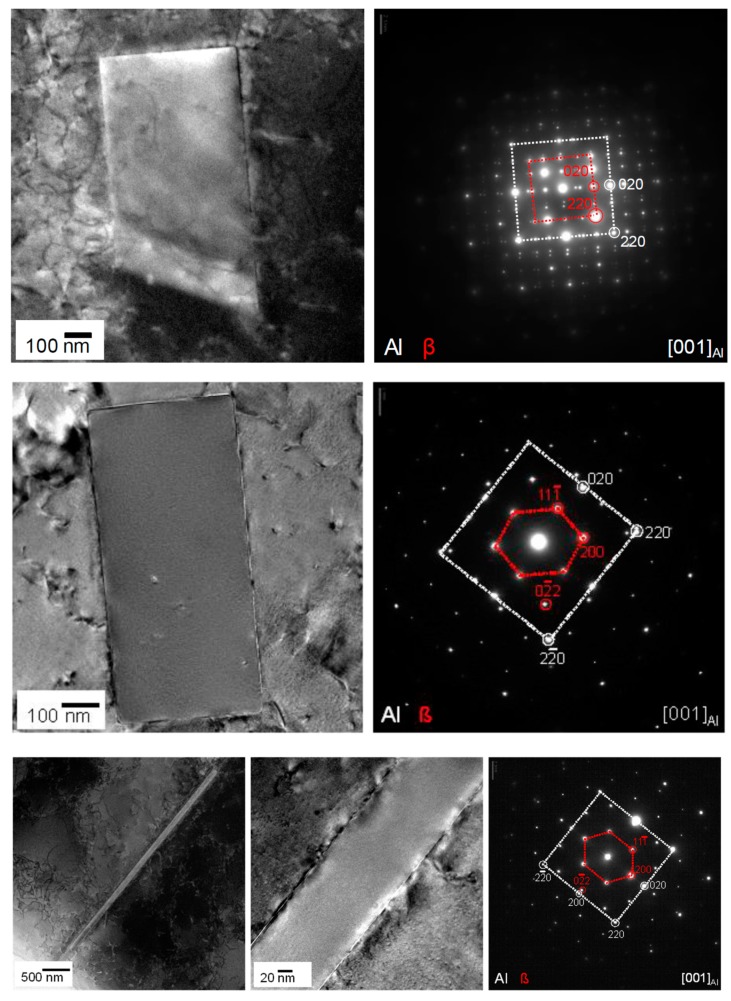
Three different fcc β-Mg_2_Si phase particles in Al0.8Mg0.6Si after cooling at 0.1 K/s. The particles have different orientation relationships with the matrix (TEM work by Shravan Kairy and Matthew Weyland, Monash University, Melbourne, Australia).

**Figure 45:**
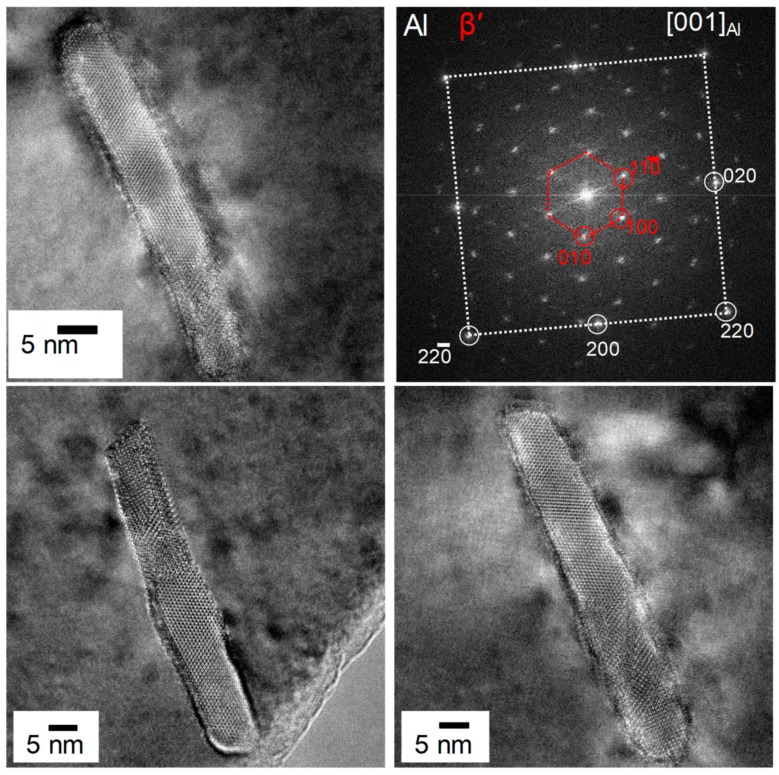
Fine quench-induced hexagonal β′-Mg_9_Si_5_ particles after cooling Al0.6Mg0.8Si at 0.1 K/s. The β′-particles apparently contain disordered areas (TEM work by Shravan Kairy and Matthew Weyland, Monash University, Melbourne, Australia).

In general, most of the quench-induced precipitates found in the pure ternary alloys Al0.6Mg0.8Si and Al0.8Mg0.6Si after cooling with 0.1 K/s are β-Mg_2_Si [132]. This holds not only for relatively coarse, low aspect ratio particles (Figure 43, originating from HTR) but also for relatively fine/small particles (Figure 44). The origin of the latter is not clearly determined, but their size hints at the potential additional precipitation of Mg_2_Si during LTR in these laboratory alloys. However, during cooling at 0.1 K/s in the ternary laboratory alloys, two further types of MgSi precursor phases were identified, namely hexagonal β’-Mg9Si5 (Figure 45) and U1-MgAl2Si2. From Figure 45, it is particularly interesting to note that these fine particles are polycrystalline, and are likely to contain disordered areas such as those previously reported for fine precipitates formed during ageing in AlMgSi alloys [184,185,186,187].

Another interesting aspect can be seen in Figure 42D, in which the hardness after cooling and ageing for pure Al0.6Mg0.8Si shows a maximum at the UCCR determined by DSC, and unlike all other alloys studied in this work, the hardness drops with faster cooling rates. Similar results have been reported for high-purity AlZnMg alloys [188]. It is possible that small quench-induced precipitates forming around the UCCR contribute a hardening effect.

The information revealed on the nature of quench-induced precipitates for AlMgSi alloys is summarised in Table 1.

**Figure 46:**
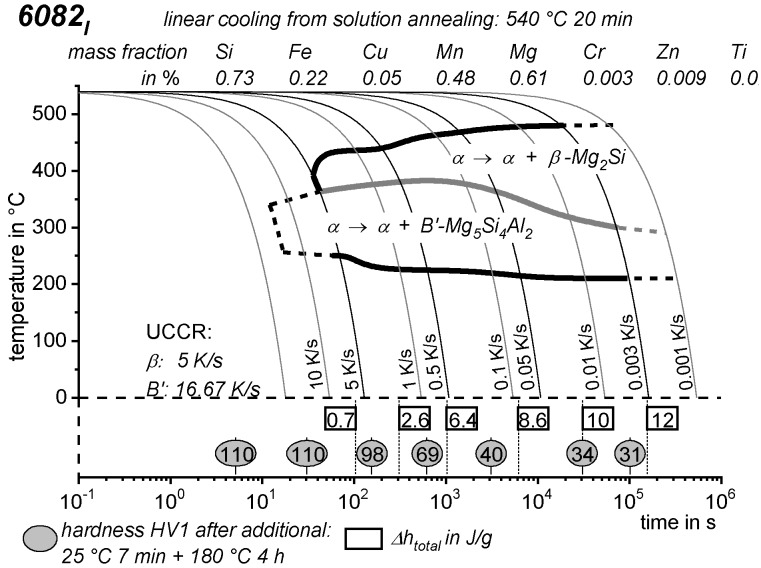
CCP diagram for 6082_I_ [128,146].

**Figure 47:**
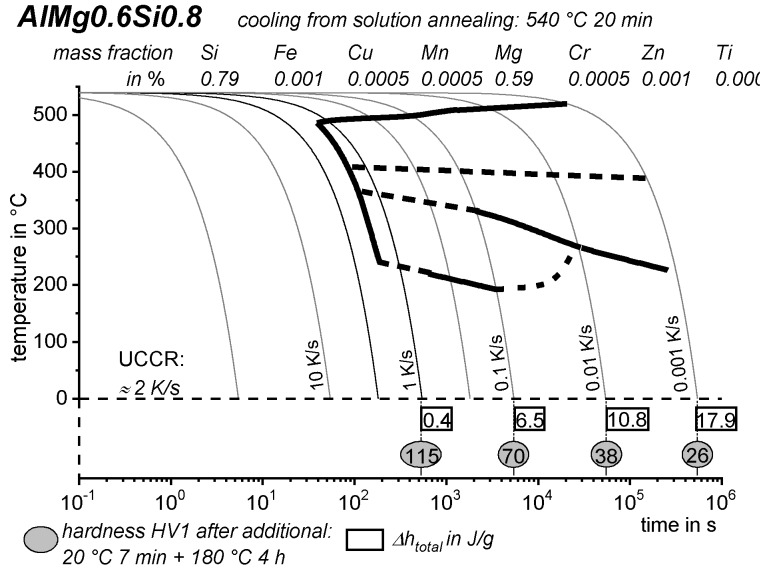
CCP diagram for Al0.6Mg0.8Si.

The CCP diagrams for 6082_I_ and Al0.6Mg0.8Si are compared in Figure 46 and Figure 47, respectively. Here, we can see a considerable difference between these alloys, which is caused by the different numbers of nucleation sites and thus different nucleation mechanisms for quench-induced precipitates. A comparison with a CCP diagram for a 6082 alloy obtained from in situ electrical resistivity curves is possible by considering Ref. [85]. In Ref. [85], over a cooling rate range of ≈16–0.03 K/s, the precipitation start temperature was found to be about 375 °C, which is close to the start of the LTR determined by DSC (compare Figure 46). In Ref. [85], the end-temperature of precipitation was evaluated to be about 225–300 °C, which is comparable to the end of the LTR identified by DSC. From this, it can be concluded that the in situ electrical resistivity measurements were able to detect the LTR in 6082. It seems likely that the HTRs were not identified by this method; however, the UCCR was determined within a range of ≈16 to 30 K/s [85], which is very similar to that identified for 6082_I_ [128].

#### 3.2.3. 7xxx AlZnMg(Cu) Wrought Alloys

**Figure 48:**
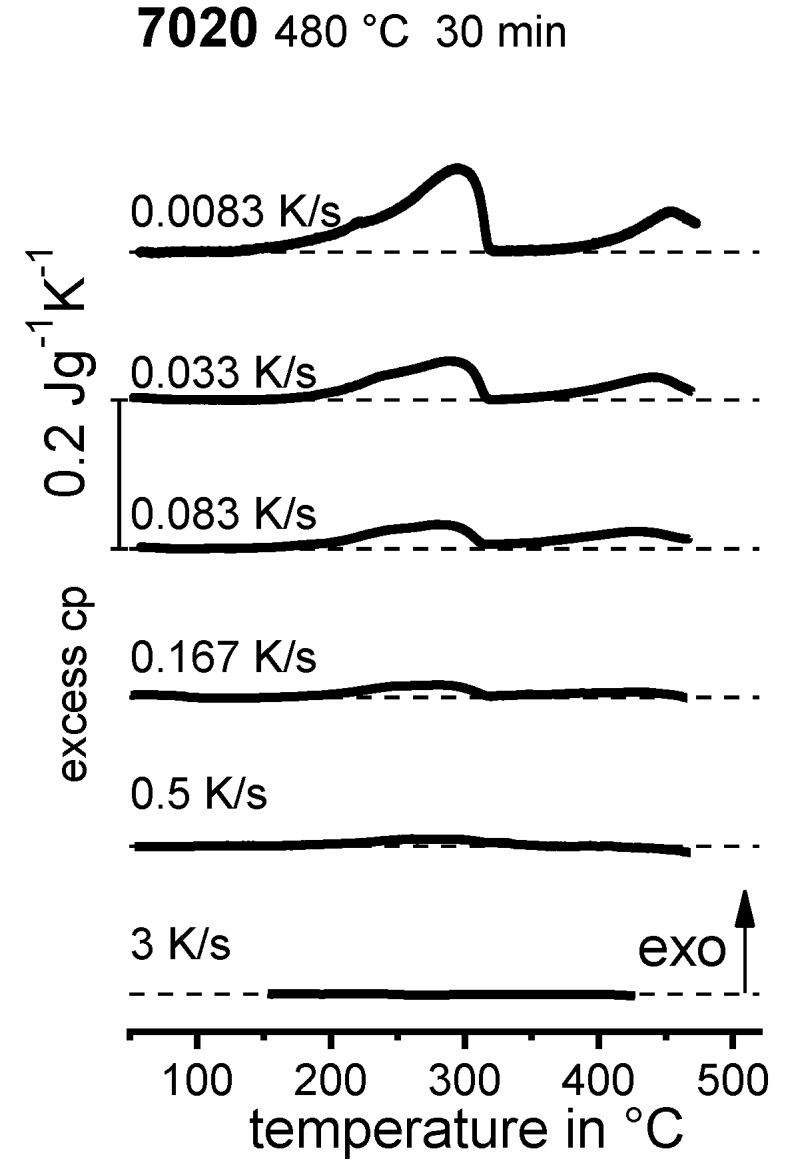
Cooling DSC curves for 7020.

**Figure 49:**
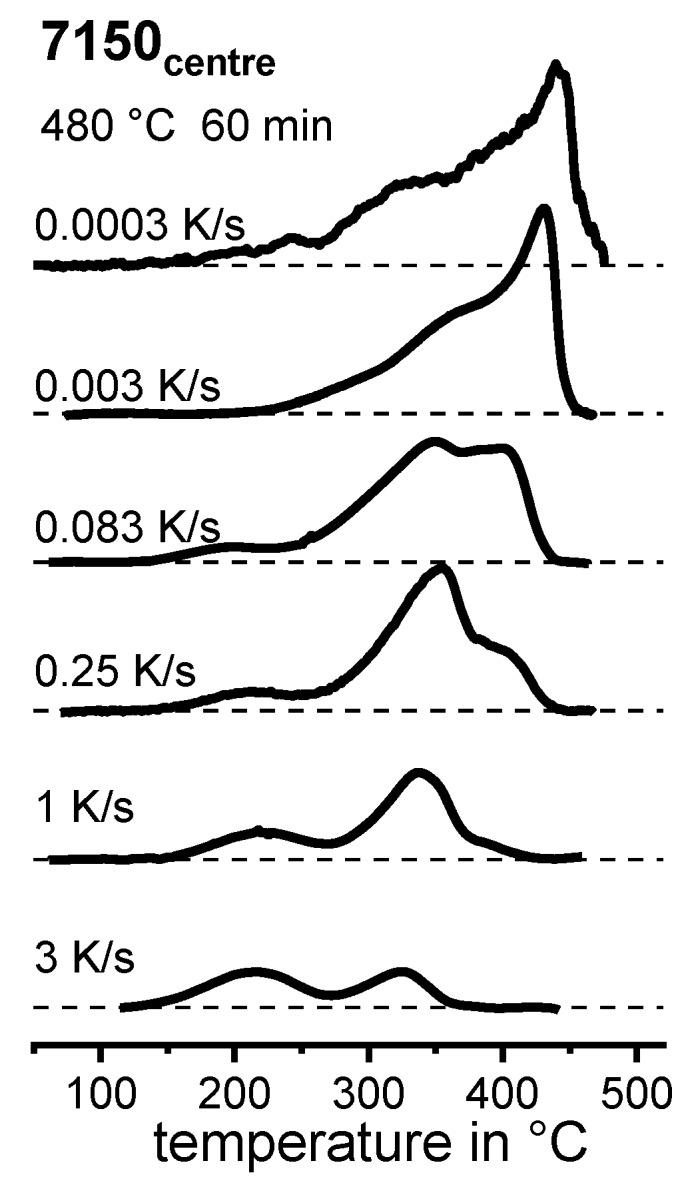
Cooling DSC curves for 7150.

**Figure 50:**
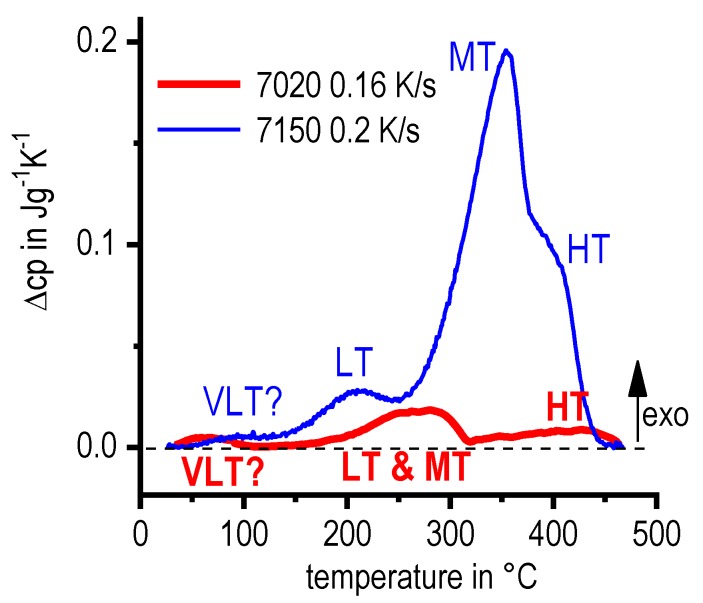
Cooling DSC curves for 7150 and 7020, showing hints of a precipitation reaction at very low temperatures (150 to 50 °C) [133].

**Figure A3:**
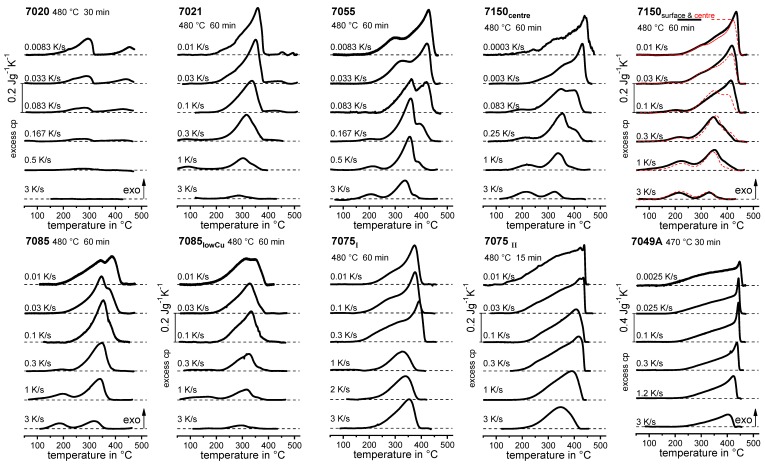
Comparison of DSC cooling curves for 10 different AlZnMg(Cu) alloys. The ordinate scaling is identical for all alloys except 7049A, for which the distance of the shifted zero-levels is doubled.

Ten variants of the alloying system AlZnMg(Cu) are considered in this work. Due to the multiple potential combinations of alloying elements, the 7xxx alloying system can be considered a very complex system in terms of precipitation. It is often a quarternary system, as in addition to Al, Zn and Mg, Cu and other elements such as Si are often incorporated. Cu and Si may add additional precipitation sequences, for instance those of S-Al_2_CuMg and/or β-Mg_2_Si [35,189]. Moreover, the Zn- and Mg-containing phases do not have a fixed composition, and isostructure variants are known for the η-Mg(Zn,Al,Cu)_2_ phase [190,191]. For most of the 10 AlZnMg(Cu) alloys analysed here, DSC indicates the presence of three or four different main reaction intervals during cooling. DSC cooling curves for 7020 (an AlZnMgSi alloy) and 7150 (an AlZnMgCu alloy) can be seen in Figure 48 and Figure 49, respectively. Although these two alloys have substantially different alloy compositions, they both show at least three main reaction intervals, which are HTRs (typically ≈450–350 °C), medium-temperature-reactions (MTRs, ≈350–250 °C) and LTRs, (≈250–150 °C). This holds for the majority of the 10 AlZnMg(Cu) alloys investigated, as can be seen from Figure A3 in the Appendix A. In some cases, a fourth reaction was detected at an even lower temperature of about 150–50 °C, as shown in Figure 50. For the 7xxx alloys with highest concentration of alloying elements such as 7075, 7049A (Figure A3 in the Appendix A and Refs. [161,162]) and 7068 [192], cooling DSC appears to show only one very broad peak, which ranges from about 450 to 150 °C. It can be assumed that this very broad heat effect is caused by the sum of numerous reactions.

The in situ cooling DSC experiments shown in Figure 49 cover four orders of magnitude and thereby an extreme dynamic DSC. The slowest cooling at 0.0003 K/s requires a cooling duration of >17 days, while the fastest cooling 3 K/s is completed within 2.5 min. Furthermore, DFSC was performed for this alloy, extending the investigated scanning rate range to some hundreds of K/s. Thus, the fastest cooling was achieved in times of less than 1 s.

Similarly to the AlSi and AlMgSi alloys, the reactions are in total increasingly suppressed with increasing cooling rates. Thus, in most cases, the reactions at higher temperatures are first suppressed, while the reactions at lower temperatures first show an increase in the fraction transformed. The latter is due to the increased concentration of alloying elements left in solution after the suppressed HTR is complete.

**Figure 51:**
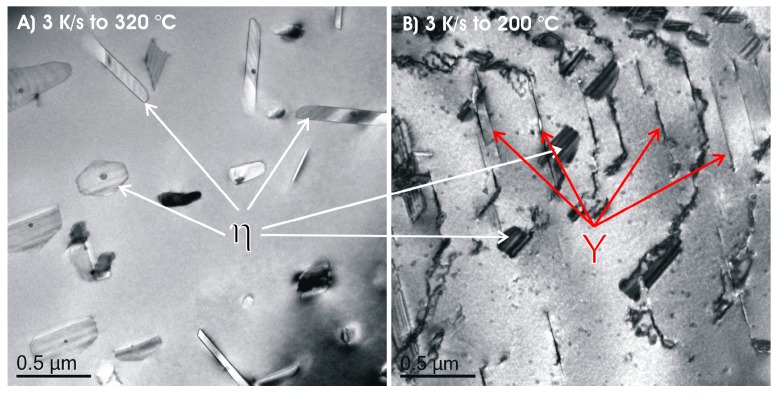
Quench-induced precipitation in 7150 during cooling at 3 K/s [129].

The type and nature of quench-induced precipitation in 7xxx alloys has been widely investigated [31,35,103,104,133,143,189,193,194,195,196]. In general, it can be stated that in presence of Cu, the S-Al_2_CuMg phase is precipitated, and in the presence of Si, the β-Mg_2_Si phase is precipitated during the HTRs [119,133,189,197]. Nucleation of these two phases appears to take place on coarse intermetallic particles. In almost every AlZnMg alloy, the MTRs seem to be dominated by the precipitation of isostructure variants of the η-Mg(Zn,Al,Cu)_2_ phase [119]. Examples of this quench-induced phase are seen in Figure 51. Nucleation of quench-induced η-Mg(Zn,Al,Cu)_2_ apparently occurs on dispersoids [31,35,119,189,194,195,196]. In cold-rolled sheet material, dispersoids are heterogeneously aligned in lines, resulting in bands of quenched-induced precipitates [196]. As a result, fine precipitates originating from ageing can only be found heterogeneously distributed between the bands of quench-induced precipitates [195].

In AlZnMg(Cu) alloys, quench-induced precipitates of the η-Mg(Zn,Al,Cu)_2_ phase can be considered the most detrimental precipitation in terms of quench sensitivity, since this phase forms at relatively fast cooling rates while its precipitates barely have a hardening effect. Quench-induced phases at even lower temperatures, namely thin Y-phase platelets [119,143,198] and clusters [102,103,104], may form during even faster cooling. These quench-induced precipitates are small enough to cause a significant hardening effect [143].

**Figure 52:**
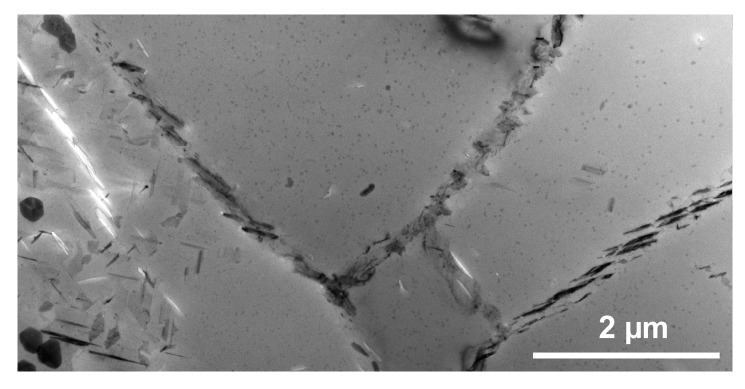
TEM image of an air-cooled 7150 sample (average cooling rate 1 K/s) showing a recrystallised grain on the left-hand side and several subgrains on the right. It can be seen that the quench-induced η-Mg(Zn,Al,Cu)_2_ precipitates preferentially nucleate at grain/subgrain boundaries and also appear inside the recrystallised grain, nucleating at Al_3_Zr dispersoids [119].

As outlined in several prior works [31,35,86,189,194,199,200,201,202,203,204], nucleation of quench-induced η-Mg(Zn,Al,Cu)_2_ takes place on dispersoids. At least for Al_3_Zr dispersoids, it has also been shown that their nucleation activity for quench-induced η-Mg(Zn,Al,Cu)_2_ is dependent on the interface relation of the dispersoid to the matrix [189,194], and only incoherent Al_3_Zr dispersoid particles appear to be active nucleation sites for η-Mg(Zn,Al,Cu)_2_. Al_3_Zr dispersoids are typically coherent with the Al matrix, although after thermo-mechanical processing, recrystallisation can occur. This recrystallisation in the Al matrix can change the interface of the dispersoid towards incoherence. An example of this phenomenon is seen in Figure 52, which shows a TEM image of hot-rolled 7150 [189,194]. Subgrains and recrystallised grains were distinguished by the lattice orientation, as revealed by SEM-EBSD (see Refs. [189,194] for details). Quench-induced η-Mg(Zn,Al,Cu)_2_ particles are found at subgrain-boundaries and in the recrystallised grain shown on the left, nucleated on incoherent Al_3_Zr dispersoids. However, in the non-recrystallised subgrains, the coherent Al_3_Zr dispersoids do not have quench-induced precipitates attached.

**Figure 53:**
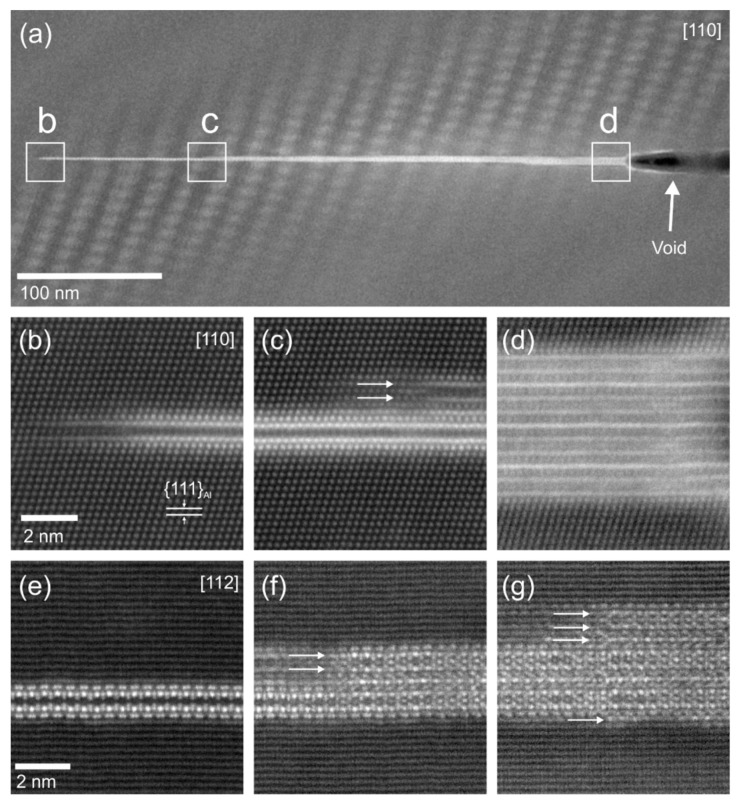
HAADF-STEM images of Y-phase platelets enriched in Zn and Cu after cooling of 7150 at 10 K/s, viewed from the [110]α direction, showing that the thickness varies along its length, with growth ledges indicated by arrows in (**c**). The regular pattern in the matrix on either side of the plate in (**a**) is an artefact caused by Moiré fringing between the lattice and scan frame. This plate appears to be nucleating from an attached void. Similar images in (**e**–**g**) from a second plate, but viewed from the [112]α direction, show variations in thickness and stacking order along the length of the precipitate [143].

The LTR in 7150 has been shown to relate to a previously unknown thin plate phase that is enriched in Zn and Cu, and it was labelled as the Y-phase [143]. Y-phase platelets have been confirmed to form during cooling by others too [198]. An example of a Y-phase platelet can be seen in the high angle annular dark field (HAADF)-STEM images in Figure 53. Based on the interrupted quenching method, we conclude that the LTR in a temperature range of about 250–150 °C is dominated by the precipitation of the Zn–Cu-rich thin plate Y-phase (compare Figure 51 with the DSC curve of 3 K/s in Figure 49). The Y-phase features strong structural similarities to the T1 phase in Al-Li alloys, with a hexagonal symmetry (a = 0.429 nm, c = 1.385 nm). The authors of Ref. [88] assume the low-temperature precipitation to refer to the η’-phase.

**Figure 54:**
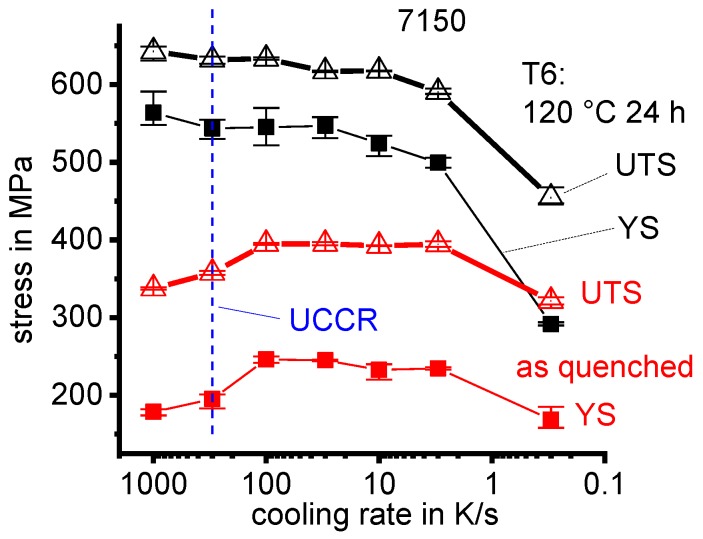
Ultimate tensile strength and yield strength of 7150 in the as-quenched and artificially aged conditions [143].

At even lower temperatures of about 150 to 50 °C (see VLT peak in the DSC curves in Figure 50), co-clusters may precipitate during quenching, as shown in SAXS experiments involving continuous cooling of 7449 to below 100 °C in Refs. [103,104]. The small Y-phase platelets and clusters cause a direct hardening effect, which is seen in the peaks in the as-quenched hardness, yield strength and ultimate tensile strength for cooling rates of about one to two orders of magnitude slower than the UCCR [143]. Tensile testing indicates that this Y-phase appears to contribute up to ≈50 MPa to the as-quenched strength in the investigated alloy 7150 (see Figure 54, [143]). The findings on quench-induced precipitation in AlZnMg(Cu) alloys are summarised in Table 2.

**Figure 55:**
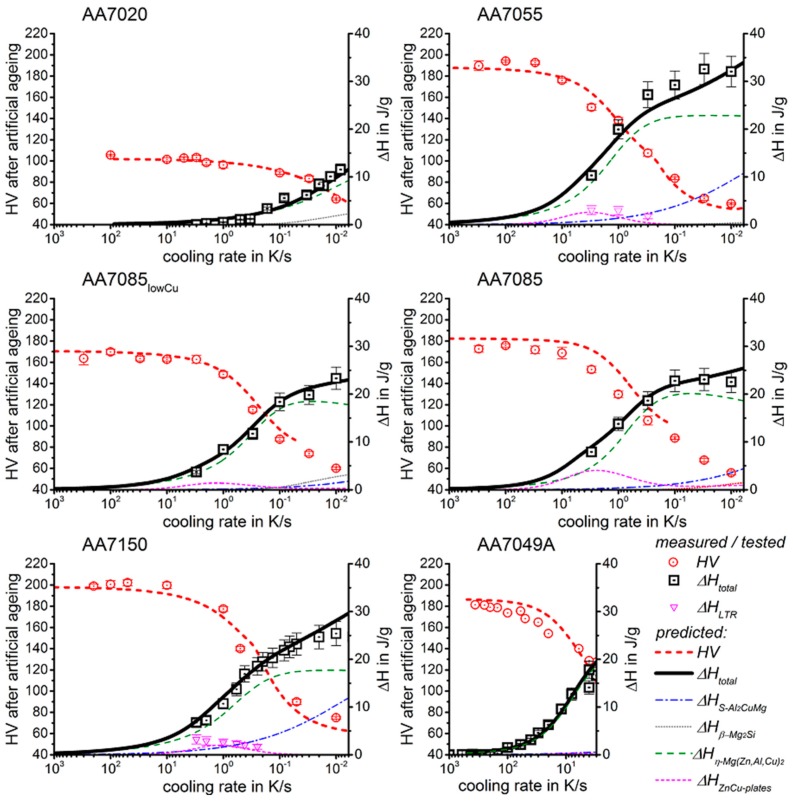
Measured values and model predictions for hardness after ageing and specific precipitation enthalpies of six AlZnMg(Cu) alloys [119]. Values for the total specific precipitation enthalpy were obtained by in situ cooling DSC as outlined above. For 7049A, chip-sensor based differential fast scanning calorimetry was applied [130,162].

**Figure A4:**
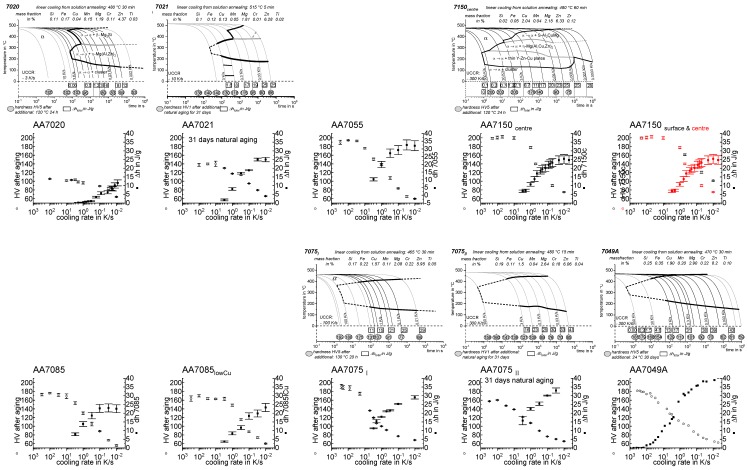
Comparison of the continuous cooling precipitation diagrams, the total specific precipitation enthalpies after cooling and hardness after additional artificial ageing, for 10 different AlZnMg(Cu) alloys.

In Figure 55, the experimentally obtained values for the total specific precipitation enthalpy after cooling and the hardness after artificial ageing of six different AlZnMg(Cu) alloys are compared to the predictions of the model derived in Ref. [119]. A general chart of these values as a function of cooling rate for each 7xxx Al alloy investigated is given in the Appendix A, in Figure A4. As can be seen from Figure 55, the model predictions are found to be very accurate to a wide range of AlZnMg(Cu) alloys and the total specific precipitation enthalpy generally decreases with increasing cooling rate for all alloys. A high level of similarity to the AlMgSi alloys is found for the general kinetic behaviour. However, the specific enthalpy values, as well as the obtainable hardness values, are generally higher for the 7xxx alloys. This is reasonable, as the latter contain higher concentrations of alloying elements. As for the 6xxx alloys, the hardness after ageing reaches a saturation level for cooling rates above the UCCR ranging from about 3 K/s for 7020 to about 300 K/s for highly concentrated alloys such as 7150 or 7049A.

**Figure 56:**
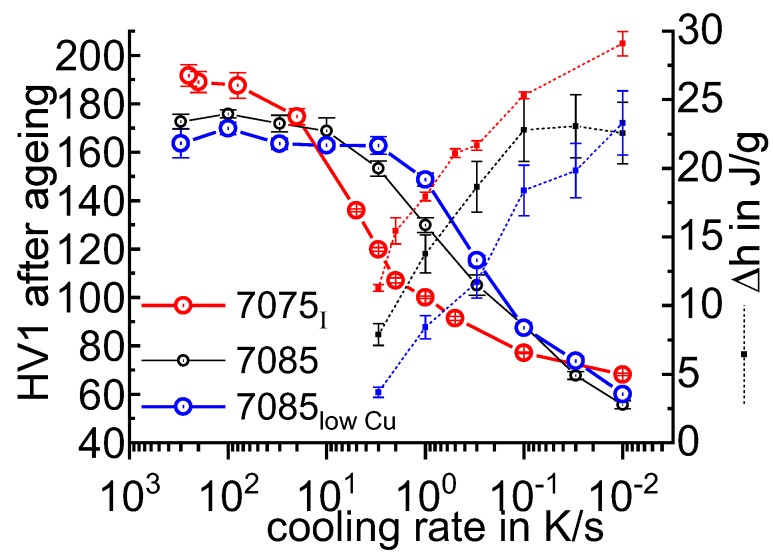
Comparison of experimentally obtained specific precipitation enthalpies after cooling and hardness after additional ageing for three differently concentrated AlZnMgCu alloys.

As discussed for AlMgSi alloys, it also holds for AlZnMgCu alloys that a lower concentrated AlZnMgCu alloy can achieve higher hardness values if cooling is restricted to medium cooling rates. Figure 56 compares 7075I, 7085 and 7085lowCu. Of these three variants, 7085lowCu, has the lowest Mg content, despite its low Cu content. The age-hardening potential of 7085lowCu that remains after cooling at 1 K/s results in a hardness of almost 150 HV1. At this cooling rate, the age-hardening potential of 7075I has already dropped to just 100 HV1. Under these conditions, the lower concentrated alloy would achieve ≈150 % of the strength of the highly concentrated alloy (i.e., 7085lowCu is less quench sensitive than both 7075I and 7085). Considering the concentrations of alloying elements, it can be assumed that the concentration of Mg predominantly drives the precipitation kinetics. This fits with the finding in [119] that the main quench-induced precipitate phase that causes quench sensitivity is η-Mg(Zn,Cu,Al)_2_ for these types of alloys.

In general, it is found that the AlZnMgCu series should be considered very quench sensitive. This is reasonable, as these alloys typically contain the highest concentration of alloying elements amongst the age-hardening Al alloys. However, there are indications that small additions of Ge and Ag might reduce the quench sensitivity of 7xxx alloys [205].

**Figure 57:**
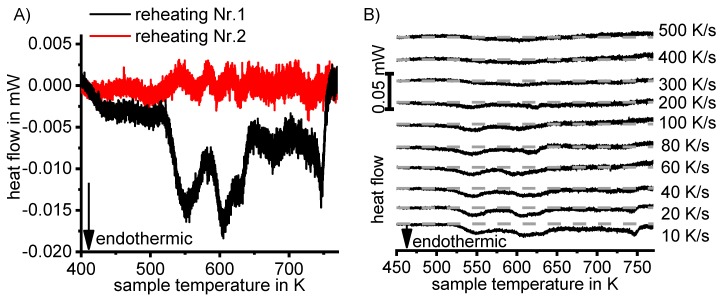
(**A**) Raw 1000 K/s reheating curves for states previously cooled at rates of 10 K/s (first reheating) and 10^5^ K/s (second reheating = baseline measurement). (**B**) Subtracted measurement curves, i.e., curves measured for the first reheating minus the curves measured for the second reheating, for various cooling rates. The baselines for integration are indicated by dashed lines [129].

**Figure 58:**
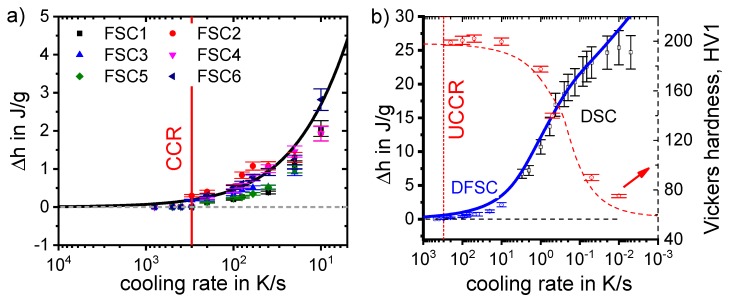
(**A**) Specific precipitation enthalpy after cooling from solution annealing of alloy 7150, as a function of cooling rate measured by DFSC. (**B**) Specific precipitation enthalpy after cooling from solution annealing and Vickers hardness after subsequent ageing (120 °C 24 h) of alloy 7150, as a function of cooling rate. The enthalpy values obtained by DFSC are shown as average values and standard deviation for six samples. The solid lines are model predictions from Ref. [119]. The DSC and hardness data tested on large samples were published in Ref. [133]. Hardness was tested for samples at the millimetre scale at the same cooling rates, obtained using a quenching dilatometer [129].

From Figure 55 and Figure 56, it also becomes obvious that in several cases, a substantial amount of enthalpy change caused by quench-induced precipitates is detected. Figure 55 includes experimental findings from DFSC obtained on 7049A in a range of cooling rates up to some hundreds of K/s [130,162]. As outlined in Section 3.1.3, DFSC allows us to obtain the specific precipitation enthalpies of highly concentrated alloys via a differential reheating measurement [129,130,162]. This is demonstrated in Figure 57A, which shows raw DFSC data for the first reheating (after relatively “slow” cooling with 10 K/s) and a second reheating after overcritical fast cooling (100,000 K/s, baseline). During the first reheating at 1000 K/s, endothermic dissolution reactions of phases precipitated during the preceding “slow” cooling are clearly seen. Figure 57B shows DFSC data for cooling rates of up to 500 K/s. Normalisation by sample mass and scanning rate and subsequent integration of the curves, as shown in Figure 57B, allows us to obtain the specific precipitation enthalpies as a function of cooling rate [129]. Figure 58A shows precipitation enthalpies for six samples of 7150 [129]. In Figure 58B, the averaged DFSC values from [129] and data from DSC [133] are plotted, and these fit together smoothly. The UCCR of 7150 was determined to be about 300 K/s for 7150 [129]. In Figure 58, the solid lines for the specific precipitation enthalpy Δh and for the hardness after ageing are the model predictions from [119]. The model predictions were created without knowledge of the DFSC data, as the DFSC experiments had not yet been done. Very good agreement between the measured and predicted values is seen, and this further confirms the high accuracy of the derived model for quench-induced precipitation of AlZnMg(Cu) alloys.

**Figure 59:**
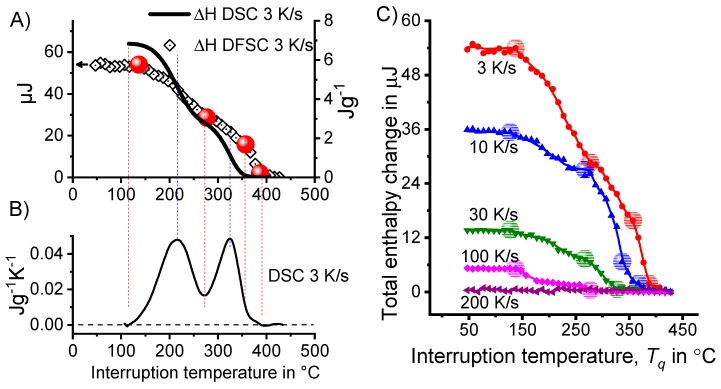
Results for 7150, showing (**A**) the total enthalpy change at a cooling rate of 3 K/s measured by DFSC and DSC; (**B**) the DSC curve at a cooling rate of 3 K/s, shown for comparison. The vertical red dashed lines indicate the precipitation start and end temperatures. (**C**) Total enthalpy change at different interruption temperatures and different cooling rates. The enlarged dots indicate the transition temperatures for the various precipitation reactions [129].

As demonstrated above, the DFSC-DRM is very helpful in assessing the kinetics of quench-induced precipitation as a function of cooling rate. However, no precipitation start and finish temperatures, which are crucial for the completion of a CCP diagram, are evaluable from the measurements shown above. In Ref. [129], the DRM was developed further to allow a temperature-dependent recording of the precipitation enthalpy values (Figure 20C). The results are summarised in Figure 59. The transition temperatures evaluated in Figure 59C allow completion of the CCP diagram with respect to the characteristic start and end temperatures of quench-induced precipitates.

**Figure 60:**
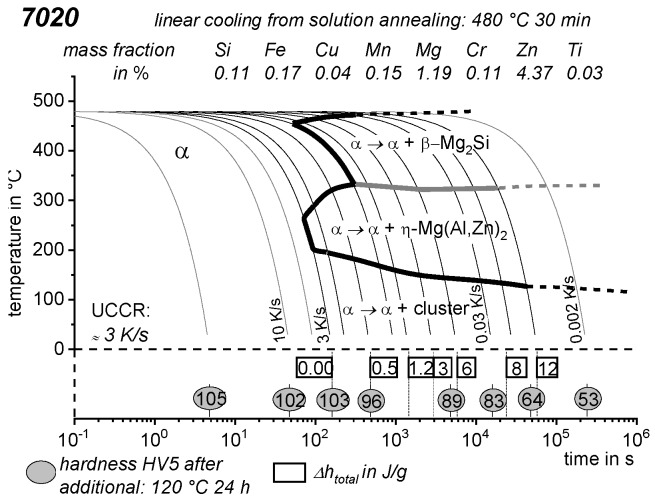
Continuous cooling precipitation diagram for 7020 [146].

**Figure 61:**
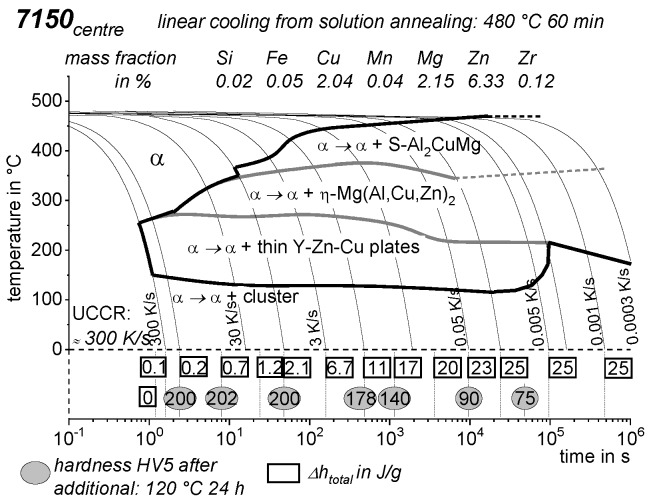
Complete continuous cooling precipitation diagram for 7150, covering seven orders of magnitude of cooling rates/cooling duration.

Figure 60 shows the CCP diagram for 7020, and this can be compared to Figure 61, which shows the complete CCP diagram for 7150. The latter covers an extreme range of seven orders of magnitude of analysed cooling rates. The CCP diagram in Figure 61 incorporates quantitative data from five different types of DSC device, as outlined in Section 2.2, and results from hardness testing over the entire cooling rate range, allowing for a profound knowledge of the kinetics quench-induced precipitation.

**Figure 62:**
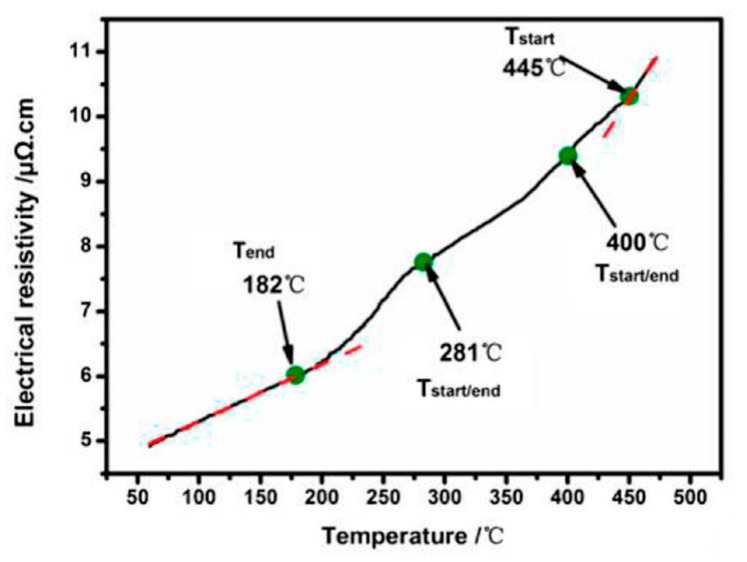
Evaluation of the characteristic transformation start and end temperatures from in situ measurements of the electrical resistivity [88]. Example of an average cooling rate of 0.7 K/s.

**Figure 63:**
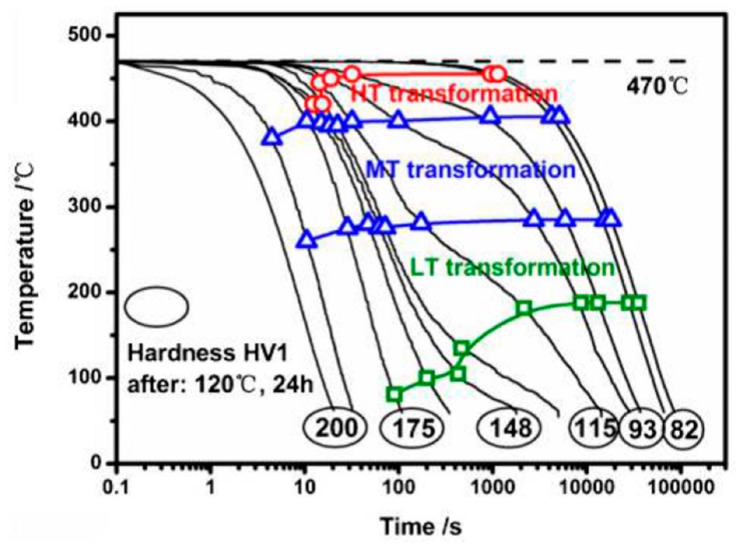
CCP diagram for 7050 obtained by in situ electrical resistivity measurements in Ref. [88]. Mass fractions of major alloying elements in %: 6.1 Zn, 2.15 Mg; 2.37 Cu.

For comparison, Figure 62 illustrates in situ electrical resistivity measurements and Figure 63 the obtained CCP diagram for alloy 7050 taken from Ref. [88]. A comparison of the evaluation in Figure 62 with the DSC cooling results seen in Figure 49 indicates that DSC generates much more convincing results, particularly in terms of reaction identification. However, highly similar results were obtained in Ref. [88] for 7050 as for 7150, which has a similar chemical composition. Using electrical resistivity measurements, the authors of Ref. [88] identified three distinct reactions at high, medium and low temperatures. The temperature ranges were relatively similar, and complete suppression of the HTR was identified within a similar range of cooling rates as in the CCP diagram for 7150 in Figure 61. The in situ electrical resistivity measurements were obtained during nonlinear cooling, which might be an advantage in terms of comparability with technological applications. The cooling time range covered is relatively large, although it is still narrower than that of the DSC work.

**Figure 64:**
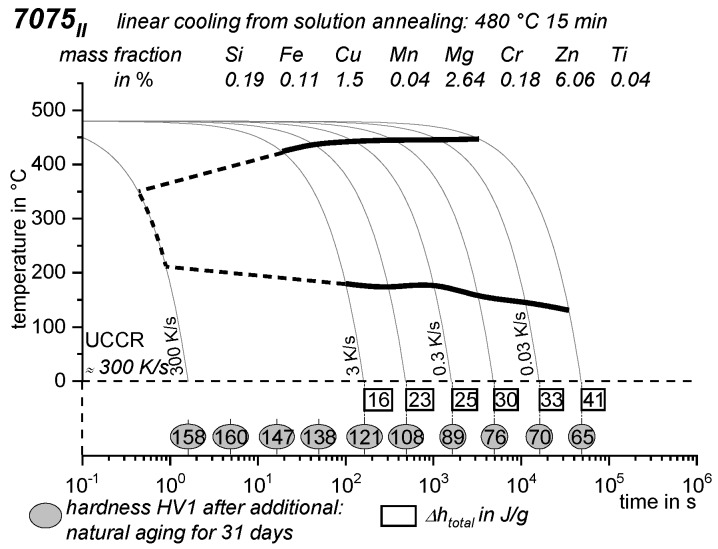
CCP diagram for 7075_II_ [161].

**Figure 65:**
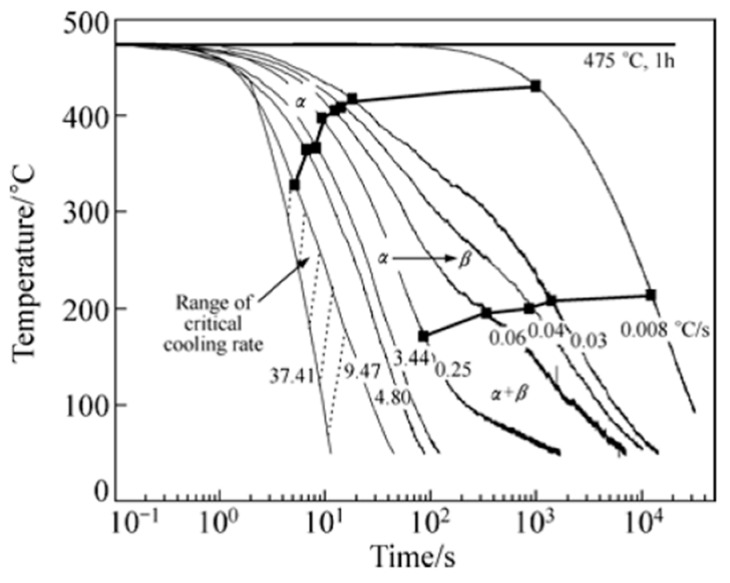
CCP diagram for alloy 7075 from Ref. [60] obtained by in situ voltage measurements. Mass fractions of major alloying elements in %: 5.44 Zn; 2.55 Mg; 1.37 Cu.

Another set of CCP diagrams for two variants of 7075 is compared in Figure 64 and Figure 65. The diagram in Figure 64 was recorded by DSC and hardness testing after natural ageing [161], while the second was established based on in situ electrical resistivity measurements in Ref. [60]. Both variants of 7075 considered here have comparable chemical compositions, although the variant from Ref. [161] is slightly more highly concentrated. For 7075, both methods obtained similar results in terms of the temperature ranges evaluated for the start and end of precipitation. However, the UCCRs are significantly different. While a combination of DSC and hardness testing after controlled linear cooling gave a rate of 300 K/s, the in situ electrical resistivity measurements suggested a UCCR range of ≈10 to 40 K/s [88]. This difference may result from a significant batch sensitivity in terms of the quench sensitivity of precipitation hardening Al alloys or from the abovementioned difficulties in evaluating in situ electrical resistivity measurements.

#### 3.2.4. 2xxx AlCu(Mg) Wrought Alloys

**Figure 66:**
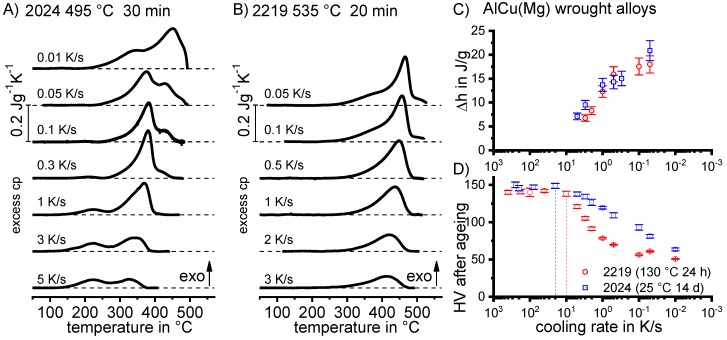
Comparison of two AlCu(Mg) alloys: (**A**) DSC cooling curves of 2024; (**B**) DSC cooling curves for 2219; (**C**) total specific precipitation enthalpies after cooling; and (**D**) hardness after additional ageing for both alloys.

The kinetic behaviour of quench-induced precipitation in AlCu(Mg) alloys is shown in Figure 66 based on DSC cooling data for 2024 and 2219. The total precipitation enthalpy (Figure 66C) and the hardness after ageing (Figure 65D, 2024 natural ageing, 2219 artificial ageing) are also shown. As seen for the other alloy systems, multiple precipitation reactions occur. During slow cooling of 2219, two major reaction areas can be detected within the considered cooling rate range, while for 2024, three distinct reaction intervals can clearly be seen. For 2219, significant superposition of the two reaction peaks occurs, meaning that the second peak is seen only as a shoulder at ≈400 °C during slow cooling. During slow cooling of 2024, only two main reaction peaks are seen, a HTR and a medium temperature reaction (MTR). The HTR is increasingly suppressed with increasing cooling rate. At the same time, the MTR increases in intensity up to a cooling rate of about 0.3 K/s. If the suppression of the HTRs is almost complete, an additional LTR (≈250–150 °C) is seen. 2219 has only Cu as its main alloying element, but 2024 contains a considerable amount of Mg in addition to Cu. This obviously adds an additional reaction to the quench-induced precipitation.

As can be seen from the specific precipitation enthalpies in Figure 66C, the total precipitation process is increasingly suppressed with rising cooling rate. The specific precipitation enthalpies of both alloys are relatively similar. For the two AlCu(Mg) alloy variants considered here, the UCCR could not be determined by in situ cooling DSC. At the fast rates measured with DSC (3 and 5 K/s, respectively), relatively high specific precipitation enthalpies of about 7 J/g are still detected. Supplemental hardness testing after various cooling rates and subsequent ageing shows hardness saturation levels at cooling rates of above 10 K/s for 2219 and 20 K/s for 2024. These rates can therefore be considered the UCCRs for these alloys for the purposes of technological applications. It is noteworthy that although the UCCRs for the two alloys are similar, the loss in hardening potential (due to quench-induced precipitates) is more severe for 2219. Hence, the achievement of high hardness values over the whole thickness of a thick plate is restricted to a reduced maximum thickness compared to 2024.

In Ref. [206], quench-induced precipitation in an AlCuMg alloy 2618 was investigated. Cooling DSC was conducted over a cooling rate range of about 0.02 to 1 K/s. The DSC results for 2618 obtained in Ref. [206] are highly similar to those for 2024 in this work, and comparable precipitation enthalpies have been determined. However, since very slow cooling was not possible in the DSC device used in [206], the authors could not identify the HTR seen in 2024 (Figure 66).

The authors of Ref. [206] ascribe precipitation in an AlCuMg alloy at high temperatures to the formation of S-Al_2_CuMg or S’-phase. In Ref. [102], the S-phase was also revealed by SAXS for cooling rates lower than 0.5 K/s. As the HTR in Figure 66A is substantially suppressed at rates higher than 0.5 K/s, it can be concluded that the HTRs revealed by DSC refer to the formation of S-Al_2_CuMg. In addition, also isothermal experiments on a 2024 type alloys suggest the formation of the S-Al_2_CuMg phase [58,207,208] at high temperatures occurring on grain boundaries [58,207] and for medium temperatures precipitation of the S-Al_2_CuMg as well as the θ-Al_2_Cu phase, both nucleating on dispersoids [58,207]. The quench-induced formation of the θ-Al_2_Cu phase in AlCuMg alloys is also supported by Refs. [209,210]. In Ref. [211], indications of quench-induced precipitation of the T-Al_20_Cu_2_Mn_3_ phase were found in a Jominy end quench sample of another AlCuMg alloy. Continuous cooling SAXS measurements in Ref. [102] revealed the precipitation of Cu-Mg co-clusters below 250 °C. This shows good agreement with the low-temperature DSC peak for 2618 and 2024.

For 2219, it seems highly likely that quench-induced precipitates will be dominated by θ-Al_2_Cu and potentially θ’-precipitates. This is supported by findings from isothermal experiments in Refs. [209,210,212,213].

**Figure 67:**
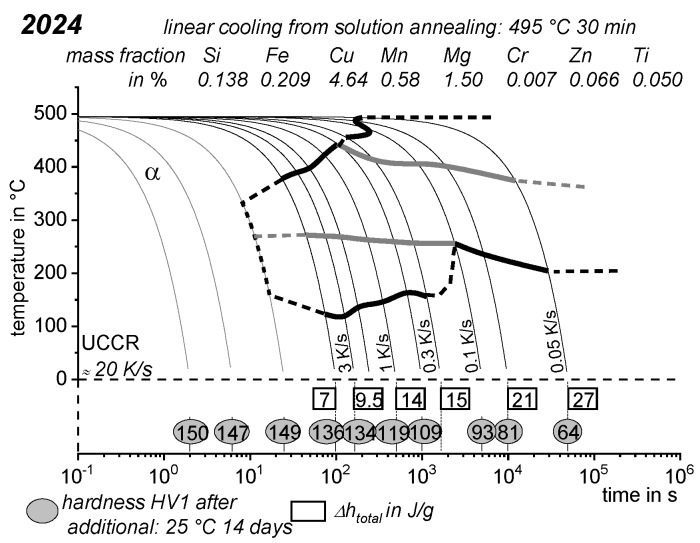
CCP diagram for 2024 [146], revised version. Hardness values obtained after cooling and natural ageing.

**Figure 68:**
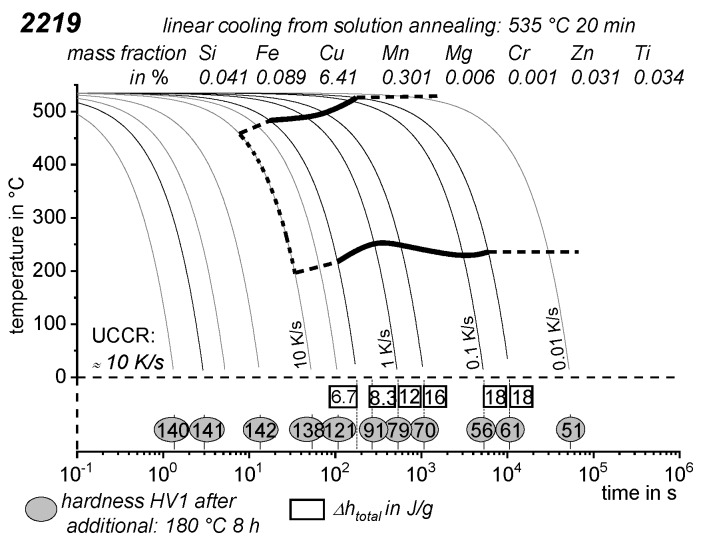
CCP diagram for 2019 [146]. Hardness values obtained after cooling and artificial ageing.

The CCP diagrams for 2024 and 2219 are plotted in Figure 67 and Figure 68, respectively. As the overlap of the reactions is severe in 2219, no distinction of these reactions can be made.

#### 3.2.5. AlSiMg Cast Alloys

The quench sensitivity of AlSiMg cast alloys has been investigated by a range of researchers, and ex situ methods have generally been used to analyse quench-induced precipitation or its detrimental effects on the resulting properties e.g., [45,47,49,53,56]. None of these previous reports used in situ cooling DSC analysis.

**Figure 69:**
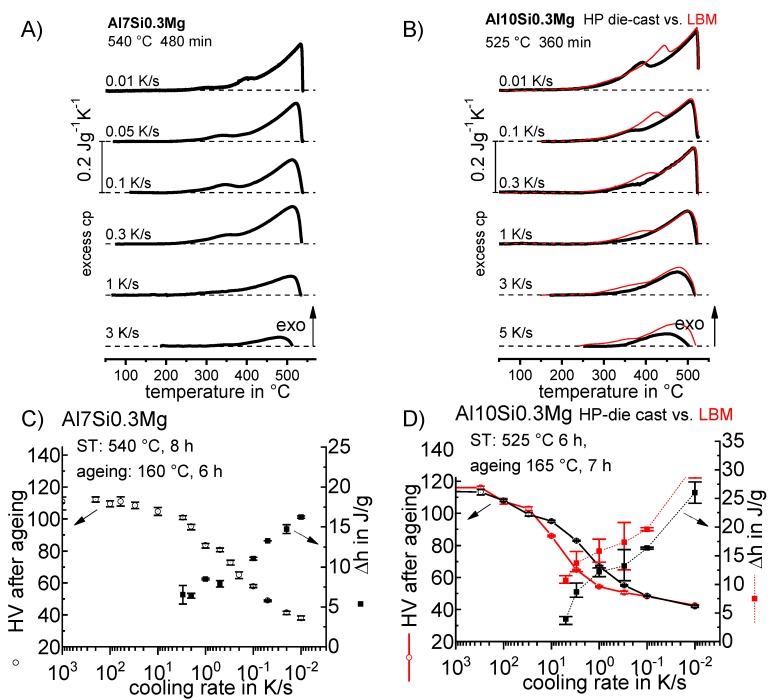
Comparison of three different AlSiMg cast alloys: (**A**) cooling DSC curves for permanent mould-cast Al7Si0.3Mg, [214]; (**B**) cooling DSC curves for two variants of Al10Si0.3Mg—one of the variants was produced by high-pressure die-casting, the second by laser beam melting, [131,215]; (**C**,**D**) values of specific precipitation enthalpy and hardness after ageing for the three cast alloys.

In this work, three variants of AlSiMg cast alloys are assessed, namely permanent mould-cast Al7Si0.3Mg [214], high-pressure die-cast Al10Si0.3Mg [131] and additively manufactured (laser-beam melted, LBM) Al10Si0.3Mg [131]. Figure 69 compares these three cast alloy variants in terms of their DSC cooling curves (A, B) and the resulting precipitation enthalpies and hardness after ageing (C, D). In the same way as for AlMgSi wrought alloys, two main reaction intervals are observed that are strongly overlapped. A broad HTR stretching over more than 100 K with a peak around 450 to 510 °C dominates over the entire cooling rate range considered. It can also be seen that DSC detects a nearly instantaneous onset of precipitation with the start of cooling i.e., nearly no undercooling is required for the reaction to start. Although a detailed experimental analysis of the nature of quench-induced precipitates has not been performed for these cast alloys, it can be assumed that precipitation of Si is important [47], i.e., during relatively slow cooling, part of the dissolved Si will diffuse to the existing eutectic Si [47]. This explains the instantaneous start of precipitation with the onset of cooling, as no separate nucleation is required: Si phase formation can proceed via growth of the existing Si particles. The latter assumption fits perfectly with the findings of instantaneous growth of undissolved β-Mg_2_Si in AlMgSi alloys [148]. In addition to Si, the formation of β-Mg_2_Si is also likely to occur at high temperatures during slow cooling of AlSiMg cast alloys. Ref. [47] reports on the nucleation of β-Mg_2_Si phase particles on eutectic Si particles at slower rates, and the formation of β-Mg_2_Si phase particles within the Al matrix at slightly faster cooling rates. It is likely that the formation of both Si and β-Mg_2_Si contributes to the HTRs. β’-phase particles have also been assumed to precipitate during cooling [47,49]. It is likely that these semi-coherent precursor precipitates form during the LTRs, which have a peak at about 350 °C in the cast alloy variants.

**Figure 70:**
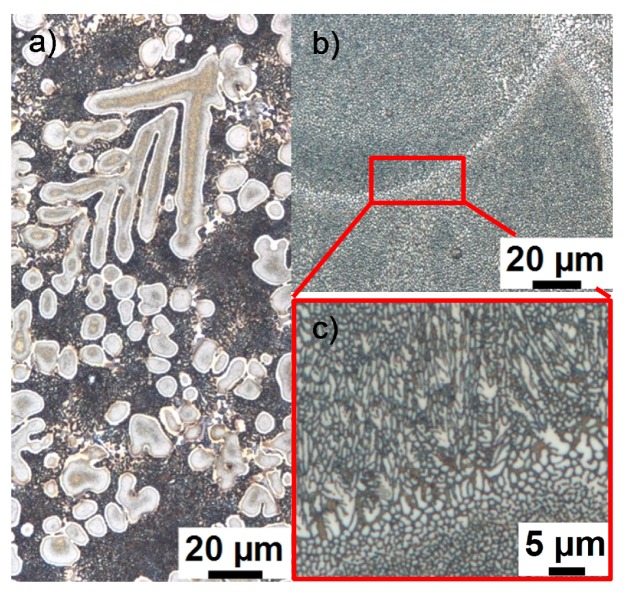
Comparison of the initial microstructures of (**a**) as-cast and (**b**,**c**) as-LBM Al10Si0.3Mg [215].

By comparing the two variants of Al10Si0.3Mg, it can be seen that the die-cast alloy has a higher concentration of Si, while the concentration of Mg is slightly higher in the LBM version, see Table A1 in the Appendix A. A further difference is that the LBM version contains almost no Mn, while the die-cast variant contains a mass fraction of ≈0.4%. It is found that the LBM variant of Al10Si0.3Mg generally produces higher values of specific precipitation enthalpy, and that the loss in hardness potential (i.e., the quench sensitivity) is also higher in this alloy. Since the laser-beam-melting process involves extremely fast cooling rates for solidification, the eutectic like structure of the LBM material is much finer compared to that of the die-cast eutectic (see Figure 70). This much finer eutectic structure is considered to increase the number of available nucleation sites, thus promoting quench-induced precipitation. In line with this, the peak temperature of the LRT is shifted to higher temperatures in the LBM alloy.

**Figure 71:**
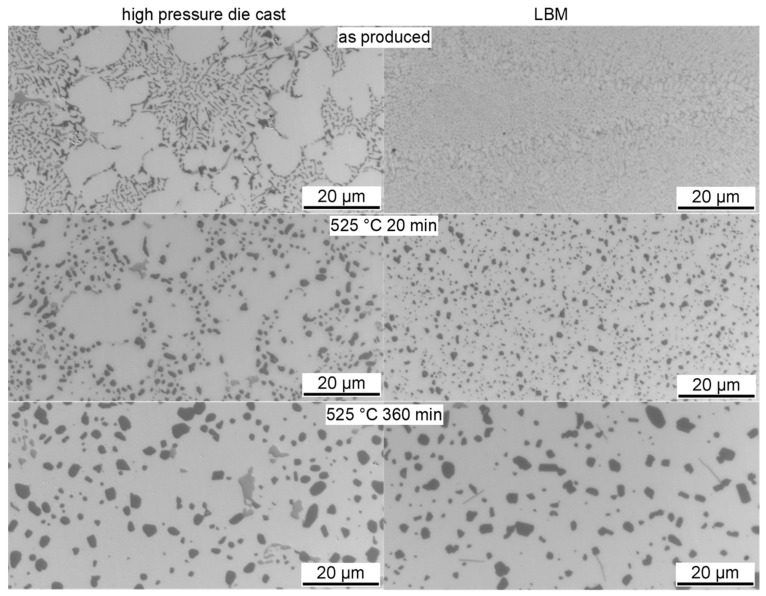
Eutectic structure of Al10Si0.3Mg produced by high-pressure die-cast and LBM, for different soaking times at 525 °C.

No systematic study of the interacting influences of prior solidification rate, solution treatment and alloying element concentration on quench-induced precipitation is currently available for cast alloys. However, there are differences of several orders of magnitude in the solidification rates for the three alloys considered here. These solidification rates are estimated to be around 10^5^ K/s for LBM [216,217], 10^3^ K/s for die casting and 10^1^ K/s for conventional casting [218]. Further, the solidification structure is greatly changed by prolonged soaking at solution temperature. For the AlSiMg cast alloys, it appears that both the alloy composition and the microstructure prior to quenching have an influence on the kinetics of quench-induced precipitation. The microstructure at the end of the solution treatment is a result of the structure after casting (or LBM) and the coarsening caused by the solution treatment. The latter can be seen in Figure 71, which compares the microstructure of the two Al10Si0.3Mg variants in the initial condition and after different soaking durations at solution temperature. This figure shows that the initial difference in the microstructure virtually disappears after six hours of soaking at 525 °C, and similar coarse microstructures are seen for both materials.

**Figure 72:**
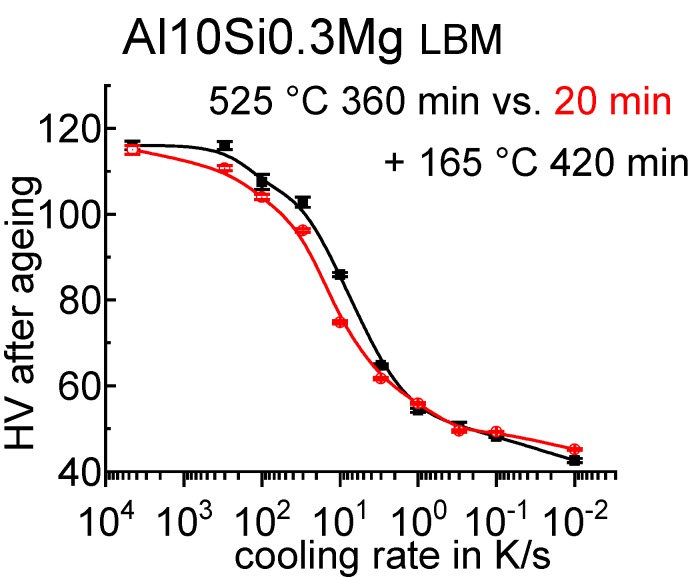
Comparison of hardness after cooling and subsequent ageing for LBM Al10Si0.3Mg.

**Figure 73:**
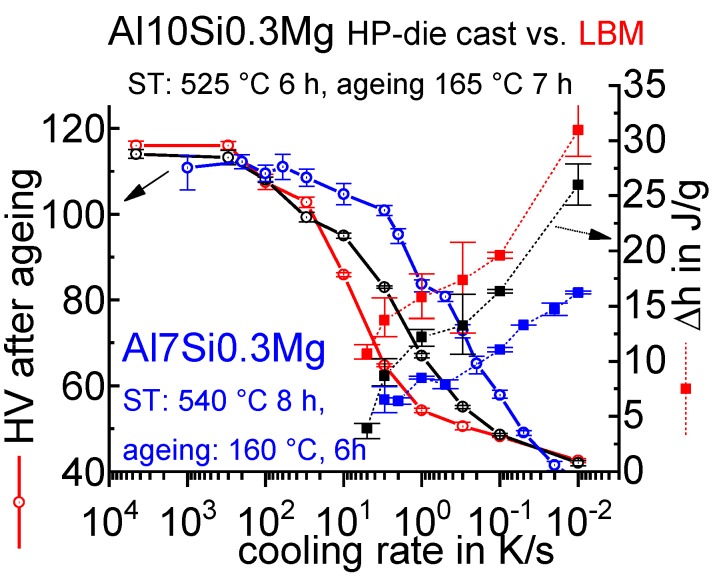
Comparison of specific precipitation enthalpies after cooling and hardness after subsequent ageing for the three AlSiMg cast alloys.

Figure 72 shows a substantial acceleration of quench-induced precipitation and the drop in hardness is shifted to faster cooling rates for a shorter solution treatment in the case of a much finer initial microstructure prior to cooling (compare Figure 71). The UCCR of the finer eutectic structure (after 20 min soaking at 525 °C) appears to be above 1000 K/s. For this reason, the difference in the solution treatments between Al7Si0.3Mg and Al10Si0.3 should be kept in mind when considering Figure 73.

Figure 73 shows that the maximum hardness achievable for the three alloys is very similar (111 to 116 HV1), and this hardness is achieved using high cooling rates in excess of ≈100 K/s [131,214]. This result perfectly fits with those of Ref. [49], in which the maximum hardness after ageing was found to occur after cooling at 110 K/s and above (average cooling rates between 450 and 200 °C). If cooling is performed at lower rates, the age-hardening potential drops significantly for the different alloys, and drops much more quickly for the fast solidified variant (with finer structure) and/or higher concentrated alloys. For instance, at a cooling rate of 2 K/s, which is a typical value for gas cooling, Al7SiMg0.3 still reaches 95 HV after ageing, while the hardness of the LBM variant of Al10Si0.3Mg drops to only around 60 HV1. Dimensional and shape distortion after quenching is very relevant for net-shaped cast products, and quenching rates are therefore often restricted to lower rates. In this case, a permanent mould cast (and less concentrated) alloy could achieve ≈150% of the strength of the LBM alloy. This finding correlates well with the discussion of the AlMgSi and AlZnMgCu wrought alloys (Figure 41 and Figure 56, respectively).

**Figure 74:**
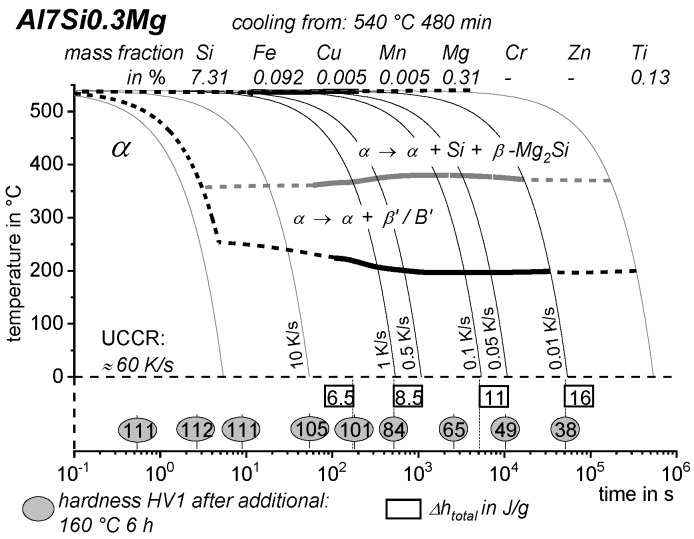
CCP diagram for Al7Si0.3Mg.

**Figure 75:**
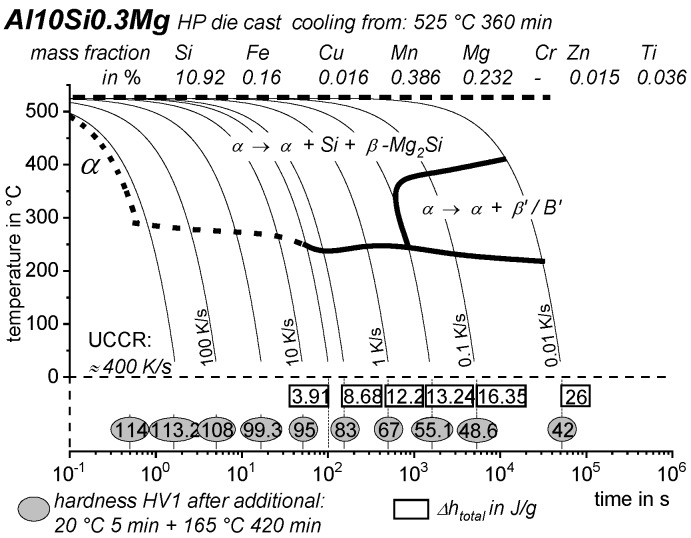
CCP diagram for high-pressure die-cast Al10Si0.3Mg.

**Figure 76:**
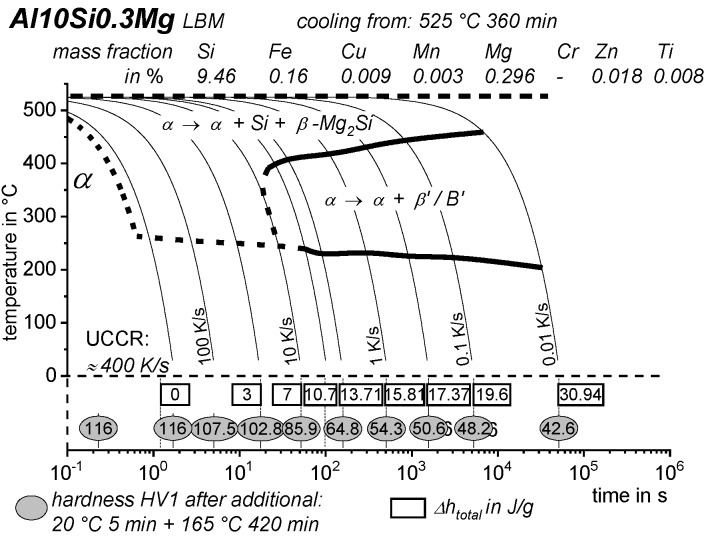
CCP diagram of LBM Al10Si0.3Mg.

CCP diagrams for the cast alloys are given in Figure 74, Figure 75 and Figure 76. As precipitation starts instantaneously with the onset of cooling for all the cast alloys, these CCP diagrams show that the onset of precipitation is the same for any cooling rate. The start of high-temperature precipitation is therefore plotted as a straight line starting at the solution treatment temperature. The reactions that are most likely to be occurring are labelled.

## 4. General Aspects of Quench-Induced Precipitation in Al Alloys

**Figure 77:**
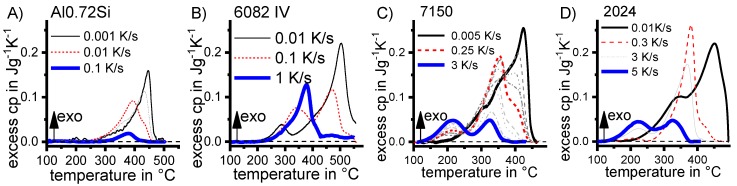
Dynamic behaviour of quench-induced precipitation in four substantially different Al-based alloys, (**A**) Al0.72Si, (**B**) 6082_IV_, (**C**) 7150, (**D**) 2024.

As demonstrated in Figure 77, quench-induced precipitation in the Al alloys investigated here generally falls into different temperature and time (cooling rate) intervals. For most alloys, two or three main reaction intervals can be identified [117,118,119,128,148,161,206]. However, there are several hints of the superposition of multiple reactions beneath the main reaction intervals [116,133]. In addition, a cooling rate that is fast in terms of precipitation for a certain lean alloy may be relatively slow in terms of precipitation for an alloy of higher concentration, and the same holds for the temperature ranges. For instance, the LTR for Al0.72Si (Figure 77A) occurs at a temperature range of about 420 to 300 °C [117], which is almost the same temperature range as for the MTRs of 7150 [119,133], 2024 [146] or 2618 [206] (at least at certain cooling rates).

In a comparison of DSC cooling curves for the four substantially different aluminium alloys shown in Figure 77, it is notable that the calorimetric and kinetic behaviour is to a large extent similar. This is particularly true for a comparison of the alloys 7150 and 2024, which are substantially different in composition but which show highly similar dynamic behaviour of quench-induced precipitation (for different phases).

**Figure 78:**
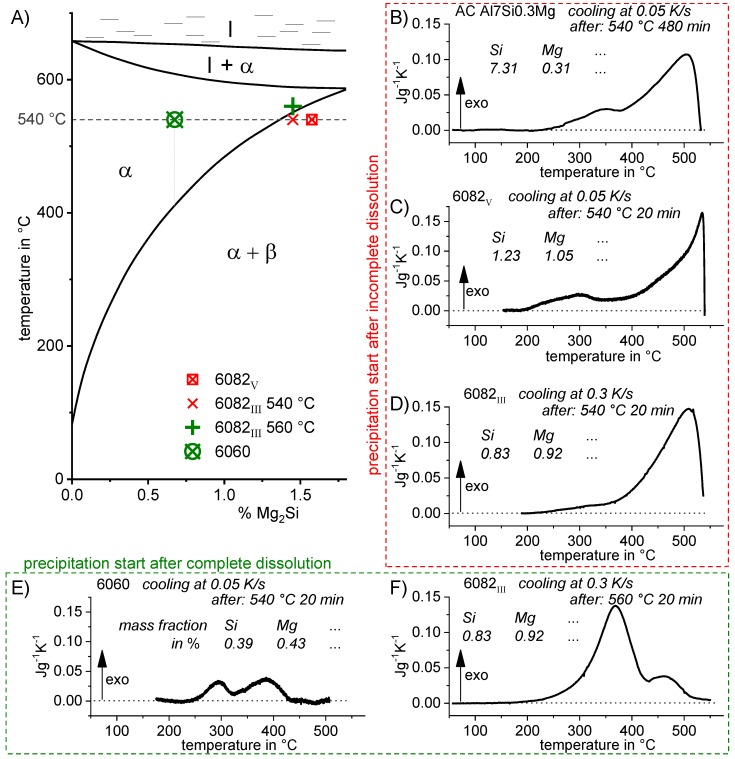
(**A**) Quasi-binary phase diagram for Al-Mg_2_Si (adapted from [5]). (**B**–**D**) DSC cooling curves for three different alloys for which the applied solution treatment resulted in an incomplete dissolution. (**E**,**F**) DSC cooling curves for two different alloys for which the applied solution treatment resulted in complete dissolution. In (**D**,**F**), the same alloy is considered, although cooling started from two different temperatures: 540 °C and 560 °C. The mass fractions of Mg and Si for the different alloys are stated.

For all of the precipitation hardening Al alloys investigated, it holds that if the alloy is cooled sufficiently slowly, quench-induced precipitation occurs at relatively high temperatures [117,118,119,128,148,161]. During the HTRs, essentially stable equilibrium phase particles form in the shape of coarse precipitates with a low aspect ratio, and their dimensions can reach more than 10 µm [117,119,128]. If the grain size of the alloy is considerably larger than this, precipitation will predominantly occur inside the grains [31,118,138,195]. However, quench-induced precipitation also occurs on the grain boundaries [31,118,119,128,138]. In commercial wrought alloys, nucleation of these equilibrium phase particles at high temperatures takes place on relatively coarse primary particles comprised of elements such as Fe, Mn and Si [118,119,128,133,138]. In addition, pre-existing particles from the same phase that were not dissolved during the solution treatment (i. e. stable equilibrium phase particles in the shape of coarse precipitates) can also act as nucleation sites [148]. These undissolved particles start to grow instantly with the onset of cooling. This is obvious from Figure 78, which compares DSC cooling curves for aluminium alloys after incomplete dissolution with those after full dissolution. A comparison of Figure 78D and F is particularly illuminating, as these show DSC cooling curves for the same alloy and the same cooling rate but with different solution treatments. The obvious difference in the precipitation behaviour during cooling is attributed to incomplete dissolution of β-Mg_2_Si after solution treatment at 540 °C for 20 min, while a full solution was achieved in Figure 78F at 560 °C. In Ref. [148], we showed that this incomplete dissolution strongly increases the quench sensitivity and that the UCCR is increased by about a factor of three.

If the reactions at higher temperatures are suppressed to a certain extent by higher cooling rates, another reaction (or multiple reactions) occur(s) at lower temperatures [117,118,119,128,148,206]. The quench-induced MTRs and LTRs of precipitation hardening Al alloys may therefore initially give an increase in the fraction transformed with increasing cooling rates (see the enthalpy change of the LTR in Figure 40). This is also typically related to an increase in the reaction temperatures. The reason for this behaviour is increasing supersaturation (due to the increasing suppression of the HTRs). This increases the driving force for precipitation of the related phases (which typically competes for the same alloying element atoms, like the HTRs).

In general, it can be stated that at lower temperatures, in 6xxx AlMgSi alloys metastable precursor phases particles are precipitated [119,128] while in 7xxx AlZnMg(Cu) alloys quench induced precipitation of the phase η-Mg(Zn,Al,Cu)_2_ occurs. These particles have much larger aspect ratios compared to high-temperature particles, but are still relatively coarse as compared to age hardening precipitates formed during typical artificial ageing treatments. Their dimensions can reach several hundreds of nm in length, and thus they contribute little to direct hardening [119,128]. Nucleation of these quench-induced lower temperature phases takes place mostly on incoherent dispersoids (and on grain boundaries; see for instance Figure 52) [118,119,128,194,195]. The dispersoid particle number density therefore substantially influences quench-induced precipitation and quench sensitivity [119].

For some alloys, quench-induced precipitation at relatively high cooling rates is shown to proceed at relatively low temperatures [119,133,206]. Particularly for AlZnMgCu alloys, it has been found that a thin platelet phase can precipitate at temperatures between 250 and 150 °C [119,143,198]. At even lower temperatures of down to ≈50 °C, cluster formation has been identified [102,103,104]. Although the latter two particle types already provide a considerable direct hardening effect, since the volume fractions generated for these quench-induced particles are small, the total hardening effect is still relatively low [143].

For pure laboratory alloys in the AlSi and AlMgSi sytems, it was shown that one phase can contribute both to the HTR with low aspect ratio precipitates and also to the reactions at lower temperatures with high aspect ratio precipitates [117,132]. It was demonstrated that additions of further alloying elements can cause an additional sequence of potential quench-induced precipitates [119,132,133].

In general, the UCCRs of commercial precipitation-hardening Al alloys range between about 0.5 K/s (6060, [128]) and about 300 K/s for highly concentrated AlZnMgCu alloys [119,129,130,162].

The kinetics of quench-induced reactions are accelerated by an increased concentration of alloying elements [118,119,128]. At medium cooling rates, which are particularly relevant for gas cooling, for instance, more highly concentrated alloys may lose a large amount of their age-hardening potential by quench-induced precipitation. Applying the same gas cooling rates to leaner alloys might still achieve sufficient supersaturation, allowing these alloys to exploit nearly their full age-hardening potential. Leaner alloys might therefore achieve higher hardness and strength after medium-slow cooling, as compared to more highly concentrated alloys. This aspect was demonstrated for AlMgSi and AlZnMgCu wrought and AlSiMg cast alloys (compare Figure 41, Figure 56 and Figure 73, respectively). Particularly for the latter, the applied solution treatment makes a substantial impact, since the coarsening of the eutectic, which increases with soaking duration, influences the density of nucleation sites. In all cases, the lower concentrated alloy after medium-slow cooling at approximately 1 K/s and additional ageing achieved a hardness of about 150% of the highest alloy concentration considered. This aspect has particular technological relevance if the aim is to retain high dimensional stability of the quenched parts, or to limit their residual stresses, which is often associated with reduced cooling rates. The knowledge obtained of the kinetics of quench-induced precipitation now allows us to choose either an appropriate cooling process for a certain fixed alloy and/or an appropriate alloy for a certain fixed cooling process.

## 5. Kinetic Assessment of the DSC Data by Modelling

Diffusion-controlled precipitation reactions can be described by a Starink model based on the extended volume *α_ext_* concept [82]. In this approach, the fraction transformed over time *α(t)* by a precipitation process can be expressed as:

(2) $$\alpha \left(t\right)=\frac{e^{-2\alpha_{ext}}-1}{2\alpha_{ext}}+1$$

(3) $$\alpha_{ext}=\frac{{\left(kt\right)}^{n}}{V_{0}}$$

In Equations (2) and (3), *α_ext_* is the extended fraction transformed, and *k* and *V_0_* are constants under isothermal conditions, which are set to 0.01 and 1 in the example shown in Figure 3. *n* is the reaction exponent, which relates to the type of nucleation and growth. In the example in Figure 3, *n* is set to 1.5, which is valid for heterogeneous nucleation [219], leading to growth in three dimensions of a fixed number of precipitates.

This basic modelling approach was combined with the knowledge gained on nucleation and growth in Ref. [219] and was applied to model the kinetic development of quench-induced precipitation, initially for pure binary AlSi alloys [117]; later, it was extended to AlMgSi and AlZnMg(Cu) alloys [118,119]. The aim of the quench sensitivity model is to predict the volume fractions of the most relevant types of quench-induced precipitates formed during the quench as a function of composition and cooling rate. This enables a kinetic assessment of the large amount of DSC data and verification of the model using these DSC data. For the AlMgSi and AlZnMg(Cu) alloys, the resulting calculated supersaturation was used to predict strengthening after ageing at various cooling rates, via a further extension of the model that describes the formation of hardening precipitates during ageing [118,119].

The new model for quench-induced precipitation has three major components. In the first step, the precipitation during quenching is calculated, while the second step calculates precipitation during subsequent artificial ageing under a T6 treatment, and the third carries out a strength prediction for the T6 condition. The model incorporates some novel theories, and the results generally show excellent accuracy. It is very efficient in terms of computation, as no time stepping is involved. The computational efficiency is achieved by incorporating some simplifications of the highly complex nature of precipitation [118,119], most notably that during cooling, the reactions can be modelled as consecutive reactions.

As input, the model for quench-induced precipitation takes the alloy content of the three or four main and three dispersoid-forming elements of the AlMgSi or AlZnMg(Cu) alloys, respectively. Amongst others, the concentration of dispersoid-forming elements provides an estimate of the number of nucleation sites. In the first step, the phases and their compositions at the solution treatment temperature are calculated assuming thermodynamic equilibrium. Subsequently, the amount of each of the phases formed during quenching is modelled. For AlMgSi alloys, two quench-induced phases are considered, namely β-Mg_2_Si for the HTR and B’-Mg_5_Si_4_Al_2_ for the LTR. For AlZnMg(Cu) alloys, four phases are taken into account: S-Al_2_(Cu,Mg) and β-Mg_2_Si for the HTR, η-Mg(Zn,Al,Cu)_2_ for the MTR and the Y-phase (thin Zn- and Cu-rich platelets [119,143]) for the LTR. In an initial analysis, it was found that taking a fixed composition of η-Mg(Zn,Al,Cu)_2_ does not allow for accurate prediction of the effects of different Mg:Zn ratios. This is as expected, since the η-Mg(Zn,Al,Cu)_2_ phase has a wide range of stable compositions in the AlCuMgZn system. A detailed consideration of the isostructural variants of this phase is therefore included in the full modell (see Ref. [119]).

A feature of the model that was introduced to allow time efficient computation is the assumption that the quench-induced precipitation reactions can be considered to occur consecutively, e.g., the HTR is assumed to be completed before the MTR starts. Although this is not always strictly the case, the DSC cooling curves indicate that is generally a reasonable assumption: the main reactions (HTR, MTR, LRT) generally have an overlap that is limited. To allow calculation of the phase fractions, the novel model for diffusion-controlled precipitation reactions (extended volume concept, [82,219]) is applied to the alloying element that depletes fastest. Thus, the extended volume fraction approach was used for a constant cooling rate:

(4) $$\alpha_{ext}={\left(\frac{k}{\beta }\right)}^{n}$$

Here, *n* is the reaction exponent and *β* is the cooling rate, while *k* is a factor that incorporates the alloy composition, the solvus temperature of the relevant phase and the temperature. For full details, see [118,119]. The extended volume concept leads to a novel expression for the fraction transformed, *α*, by combining Equation (2) [82] with Equation (4), now allowing for the consideration of linear cooling.

(5) $$n=N_{dim}\;g+B$$

In Equation (5), *N_dim_* represents the dimensionality of growth, while *g* is ½ for diffusion-controlled growth and *B* is either zero (if nucleation is completed early in the reaction) or one (in the case of continuous nucleation).

**Figure 79:**
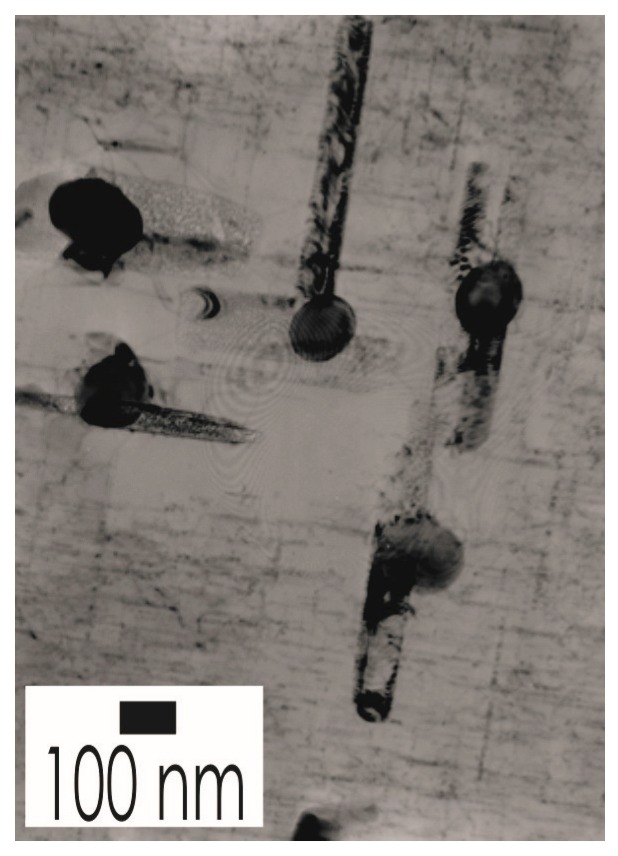
TEM-BF image of quench-induced B’-rods after air cooling (≈1 K/s) in a 6082 alloy (courtesy Shuncai Wang, University of Southampton, UK & Paul Rometsch, published in [118]).

This novel model thereby takes into consideration soft impingement for the diffusion fields around different quench-induced particles. The necessity of taking soft impingement into account is evident from the theory and analysis of transformation data [57,75,82], and can be illustrated using Figure 79, which shows quench-induced B’ particles in an alloy 6082 [118]. The sample was cooled in air from solution treatment and subsequently artificially aged. The quench-induced B’ - particles are nucleated on Mn,Fe-rich dispersoids. The fine needle-shaped β’’ age-hardening precipitates clearly indicate the overlapping diffusion fields of the quench-induced B’-rods, as there are clear zones that are free of age-hardening β’’ precipitates (due to the severe depletion of solutes and potentially vacancies from overlapping diffusion fields of the quench-induced B’-rods).

The model also considers the effect of the interfacial energy of the dispersoid particles in the AlZnMg alloys on the nucleation rate. To this end, we first consider that the interfaces of Al_3_Zr and Al_6_(Mn,Fe) are known to be semi-coherent with the matrix, whilst the Cr-containing dispersoids are generally considered to be incoherent. The difference in interfacial energy between a semi-coherent precipitate (misfit ~1%) and an incoherent interface is typically a factor of about three to eight [220]. In line with this, we take the efficiency of Cr-containing dispersoids in nucleating the η-Mg(Zn,Al,Cu)_2_ precipitates to be a factor of five larger than that of the semi-coherent precipitates. This assumption has no noticeable effect on the accuracy of the model for the present alloys, since nucleation of quenched-in precipitates is dominated by Al_3_Zr dispersoids, grain/subgrain boundaries, and, to a lesser extent, Mn-containing dispersoids. However, it would influence the accuracy of prediction for data on Cr-containing alloys.

The model of quench-induced precipitation requires only one fitting parameter per reaction/phase, and can cover any composition and a very wide range of cooling rates.

The main model outputs that are directly amenable to comparison with available data are the predictions for the total specific precipitation enthalpy after cooling and hardness after additional ageing. These comparisons are presented in Figure 40 and Figure 41 for AlMgSi alloys and in Figure 55 for AlZnMg(Cu) alloys. It is clear that the model works well for a wide range of alloys.

**Figure 80:**
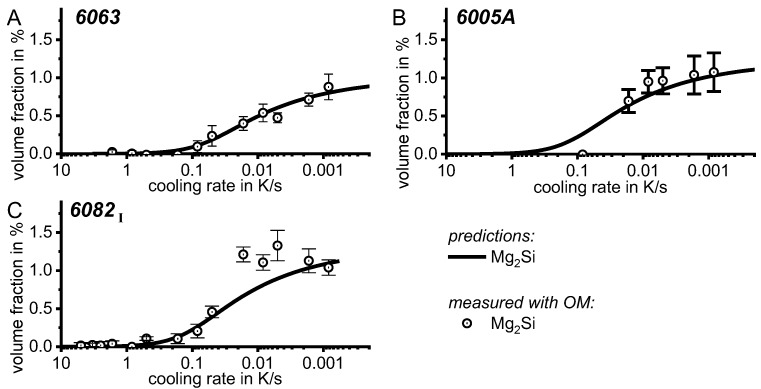
Comparison of measurements and predictions of the volume fractions of Mg_2_Si precipitated during cooling in three different AlMgSi alloys [118].

The model was also validated through comparison with the measured volume fractions of quench-induced β-Mg_2_Si precipitation for different AlMgSi alloys (see Figure 80). It can be observed that using this data, the model also provides an excellent fit to the measured values. Another validation of the predictions of the model is presented in Figure 58, which shows the specific precipitation enthalpy of 7150. In this case, experimental data were obtained one year after the model was derived. The excellent correspondence is further evidence that the model is genuinely predictive for unseen data.

## 6. Application of the Derived DSC Methods to Other Alloy Systems

The methodology for heating and cooling DSC described in Section 2 has been applied to analyse a range of solid-solid phase transformations in other metallic alloy systems. For instance, quench-induced precipitation in magnesium alloys [123] and the tempering of steels have been analysed [221,222]. Recently, the DSC method for the analysis of solid-solid phase transformation kinetics was successfully adapted for use with high-temperature alloys up to 1100 °C [122]. The in situ analysis of quench-induced precipitation in precipitation hardening martensitic steels [122,131,215], and Ni-based alloys [122] has successfully been demonstrated.

**Figure 81:**
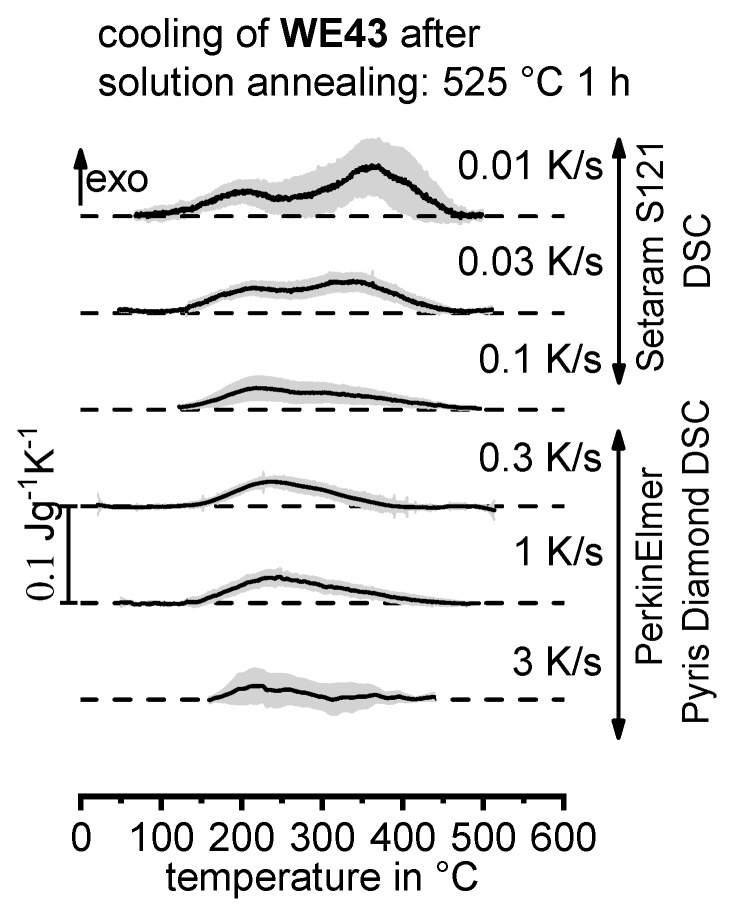
DSC cooling curves for age-hardening Mg alloy WE43 [123].

**Figure 82:**
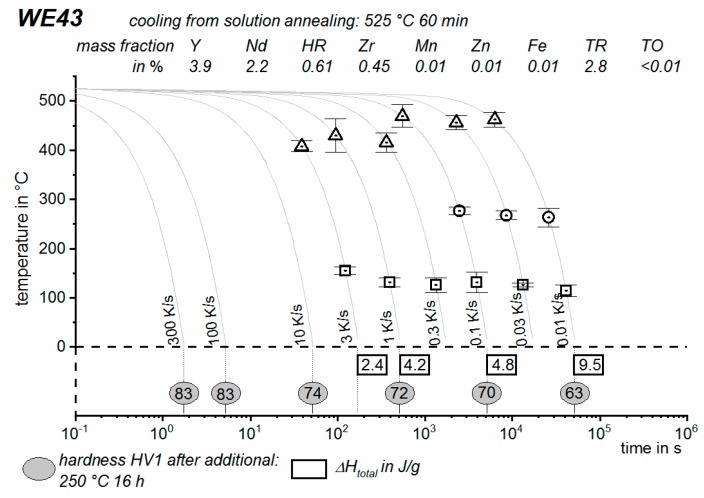
Continuous cooling precipitation diagram of age-hardening Mg alloy WE43 [123].

The strength of a range of magnesium alloys is increased by precipitate strengthening, achieved through post-quench ageing [5]. The age-hardening potential of precipitation-hardening Mg alloys can be reduced by quench-induced precipitation. Until recently, the analysis of precipitation reactions by DSC was restricted to temperatures well below the solution temperature, mainly due to the low eutectic melting temperature in the system Mg-Al system, causing localised melting at the interface between Mg alloy sample and Al crucible. To solve this limitation recently the DSC methods have been adapted by using graphite crucibles to allow the kinetic analysis of precipitation and dissolution reactions in Mg alloys up to solution annealing temperatures [123]. An example of DSC cooling curves for the WE43 Mg-Y-Re alloy from solution treatment temperature is shown in Figure 81, and these DSC curves show a high similarity to the DSC cooling curves for Al alloys. DSC has been shown in recent work to allow the derivation of a continuous cooling precipitation diagram for Mg alloys [123]. The CCP diagram for the WE43 Mg-Y-RE alloy is presented in Figure 82. The UCCR of this alloys is in the range of 10 to 100 K/s [123].

**Figure 83:**
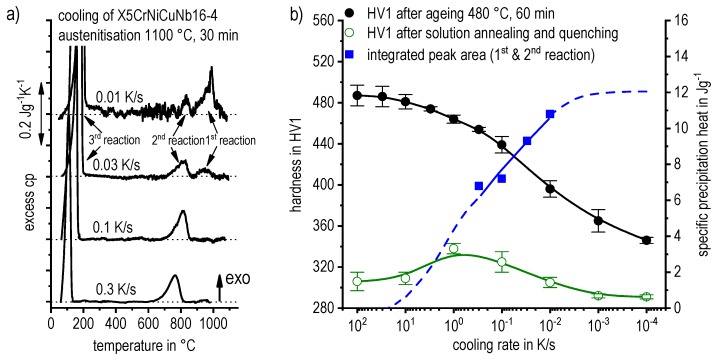
X5CrNiCuNb16-4 (**a**) Selected DSC cooling curves of X5CrNiCuNb16-4 after austenitisation at 1100 °C, 30 min. The DSC peaks between about 1000 °C and 600 °C indicate the quench-induced precipitation of Cu-rich particles, while the strong peak below about 200 °C corresponds to the martensitic transformation. (**b**) Hardness as a function of the cooling rate in the quenched and in the quenched and aged conditions, adapted from [122].

In the martensitic precipitation hardening of steels such as X5CrNiCuNb16-4, strengthening is caused by the precipitation of fine Cu-rich particles [223,224,225,226]. In terms of cooling from solution treatment, the quench-induced precipitation of Cu-rich phases has thus far been disregarded, since the available continuous time-temperature transformation diagrams for precipitation hardening steels were recorded by dilatometry. The available continuous time-temperature-transformation diagrams therefore neglect precipitation, and only the martensitic transformation has been analysed in work using dilatometry [227]. Recent work [122,131] covering cooling DSC on X5CrNiCuNb16-4 clearly reveals quench-induced precipitation and its negative effect on the hardness after ageing (see Figure 83). In a similar way to aluminium alloys, precipitation occurs in two different temperature regimes and the UCCR for this steel is about 30 K/s.

**Figure 84:**
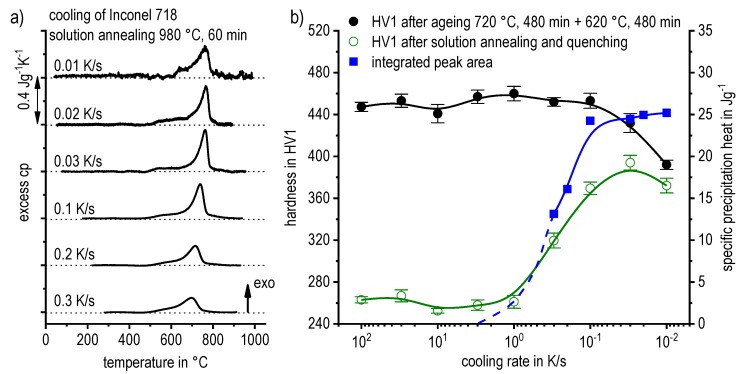
(**a**) Continuous DSC cooling curves of Inconel 718; (**b**) hardness profile and specific precipitation heat depending on the cooling rate [122].

Ni-based alloys can also benefit from precipitation strengthening. Recently the precipitation behaviour of Inconel 718 (a Ni-17Cr-17Fe-5Nb-3Mo alloy, mass fraction in %) during cooling from solution treatment was investigated [122]. Figure 84a shows continuous DSC cooling curves for this alloy after solution annealing at 980 °C for 60 min, and Figure 84b illustrates the hardness and the specific precipitation heat, which depend upon the cooling rate. Linear cooling (e.g., cooling in the DSC) involves a two-stage precipitation process that is kinetically suppressed by an increase in the cooling rate. It can be seen from Figure 84b that there is a substantial direct hardening effect of quenched induced precipitates. This effect is known to occur in Ni-based alloys in an extend allowing technological application for strengthening [228,229,230]. Compared to Al alloys, this effect is much stronger due to the substantially larger fractions of alloying elements involved in precipitation. The UCCR in terms of hardness after ageing is in the range of just 0.1 K/s.

## 7. Conclusions

### 7.1. In Situ DSC Analysis of Solid-Solid Phase Transformations in Precipitation Hardening Alloys

- In the past 10 to 15 years, the dynamic range of in situ DSC on age-hardening alloys has been substantially extended, particularly in terms of continuous cooling experiments. A range of cooling rates from about 3 × 10^−4^ to 3 K/s can now be applied in a reliable analysis method using direct, in situ DSC experiments. Based on a typical temperature interval for the cooling of light metal alloys from solution treatment, this is equivalent to cooling durations ranging from several weeks up to a few minutes.
- By combining direct, in situ DSC measurements with indirect DSC measurements where no direct measurement is possible, the accessible cooling rate range is extended to 10 orders of magnitude, from about 10^−5^ to 10^5^ K/s. When applied to the cooling of aluminium alloys from solution annealing, this corresponds to cooling over several months down to cooling within several hundredths of a second.
- The key features which make these in situ DSC analyses of solid-solid phase transformation possible are as follows:
  ○ Measurement and evaluation of the specific excess heat capacity;
  ○ Taking great care with the accuracy of the DSC zero level; and
  ○ Consideration of a large dynamic range of heating or cooling rates as well as the analysed scales of microstructural changes.
- DSC provide crucial information for choosing appropriate heat treatment parameters for age-hardening alloys based on in situ experiments. This holds for the solution treatment (heating rates, solution temperature and soaking duration) and particularly for quenching.
- The new DSC methods have also been successfully adapted and applied to the analysis of solid-solid phase transformations in other precipitation hardening alloy systems, including Mg alloys, precipitation-hardening martensitic steels and Ni-based alloys.

### 7.2. Continuous Heating and Solution Annealing

- DSC heating curves of precipitation hardening alloys for a specific initial condition allow us to judge whether dissolution or precipitation reactions are predominant at certain times and temperatures.
- Interpretations of DSC heating curves for precipitation-hardening alloys are often found to be challenging. The strong superposition of opposite endo- and exothermic reactions (dissolution/precipitation) make exact interpretations difficult; that is, single DSC peaks, their peak positions and peak areas are not necessarily equal to the maximum intensity of the underlying microstructural reaction.
- Nevertheless, as a general rule, it can be derived that:
  ○ Any diffusion-controlled reaction is increasingly suppressed with increasing heating rate. Suppression of precipitation reactions seems to be easier than suppression of dissolution reactions. Consequently, at sufficiently high heating rates, only dissolution reactions will occur.
  ○ Any diffusion-controlled reaction shifts to higher temperatures with increasing heating rate. Increasing the heating rate by a factor of 100 typically causes shifts on the order of more than 100 K.
- If the alloy and heating rate specific solvus temperature is exceeded, the total integral of the DSC heating curve reveals the enthalpy level of the initial alloy state. This enthalpy level provides information about the thermal stability of the initial condition, and is higher for more stable conditions.
- For aluminium alloys undergoing different initial heat treatment states such as “as quenched”, T4, T6 or T7, DSC heating curves show severe differences at temperatures below about 300 °C. Above this temperature, the differences are typically small.
- DSC heating is able to identify appropriate temperature ranges for solution treatment, particularly at slow heating rates, since for slower heating the alloy- and heating-rate-specific solvus temperature can be identified. At very slow heating rates, the latter will be close to the equilibrium solvus temperature. Additional experiments can be performed to check whether a complete dissolution of the major alloying elements has been achieved. These additional experiments include isothermal DSC during soaking within the previously identified temperature range.

### 7.3. Continuous Cooling and Analysis of Quench-Induced Precipitation

- During cooling, only exothermic precipitation occurs, making interpretation of DSC cooling curves easier than for DSC heating curves. However, in most cases, several different reactions overlap, and their deconvolution may be challenging. Multiple reactions occurring sequentially were detected for all alloys investigated.
- The nature and kinetics of quench-induced precipitates in age-hardening Al-alloys were analysed for 27 different alloys, and both differences and similarities were identified between different alloying systems:
  ○ Quench-induced precipitation occurs at both, grain boundaries and predominantly inside grains. The latter particularly holds for grain sizes above several tens of µm.
  ○ Nucleation of quench-induced precipitation generally occurs on existing crystal defects such as grain boundaries, primary precipitates and dispersoids.
  ○ At high temperatures (≈500–350 °C), the stable equilibrium phases of the alloy system precipitate as coarse particles with a low aspect ratio (aspect ratios = length/ (thickness or diameter) is about 1 to 5), and nucleation occurs on coarse primary particles.
  ○ At medium temperatures (≈350–200 °C) in the AlMgSi system, precipitation of MgSi precursor phases occurs as rods with aspect ratios of about 10. In the AlZnMg(Cu) system, precipitation of the η-Mg(Zn,Al,Cu)_2_ phase occurs as plates with aspect ratios of up to 10. In both alloy systems, quench-induced precipitation at medium temperatures nucleates on dispersoids.
  ○ At low temperatures (≈250–150 °C) in the AlZnMg(Cu) system, thin plates enriched with Cu and Zn were detected as quench-induced precipitation. At very low temperatures (≈150–50 °C) in the AlZnMg(Cu) and AlMg(Cu) systems, quench-induced precipitation of clusters was revealed.
- The major aspects influencing the quench sensitivity are:
  ○ The concentrations of the main and dispersoid-forming alloying elements;
  ○ The density of nucleation sites (coarse primary particles, particularly dispersoids, grain boundaries, undissolved secondary phases, eutectic structure), i.e., the initial microstructure initial prior to the start of cooling;
  ○ Concentrations and nucleation sites form the major reason for the significant alloy batch sensitivity in terms of the kinetics of quench-induced precipitation. For instance, for different batches of 6082, the upper critical cooling rate (UCCR) might vary by up to a factor of 10;
  ○ Moreover, the result of the solution treatment in terms of complete or incomplete dissolution influences the quench sensitivity; incompletely dissolved remaining particles can instantly start to grow with the onset of cooling (with no nucleation required, and no undercooling). This can increase the UCCR by a factor of three.
- The most highly concentrated Al alloys have a UCCR in the range of several hundreds of K/s. Lean commercial alloys such as 6060 may have a UCCR as low as 0.5 K/s, and for pure laboratory alloys (with a substantially reduced number of nucleation sites), this may be even lower (e.g., pure binary Al0.26Si about 0.02 K/s).
- A systematic methodology for the analysis, evaluation and construction of continuous cooling precipitation diagrams for precipitation hardening alloys and guidelines for reading these were derived. This significantly advances the state of the art in the heat treatment of aluminium alloys.
- It was shown that alloys with the highest alloying element contents are able to achieve the highest hardness if the necessary high upper critical cooling rate is reached. However, if the technological application requires slower cooling (for instance due to thick products or to keep distortion low), a lower concentration of alloying elements can lead to higher hardness due to quench-induced precipitation kinetics and the related loss of age-hardening potential.
- A kinetic assessment of the specific precipitation enthalpy data allowed us to model the kinetic development of quench-induced precipitation and verification of the model using these DSC data. In addition, the resulting strength and hardness after subsequent artificial ageing were modelled for AlMgSi and AlZnMg(Cu) alloys.

## Figures and Tables

**Table 1:** Quench-induced phases in AlMgSi alloys.

	Quench-Induced Phases	Alloy	Precipitation Temperature Range from DSC	Particle Morphology and (Aspect Ratio: Length/Thickness)	Nucleation on	Reference
**Stable phases**	β-Mg_2_Si, fcc	6005A	HTR	Plates (3)	Coarse primary Fe, Si, Mn-rich particles (inside the grain and on grain boundaries) or on undissolved Mg_2_Si [148]	[128,138]
		Al0.6Mg0.8Si	HTR & LTR	Plates (?) and needles? (100)	?	This work
	Si, diamond cubic	6005A	HTR (LTR?)	Polygonal particles (close to 1) Potentially plates	?	This work
**Metastable phases**	β′-Mg_9_Si_5_, hexagonal	6005A Al-0.67Si-0.84Mg-0.35Mn-0.25Fe (mass %)	LTR	Rods (19)	Dispersoids	[118] [180]
		Al0.6Mg0.8Si Al0.8Mg0.6Si	LTR	Rods/plates?	?	This work
	B’-Mg_5_Si_4_Al_2_, hexagonal	6005A 6082	LTR	Rods (10)	Dispersoids (potentially on grain boundaries)	[118,128]
	U1-MgAl_2_Si_2_ trigonal	Al0.8Mg0.6Si	LTR	Rods/plates?	?	This work

**Table 2:** Quench-induced phases in AlZnMg(Cu) alloys.

	Quench-Induced Phases	Alloy	Precipitation Temperature Range from DSC	Particle Morphology (Aspect Ratio)	Nucleation	Reference
Stable phases	S-Al_2_CuMg	7150	HTR	(<5?)	On coarse primary Al_7_CuFe and grain boundaries	[119,133,189]
	β-Mg_2_Si	7020	HTR	Plates (?)	(?)	
	η-Mg(Zn,Al,Cu)_2_	7150	MTR	Polygonal plates (<10)	On dispersoids [31,194] and grain boundaries [119,189]	
		7020	MTR	Plates (?)	(?)	
Metastable phases	Y-phase (enriched in Zn, Cu)	7150	LTR	Thin plates (≈100)	Presumably on vacancy clusters or dislocations	[119,143]
	Cluster	7449	vLTR			[103,104]

## Appendix A

**Table A1:** Atomic fractions (bold) and mass fractions of alloying elements for the considered alloys.

		Mass Fraction in %				Atomic Fraction in %					
**wrought alloys**											
product type		**Si**	**Fe**	**Cu**	**Mn**	**Mg**	**Cr**	**Zn**	**Ti**	**Zr**	**Al**
**6xxxx - AlMgSi**											
AA6060	commercial extrusion	0.4	0.2	0.01	0.02	0.44	0.001	0.01	0.009		98.9
	thin walled profile	**0.38**	**0.10**	**0.00**	**0.01**	**0.49**	**0.00**	**0.00**	**0.01**		**99.0**
AA6063	commercial extrusion	0.5	0.19	0.02	0.03	0.47	0.005	0.03			98.7
	closed profile	**0.48**	**0.09**	**0.01**	**0.01**	**0.52**	**0.00**	**0.01**			**98.9**
AA6005A	commercial extrusion	0.68	0.2	0.01	0.11	0.57	0.04	0.01			98.4
	open profile	**0.65**	**0.10**	**0.00**	**0.05**	**0.63**	**0.02**	**0.00**			**98.5**
Al0.6Mg0.8Si	laboratory extrusion	0.79	<0.001	<5 ppm	<5 ppm	0.59	<5 ppm	<10 ppm			98.6
	rectangular bar 20 mm × 50 mm	**0.76**				**0.65**					**98.6**
Al0.8Mg0.6Si	laboratory extrusion	0.61	<0.001	<5 ppm	<5 ppm	0.8	<5 ppm	<10 ppm	<2ppm		98.6
	rectangular bar 20 mm × 50 mm	**0.59**				**0.89**					**98.5**
AA6082 I	commercial extrusion	0.733	0.22	0.05	0.48	0.609	0.003	<0.01	0.008		97.9
	rod Ø30 mm	**0.71**	**0.11**	**0.02**	**0.24**	**0.68**	**0.00**	**0.00**	**0.00**		**98.2**
AA6082 II	commercial extrusion	0.94	0.19	0.0452	0.575	0.757	0.0803	0.198	0.0245		97.2
	thin walled open profile	**0.91**	**0.09**	**0.019**	**0.28**	**0.84**	**0.042**	**0.082**	**0.014**		**97.7**
AA6082 III		0.83	0.38	0.06	0.48	0.92	0.03	0.01	0.02		97.3
	commercial 10 mm sheet	**0.80**	**0.18**	**0.03**	**0.24**	**1.03**	**0.02**	**0.00**	**0.01**		**97.7**
AA6082 IV	commercial extrusion	1019	0.435	0.106	0.476	0.64	0.04	0.096	0.034		97.0
	rod Ø30 mm	**1.15**	**0.21**	**0.05**	**0.24**	**0.71**	**0.02**	**0.04**	**0.02**		**97.6**
AA6082 V	commercial extrusion	1.23	0.2	0.09	0.65	1.05	0.2	0.05	0.03		96.5
	rod Ø70 mm	**1.19**	**0.1**	**0.038**	**0.32**	**1.17**	**0.104**	**0.021**	**0.017**		**97.0**
**7xxxx - AlZnMg(Cu,Si)**		**Si**	**Fe**	**Cu**	**Mn**	**Mg**	**Cr**	**Zn**	**Ti**	**Zr**	**Al**
AA7020	laboratory extrusion	0.11	0.17	0.04	0.15	1.19	0.11	4.37	0.03	0.14	93.69
	rod Ø30 mm	**0.11**	**0.08**	**0.02**	**0.06**	**1.36**	**0.06**	**1.85**			**96.42**
AA7021		<0.25	<0.4	<0.16	<0.1	1.6–2.1	<0.05	6.0–6.8	<0.1	<0.18	
	commercial 2 mm sheet										
AA7055		0.03	0.03	2	<0.01	2	<0.01	8.12		0.12	87.7
	laboratory hot rolled plate	**0.03**	**0.02**	**0.9**		**2.36**		**3.56**		**0.04**	**93.09**
AA7150	commercial thick plate,	0.02	0.05	2.04	0.04	2.15	<0.01	6.33	0.01	0.12	89.24
	centre layer	**0.02**	**0.03**	**0.91**	**0.02**	**2.51**		**2.74**		**0.04**	**93.73**
AA7150	commercial thick plate,	0.02	0.05	2.04	0.04	2.15	<0.01	6.33	0.01	0.12	89.24
	surface layer	**0.02**	**0.03**	**0.91**	**0.02**	**2.51**		**2.74**		**0.04**	**93.73**
AA7085		0.07	0.03	2.06	<0.01	1.46	<0.01	8.16		0.12	88.1
	laboratory hot rolled plate	**0.07**	**0.02**	**0.93**		**1.72**		**3.58**		**0.04**	**93.64**
AA7085_lowCu_		0.12	0.02	0.91	<0.01	1.37	<0.01	7.82		0.12	89.64
	laboratory hot rolled plate	**0.12**	**0.01**	**0.41**		**1.6**		**3.4**		**0.04**	**94.42**
AA7075 I	commercial extrusion	0.17	0.22	1.57	0.11	2.08	0.22	5.95	0.05		89.63
	rod Ø35 mm	**0.17**	**0.11**	**0.698**	**0.06**	**2.42**	**0.12**	**2.57**	**0.029**		**93.83**
AA7075 II		0.19	0.11	1.5	0.04	2.64	0.18	3.06	0.04		89.24
	commercial 2 mm sheet	**0.19**	**0.06**	**0.67**	**0.02**	**3.06**	**0.10**	**2.61**	**0.024**		**93.27**
AA7049A	commercial extrusion	0.25	0.35	1.9	0.2	2.9	0.22	8.2			86.0
	rod Ø50 mm	0.26	0.18	0.86	0.1	3.43	0.12	3.6	–	–	91.45
**2xxxx – AlCu(Mg)**		**Si**	**Fe**	**Cu**	**Mn**	**Mg**	**Cr**	**Zn**	**Ti**	**Zr**	**Al**
AA2219		0.041	0.089	6.41	0.301	0.0057	<0.001	0.031	0.034		93.1
	commercial 9mm sheet	**0.04**	**0.045**	**2.83**	**0.15**	**0.007**	**<0.001**	**0.013**	**0.020**		**96.9**
AA2024	commercial extrusion	0.138	0.209	4.64	0.58	1.5	0.007	0.066	0.05		92.8
	rod Ø30 mm	**0.14**	**0.10**	**2.3**	**0.29**	**1.72**	**0.00**	**0.03**	**0.03**		**95.7**
**Al-Si binary**		**Si**	**Fe**	**Cu**	**Mn**	**Mg**	**Cr**	**Zn**	**Ti**	**Zr**	**Al**
Al0.26Si		0.26	<5 ppm	<4 ppm	<3 ppm	<1 ppm	<3 ppm	–	20 ppm		99.74
	laboratory 20 mm plate	**0.25**									**99.75**
Al0.72Si		0.72	<5 ppm	<1 ppm	<3 ppm	<1 ppm	<3 ppm	14 ppm	11 ppm		99.28
	laboratory 20 mm plate	**0.69**									**99.31**
**cast alloys**											
**AlSiMg cast**		**Si**	**Fe**	**Cu**	**Mn**	**Mg**	**Cr**	**Zn**	**Ti**	**Zr**	**Al**
Al7Si0.3Mg		7.31	0.092	0.005	0.005	0.31			0.13		92.1
	mould cast	**7.05**	**0.04**	**0.002**	**0.002**	**0.35**			**0.07**		**92.5**
Al10Si0.3Mg		10.92	0.16	0.02	0.39	0.23		0.02	0.04		88.2
	high pressure die cast	**10.56**	**0.08**	**0.01**	**0.19**	**0.26**		**0.01**	**0.02**		**88.9**
Al10Si0.3Mg		9.46	0.16	0.01	0.003	0.30		0.02	0.01		90.0
	laser beam molten bar	**9.13**	**0.08**	**0.004**	**0.001**	**0.33**		**0.01**	**0.00**		**90.4**
